# Continent-Wide Decoupling of Y-Chromosomal Genetic Variation from Language and Geography in Native South Americans

**DOI:** 10.1371/journal.pgen.1003460

**Published:** 2013-04-11

**Authors:** Lutz Roewer, Michael Nothnagel, Leonor Gusmão, Veronica Gomes, Miguel González, Daniel Corach, Andrea Sala, Evguenia Alechine, Teresinha Palha, Ney Santos, Andrea Ribeiro-dos-Santos, Maria Geppert, Sascha Willuweit, Marion Nagy, Sarah Zweynert, Miriam Baeta, Carolina Núñez, Begoña Martínez-Jarreta, Fabricio González-Andrade, Elizeu Fagundes de Carvalho, Dayse Aparecida da Silva, Juan José Builes, Daniel Turbón, Ana Maria Lopez Parra, Eduardo Arroyo-Pardo, Ulises Toscanini, Lisbeth Borjas, Claudia Barletta, Elizabeth Ewart, Sidney Santos, Michael Krawczak

**Affiliations:** 1 Institute of Legal Medicine and Forensic Sciences, Department of Forensic Genetics, Charité—Universitätsmedizin Berlin, Berlin, Germany; 2 Cologne Center for Genomics, University of Cologne, Cologne, Germany; 3 Institute of Medical Informatics and Statistics, Christian-Albrechts University, Kiel, Germany; 4 Institute of Molecular Pathology and Immunology, University of Porto, Porto, Portugal; 5 Universidade Federal do Pará, Laboratório de Genética Humana e Médica, Belém, Pará, Brazil; 6 Universidad de Buenos Aires, Facultad de Farmacia y Bioquimica, Servicio de Huellas Digitales Geneticas, Buenos Aires, Argentina; 7 Department of Psychiatry and Psychotherapy, Charité—Universitätsmedizin Berlin, Berlin, Germany; 8 Department of Forensic Medicine, University of Zaragoza, Zaragoza, Spain; 9 Science and Technology Department, Ministry of Public Health, Quito, Ecuador; 10 Laboratorio de Diagnósticos por DNA, Instituto de Biologia, Universidade do Estado do Rio de Janeiro, Rio de Janeiro, Brazil; 11 GENES Ltda., Laboratorio Genetica Forense y Huellas Digitales del DNA, Medellín, Colombia; 12 Instituto de Biología, Universidad de Antioquia, Medellín, Colombia; 13 Unitat d'Antropologia, Departamento de Biologia Animal, Facultat de Biologia, Universitat de Barcelona, Barcelona, Spain; 14 Laboratorio de Genetica Forense, Departamento de Toxicología y Legislación Sanitaria, Facultat de Medicina, Universidad Complutense de Madrid, Madrid, Spain; 15 PRICAI–Fundación Favaloro, Buenos Aires, Argentina; 16 Laboratorio de Genetica Molecular, Unidad de Genetica Medica, Facultad de Medicina, Universidad del Zulia, Maracaibo, Venezuela; 17 Laboratorio de Genética Humana, Facultad de Ciencias Biológicas, UNMSM–Universidad, Nacional Mayor de San Marcos, Lima, Peru; 18 Institute of Social and Cultural Anthropology, University of Oxford, Oxford, United Kingdom; Dartmouth College, United States of America

## Abstract

Numerous studies of human populations in Europe and Asia have revealed a concordance between their extant genetic structure and the prevailing regional pattern of geography and language. For native South Americans, however, such evidence has been lacking so far. Therefore, we examined the relationship between Y-chromosomal genotype on the one hand, and male geographic origin and linguistic affiliation on the other, in the largest study of South American natives to date in terms of sampled individuals and populations. A total of 1,011 individuals, representing 50 tribal populations from 81 settlements, were genotyped for up to 17 short tandem repeat (STR) markers and 16 single nucleotide polymorphisms (Y-SNPs), the latter resolving phylogenetic lineages Q and C. Virtually no structure became apparent for the extant Y-chromosomal genetic variation of South American males that could sensibly be related to their inter-tribal geographic and linguistic relationships. This continent-wide decoupling is consistent with a rapid peopling of the continent followed by long periods of isolation in small groups. Furthermore, for the first time, we identified a distinct geographical cluster of Y-SNP lineages C-M217 (C3*) in South America. Such haplotypes are virtually absent from North and Central America, but occur at high frequency in Asia. Together with the locally confined Y-STR autocorrelation observed in our study as a whole, the available data therefore suggest a late introduction of C3* into South America no more than 6,000 years ago, perhaps via coastal or trans-Pacific routes. Extensive simulations revealed that the observed lack of haplogroup C3* among extant North and Central American natives is only compatible with low levels of migration between the ancestor populations of C3* carriers and non-carriers. In summary, our data highlight the fact that a pronounced correlation between genetic and geographic/cultural structure can only be expected under very specific conditions, most of which are likely not to have been met by the ancestors of native South Americans.

## Author Summary

In the largest population genetic study of South Americans to date, we analyzed the Y-chromosomal makeup of more than 1,000 male natives. We found that the male-specific genetic variation of Native Americans lacks any clear structure that could sensibly be related to their geographic and/or linguistic relationships. This finding is consistent with a rapid initial peopling of South America, followed by long periods of isolation in small tribal groups. The observed continent-wide decoupling of geography, spoken language, and genetics contrasts strikingly with previous reports of such correlation from many parts of Europe and Asia. Moreover, we identified a cluster of Native American founding lineages of Y chromosomes, called C-M217 (C3*), within a restricted area of Ecuador in North-Western South America. The same haplogroup occurs at high frequency in Central, East, and North East Asia, but is virtually absent from North (except Alaska) and Central America. Possible scenarios for the introduction of C-M217 (C3*) into Ecuador may thus include a coastal or trans-Pacific route, an idea also supported by occasional archeological evidence and the recent coalescence of the C3* haplotypes, estimated from our data to have occurred some 6,000 years ago.

## Introduction

The way a certain habitat is first colonized by humans creates a primordial pattern of genetic variation that is subsequently attenuated by various demographic processes, including migration, population bottlenecks, fissions and fusions. A popular ramification of this paradigm is that most changes of the original genetic ‘make-up’ of a particular region follow trajectories established by geography and language [1] because, in addition to climatic conditions, the latter are the main conductors of gene flow. As a consequence, the type and degree of correlation observed between the genetic structure of an extant population on the one hand, and its linguistic and geographical structure on the other, should provide valuable information about the history of that population. Dissenting processes such as the adoption of a new language without substantial gene flow into the adopting population, for example, by ‘elite dominance’ are usually conceived as exceptions to the rule [2]. Following this line of arguments, any concordance between genetic, linguistic and geographic data should be indicative of steady settlement, isolation by distance and constant population growth whereas discordances suggest abrupt demographic changes such as major contractions or relocations [3].

A plethora of culture anthropologic and population genetic studies have corroborated the above viewpoint for various geographical regions, with Europe providing a most illustrative example. Thus, genetic markers of different time depths, including rapidly mutating short tandem repeats (STRs) and slowly mutating single nucleotide polymorphisms (SNPs), have been employed to trace the settlement of Europe over the last 40,000 years, from the Middle Paleolithic through the Neolithic into historical times [4]–[8]. Studies of Y-chromosomal markers, in particular, revealed a substantial correlation between genetic and geographic as well as linguistic patterns for parts of Europe and Asia that did not however become similarly apparent with mitochondrial or autosomal markers [5], [7], [9]–[13]. Hence, an analysis of molecular variance (AMOVA) of Y-STRs identified clearly distinguishable sub-clusters of western and eastern European Y chromosomes that were largely congruent with the Slavic and Romance language domains [7], [9], [11], and Y-chromosomal genetic discontinuities throughout the continent were found to coincide with linguistic boundaries [7], [14]–[16]. Other in-depth studies in Asia [17]–[20], Africa [21], [22], Melanesia [23]–[27] and globally [28], [29] also suggested that paternal (i.e. Y-chromosomal) but not maternal (i.e. mtDNA) lineage formation was strongly related to language dispersal [30]. These findings notwithstanding, it must be noted that at least some instances of male-mediated gene flow over major linguistic barriers have been inferred as well, for example, in Iberia [31] and in the Balkans [32]. Therefore, the correlation between genetics, language and geography may vary, particularly across a highly differentiated region such as Europe, depending upon the local effective population size and the time-depth of the DNA markers used.

South America was the last continent colonized permanently by modern humans. The popular “Out of Beringia” model [33] purports that, after the initial peopling of Beringia by North Asians >16,000 years ago, this proto-American population expanded, migrated southward along the coast and through an ice-free interior corridor, and differentiated either into a few large groups corresponding to the Eskimo-Aleut, Na-Dene and Amerindian linguistic families [34] or, in the view of many scholars, into hundreds of independent Paleo-Indian groups. On their way south, human populations may have experienced their first notable expansion in Central America [35]. However, the narrow Isthmus of Panama is a likely bottleneck that should have allowed only a comparatively small number of nomadic hunters, fishermen and gatherers to enter the northern Andean region via the Cauca and Magdalena rivers [36]. Another major population expansion is thought to have occurred in the Amazon basin from which the diverse habitats of the continent were then rapidly colonized [35]. As yet, this scenario could not be confirmed by any archeological evidence but is instead based mainly upon geographic reasoning as well as linguistic and molecular genetic data.

Today, most researchers agree that the initial human settlement of the Americas was a relatively swift process, a view spawned mainly by the dating of archeological findings from Paleo-Indian populations in North and South America. In particular, the Manis site in North America [37] has an estimated age of 13,860–13,765 calendar years before present (YBP) while the Monte Verde site in Chile dates back to an estimated 14,220–13,980 YBP [38]. Additional South American excavation sites in the north (Taima-Taima, Falcon, Venezuela), east (Lagoa Santa, Minas Gerais, Brazil), west (Pikimachay, Ayacucho Valley, Peru) and south (Los Toldos, Santa Cruz, Argentina) as well as in Amazonia (Pedra Pintada, Pará, Brazil) also indicate that human populations of high technological standard were scattered all over the continent by 12,000 YBP and were contemporary with the North American Clovis culture [39], [40]. As an alternative to terrestrial expansion, it has been suggested that some early settlers entered South America by sea [41]. Such a scenario would point towards trans-Pacific links with Polynesia [42] and the ancient Jōmon culture of Kyushu (Japan) [43], but no genetic evidence for a large-scale pre-Columbian immigration into the south other than via Central America has been found so far. In summary, it thus appears as if no consistent model for the peopling of South America has been established yet.

Greenberg's influential tripartite classification of the American language phyla [34], [44], in which the Amerind group reflects a single wave of colonization and ties together all extant native American languages, is no longer considered adequate [45]–[47]. Many historical linguists even doubt whether a credible relationship can be established at all between the long-diverged Amerind languages [48]. Moreover, Hunley and coworkers [49] highlighted an apparent incongruence between the American mtDNA pool and popular linguistic classifications. In general, molecular genetic data (i.e. blood groups, HLA, mtDNA, Y-chromosomal and autosomal STRs and SNPs) poorly fit the available linguistic, archeological and paleo-anthropological data from South America, and different types of genetic markers even gave contradictory results [50]. Undoubtedly, the hegemony of the pre-Columbian Inca empire in the 15^th^ Century and the contact with Europeans soon thereafter erased much of the previous relationship between genes, geography and culture through the elimination of some South American populations, and through the reduction of genetic diversity in others [39], [51].

Most population genetic studies so far have confirmed an Asian origin of all native American populations and provided clues as to the timing of the colonization and subsequent differentiation processes [52]–[54]. Reduced genetic diversity and strong differentiation prevail among South American natives, and do so more markedly in the east than in the west of the continent [50], [55], [56]. Strong genetic drift must have accompanied the dispersal process. However, while most researchers assume that, before the arrival of the Europeans, most communities in South America were rather small [50], [57], new evidence suggests that not only the Andes but also the Amazon region were home to large integrated populations between 1250 and 1600 AD, and that the small and isolated contemporary groups are only remnants of these [58].

Y-chromosomal studies of the peopling of South America are abundant because South American natives possess a founder Y chromosome defined by Y-SNP M3, a haplogroup commonly classified as Q1a3a [57], [59]–[61]. Some carriers of Q1a3a have also been found in Siberia, probably reflecting reverse gene flow from Alaska into Asia. On the other hand, sub-lineages downstream of Q1a3a and different members of upstream paragroup Q1a3* (defined by Y-SNP M346) have seldom been observed in South America [62]–[65]. In addition to haplogroup Q, only Asian haplogroup C is known also to occur in American natives where its presence is however restricted to sub-clades C3b and C3*. Lineage C3b, defined by Y-SNP P39, is apparently confined to North America [65]–[67]. In contrast, more ancient lineage C3* has only been detected unquestionably in four chromosomes from the northwest of South America as yet [68] and, more recently, also in a Tlingit individual from Southeast Alaska of self-reported indigenous ancestry [67]. No C haplogroups have been found in Central American natives as yet [66], [69].

In the present study, we examined whether the view that human genetic variation predominantly follows geographic and linguistic trajectories also holds for South America. To this end, we initiated the largest population genetic study of Y-chromosomal genetic variation in South American natives to date. We also report upon the discovery of a distinct cluster of C3* carriers in different albeit closely neighboring tribal and linguistic groups from Ecuador. At the same time as supporting our conclusion that the extant genetic structure of South American native populations, if any, is largely decoupled from the continent-wide linguistic and geographic relationships, this finding lends credit to the possibility of coastal or trans-Pacific migration that left no traces in North and Central America.

## Results

### Sample characteristics

**Figure 1:**
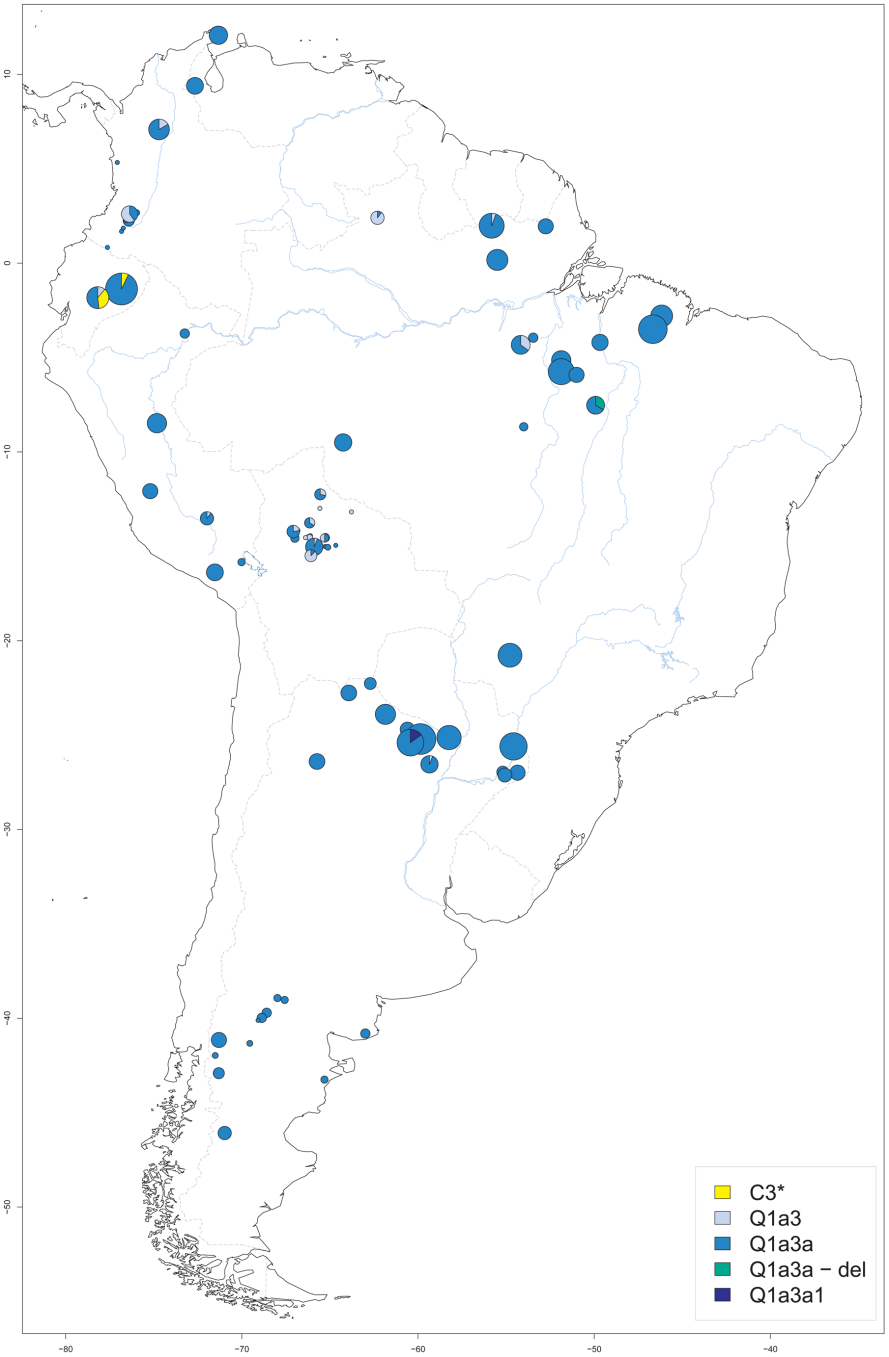
Origin of male native South American samples. For each sampling site, its geographic location as well as the size (proportional to the circle area) and Y-SNP haplogroup composition of the respective sample are shown. Blue lines: major aquatic systems; dashed gray lines: current national boundaries.

A total of 1011 DNA samples from native South American males, ascertained at 81 different sites, were available for analysis. The sample size per site ranged from 1 to 57, with a median of 8 and an interquartile range (IQR) of 2 to 17. Sampling sites were located between −78.18° and −46.17° longitude, and between −46.08° and 12.07° latitude. Places of origin ranged from the Guajira peninsula of Venezuela in the north and the Maranhão province of Brazil in the east to the Chubut province in Argentina in the south and the Pastaza province in Ecuador in the west (Figure 1). Additional information including the haplogroup, ethnic and linguistic assignment of each sample as well as site-specific measures of Y-STR haplotype variation is provided in Table S1.

Our samples included members of 50 different ethnic groups, with the number of males per group ranging from 1 for the Borjano, Embera, Movima, and Pastos to 90 (9% of the total) for the Toba (median number per group: 14.5, IQR: 6.3 to 26.5). Some 26 different language groups were present. Predominant groups included Mataco-Guaicuruan (n = 200; 20%), Tupi-Guaraní (n = 158; 16%) and Quechua speakers (n = 97; 10%). In contrast, Movima and Pano-Tacana were each spoken by a single individual only. Following Ruhlen [44], all samples were assigned to one of the four Amerindian language classes, namely Equatorial-Tucanoan (n = 399; 40%), Ge-Pano-Carib (n = 327; 32%), Andean (n = 166; 16%) and Chibchan-Paezan (n = 79; 8%), except for 40 Waorani males (4%) from two villages in the Pastaza province of Ecuador who spoke a Wao Tiriro isolate language. For the geographic distribution of language groups and classes, see Figure S1.

### Y-chromosomal SNP haplogroups

Five different Y-SNP haplogroups were present in the sample (Figure 1), with the large majority (n = 927; 92%) being Q-M3 (Q1a3a). This haplogroup was found throughout South America. Another 58 samples (6%) carried Q-M346 (Q1a3), a haplogroup previously reported from Argentina, Chile and Bolivia, and sporadically from North America [62] and Siberia [70]. Q1a3 carriers in our study originated from the north of South America and from Central Bolivia. The only sublineage of Q1a3a present in our study was Q-M19 (Q1a3a1), which was observed in six samples from the Argentinian Chaco province [71]. This sublineage has only been reported before from the Ticuna (Upper Amazon) and the Wayuu (Caribbean coast) [57], [72]. Another six samples from a single Gaviao village belonged to a Q1a3a-del haplogroup. This haplotype is characterized by a large deletion at the proximal azoospermia factor (AZFa) region that is responsible for the failure to amplify five of the STRs included in the YFiler kit, namely DYS389 I and II, DYS437, DYS439 and DYS635. However, other markers from distal AZFa could be analyzed in these samples, namely DYS438, M194, M242 and M199. Since both the *USP9Y* gene and the *DBY* gene are also located in the distal portion of AZFa [73], the deletion is not expected to affect fertility, which would be compatible with a spread of Q1a3a-del in South America. We did not observe other sublineages downstream of Q1a3a, such as Q-M194 (Q1a3a2) or Q-M199 (Q1a3a3) [65]. We did not test for the presence of recently published branch Q-SA01 (Q1a3a4) which had been detected before in Peru and Bolivia [64]. Notably, we found 14 individuals, all from Ecuadorian ethnic groups, who carried a C3*(xC3a-f) haplogroup. While three of these were Waorani and belonged to the Wao-Tiriro language isolate, the other 11 males belonged to the Kichwa speakers from the Pastaza province and lived close to the Waorani settlements. No carriers of C-P39 (C3b) were detected, a Y-SNP haplogroup that is apparently confined to North America [66], [74].

### Geographic distribution of Y-STR haplotype variation

We analyzed two sets of Y-STRs, namely a small set (DYS19, DYS389I, DYS389II, DYS390, DYS391, DYS392 and DYS393) and a large set (additionally including DYS437, DYS438, DYS439, DYS448, DYS456, DYS458, DYS635 and YGATAH4). Since both sets yielded similar results, we will only report upon the small marker set unless indicated otherwise. The haplotype diversity for the small marker set ranged from 0.0 to 1.0 per site, with a median of 0.88 and an IQR of 0.24 to 0.98, but these calculations were partially based upon small sample sizes and should therefore be considered with some caution. Bearing in mind the uneven and sometimes sparse distribution of sampling sites, we refrained from performing any geographical interpolation of haplotype diversity.

**Figure 2:**
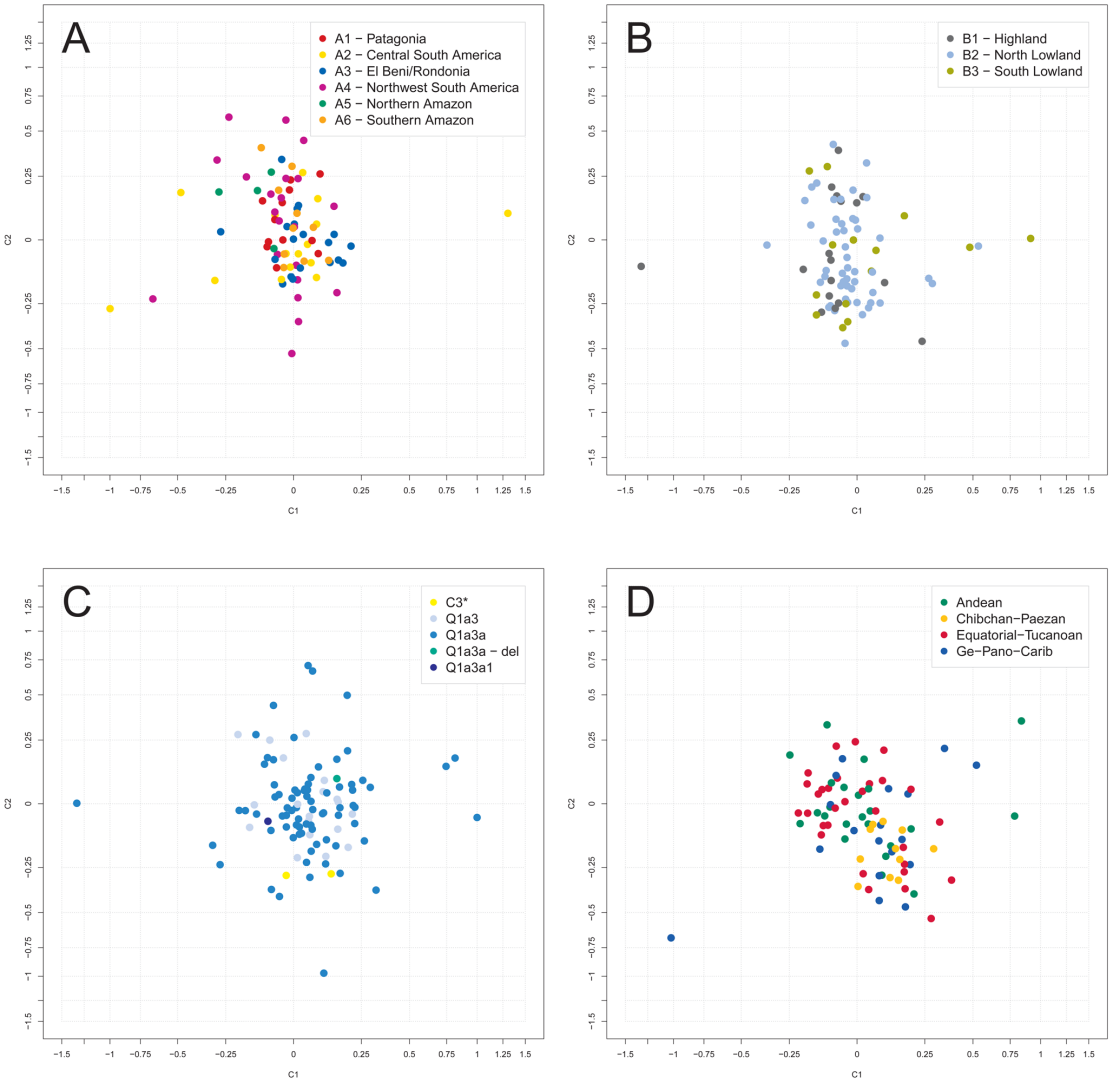
Multidimensional scaling (MDS) analysis of Y-STR genotypes. Depicted are the first two R_ST_-based MDS components, C1 and C2, as obtained for the small marker set. The center of each graph has been magnified for better resolution. A: grouping of sampling sites in AMOVA according to fine geographic clustering (A); B: AMOVA grouping according to broad geographic clustering (B); C: AMOVA grouping according to haplogroup affiliation; D: AMOVA grouping according to class of spoken language (excluding samples of individuals with unassigned language class).

In an analysis of molecular variance (AMOVA), variation between sampling sites was found to account for 21% of the total Y-STR genetic variation with the large marker set, and for 28% with the small marker set. These figures increased to 26% and 32%, respectively, when only Q1a3a carriers were analyzed. Next, sampling sites were combined into geographic clusters and analyzed jointly (Figure S2, Table S1). Clusters were defined manually taking altitude (high *versus* low), barriers (e.g. north *versus* south of the Amazon River) and distance between sites into account. We considered two types of geographic clusters. Fine clustering (A) assigned each sampling site to one of six clusters, namely A1 (‘Patagonia’; 6% of samples), A2 (‘Central South America’; 33%), A3 (‘El Beni/Rondonia’; 9%), A4 (‘Northwest South America’; 24%), A5 (‘Northern Amazon’; 8%) and A6 (‘Southern Amazon’; 20%). Coarse clustering (B) resulted in three clusters, namely B1 (‘Highland’; 12%), B2 (‘North Lowland’; 57%) and B3 (‘South Lowland’; 31%). Only a minor fraction of the total Y-STR variation of the small marker set was explained by differences between type A clusters (4% in a one-factor model and 1% in a two-factor model also including sampling site) or between type B clusters (3% and 1%, respectively). Virtually no systematic inter-cluster Y-STR variation was observed with the large marker set. Restricting the analysis to Q1a3a samples increased the above figures to 9% and 6% for type A clusters, respectively, and left them virtually unchanged for type B clusters. Multidimensional scaling (MDS) analysis of pair-wise R_ST_ between sampling sites also gave no indication of systematic population differences (Figure 2A and 2B). The first two MDS components (C1 and C2) explained 9.2% and 6.7% of the R_ST_-defined genetic variation, respectively, for type A clustering, and 8.4% and 7.0% for type B clustering, respectively.

Next, sampling sites were grouped for AMOVA and MDS according to their local Y-SNP haplogroups. The 18 sites harboring more than one Y-SNP haplogroup were split into homogeneous haplogroup-defined subgroups. As was to be expected, grouping by Y-SNP haplogroup explained a large proportion (∼85%) of the total Y-STR variation in the AMOVA. However, R_ST_-based MDS (Figure 2C) indicated only a modest separation between sites of C3*, Q1a3a1 and Q1a3a-del carriers, but not between sites of Q1a3 and Q1a3a carriers. The first two MDS components explained 6.8% and 5.3% of the R_ST_-defined genetic variation, respectively. One possible explanation for these apparently discrepant results may be that >90% of chromosomes belonged to haplogroup Q1a3a. Thus, the bulk of genetic variation occurred within one group and was therefore well explicable by group affiliation in an AMOVA but cannot, as a matter of principle, exhibit much structure in a sampling site-wise MDS.

To forestall concerns that the observed lack of genetic structure among native South American males was due to our sampling scheme, we performed an AMOVA of 100 random subsamples taken from the European portion of the Y Chromosome Haplotype Reference Database (YHRD), adopting the same number of sampling sites and the same distribution of sample size per site as in the South American data (see Materials and Methods). Over the 100 subsamples, geographic cluster affiliation (Figure S8) was found to account for 5.5% to 12.8% of the genetic variation in a two-factor analysis, with a median of 8.8% and an IQR of 8.0% to 9.6%. These figures were very similar to the proportion explained when all 214 European sampling sites were taken into consideration (mean: 8.9%, standard error: 0.2%; see Materials and Methods for details of the estimation procedure).

**Figure 3:**
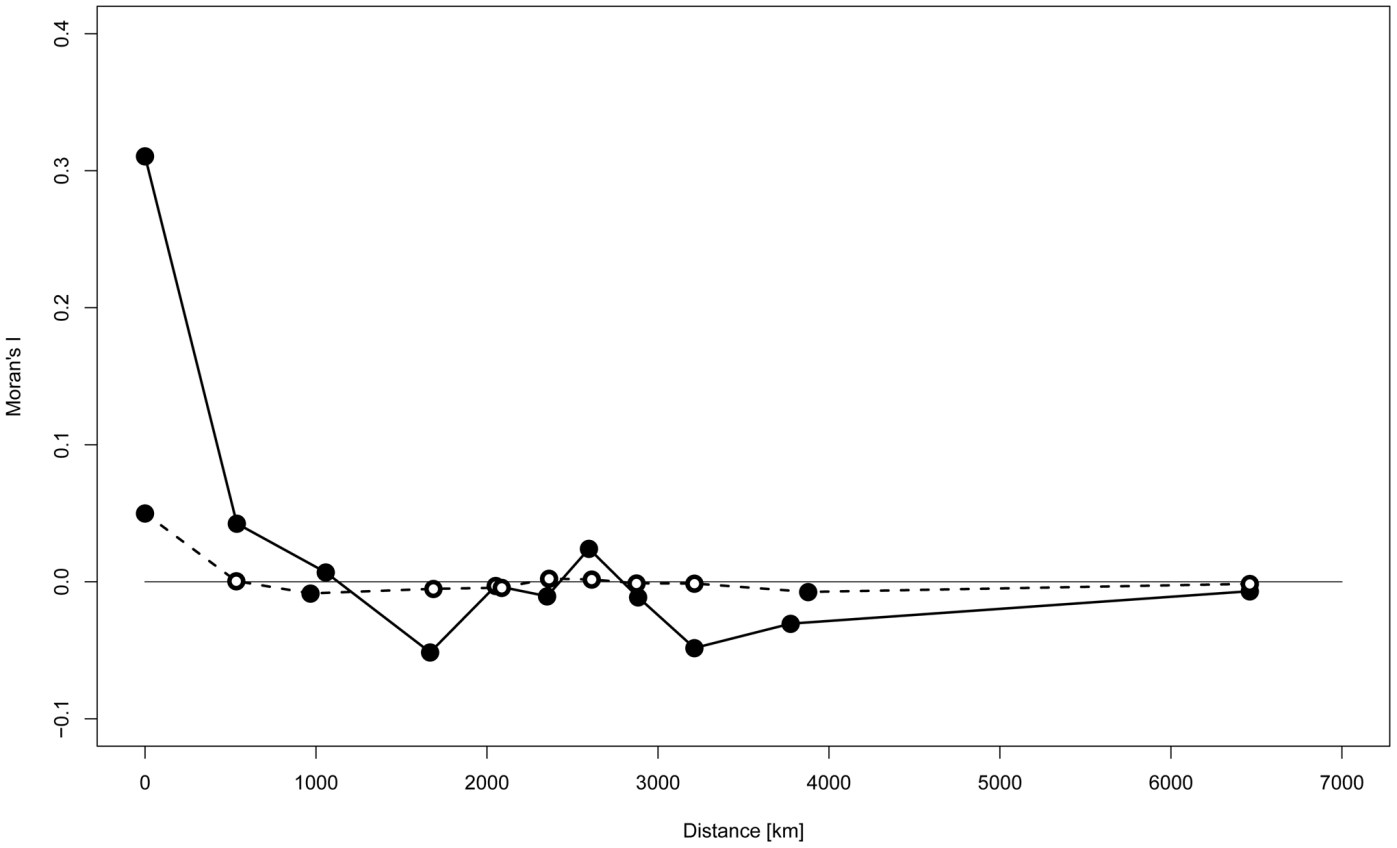
Spatial autocorrelation analysis of Y-STR genotypes. The spatial autocorrelation analysis was based upon the small marker set. Bold line: all samples; dashed line: Q-M3 (Q1a3a) haplogroup carriers only; filled circles: significant autocorrelation (p<0.05); empty circles: non-significant autocorrelation.

Spatial autocorrelation analysis (SAA) revealed a substantial positive correlation between Y-STR haplotypes only for sites that were at most a few hundred kilometers apart (Figure 3). With the exception of A1 cluster ‘Patagonia’, SAA confined to geographically defined clusters revealed a similar pattern, most convincingly for clusters B2 ‘North Lowland’, A6 ‘Southern Amazon’ and A4 ‘Northwest South America’ (Figure S3). When the SAA was confined to Q1a3a carriers, however, no significant autocorrelation of Y-STR haplotypes was observed apart from a modest correlation within sites, i.e. at zero distance (Figure 3).

### Y-STR network analysis

After removing samples that carried at least one deletion or duplication, median-joining network analyses were performed for chromosomes with haplogroup Q or C3*. The resulting haplogroup Q network was characterized by star-like offshoots and little structure. No correlation was observed between any of the linguistic or geographic classifications and Y-STR haplotypes belonging to haplogroup Q (Figures S4, S5, S5, S7). The C3* network structure will be alluded to in more detail below.

### Haplotype variation and language

We also assessed the degree to which Y-STR variation correlated with spoken language. To this end, we considered two types of linguistic categories, namely the 26 narrowly defined language groups and the four more broadly defined language classes of Ruhlen [44] (Andean, Chibchan-Paezan, Equatorial-Tucanoan and Ge-Pano-Carib) plus a language isolate for 40 Waorani samples (Table S1). A highly significant association was observed between haplogroup and both language class (Cramer's V = 0.20, p<10^−8^, Table 1) and language group (V = 0.41, p<10^−8^, Table S3). In view of their uncertain linguistic relationship with the remaining samples, the Waorani haplotypes were excluded from subsequent AMOVA and MDS analysis. Sampling sites comprising more than one language group (n = 7) or class (n = 2) were split into homogeneous subgroups. Language class was not found in an AMOVA to explain much of the Y-STR genetic variation (<0.5% for both marker sets). In contrast, differences between the more narrowly defined language groups explained 12% and 8% of the variation for the large and the small marker set, respectively. MDS analysis did not indicate any strong genetic differences between language classes, except for a weak clustering of sites belonging to the Chibchan-Paezan class (Figure 2D). The first two MDS components explained 8.0% and 6.6% of the R_ST_-defined genetic variation.

**Table 1:** Correlation between Y-SNP haplogroup and language class.

Language class	Y-SNP Haplogroup				
	C3*	Q1a3	Q1a3a	Q1a3a - del	Q1a3a1
Andean	11	5	150	0	0
Chibchan-Paezan	0	22	57	0	0
Equatorial-Tucanoan	0	12	387	0	0
Ge-Pano-Carib	0	19	296	6	6
Wao-Tiriro isolate	3	0	37	0	0

### Occurrence of C3* in South America

**Figure 4:**
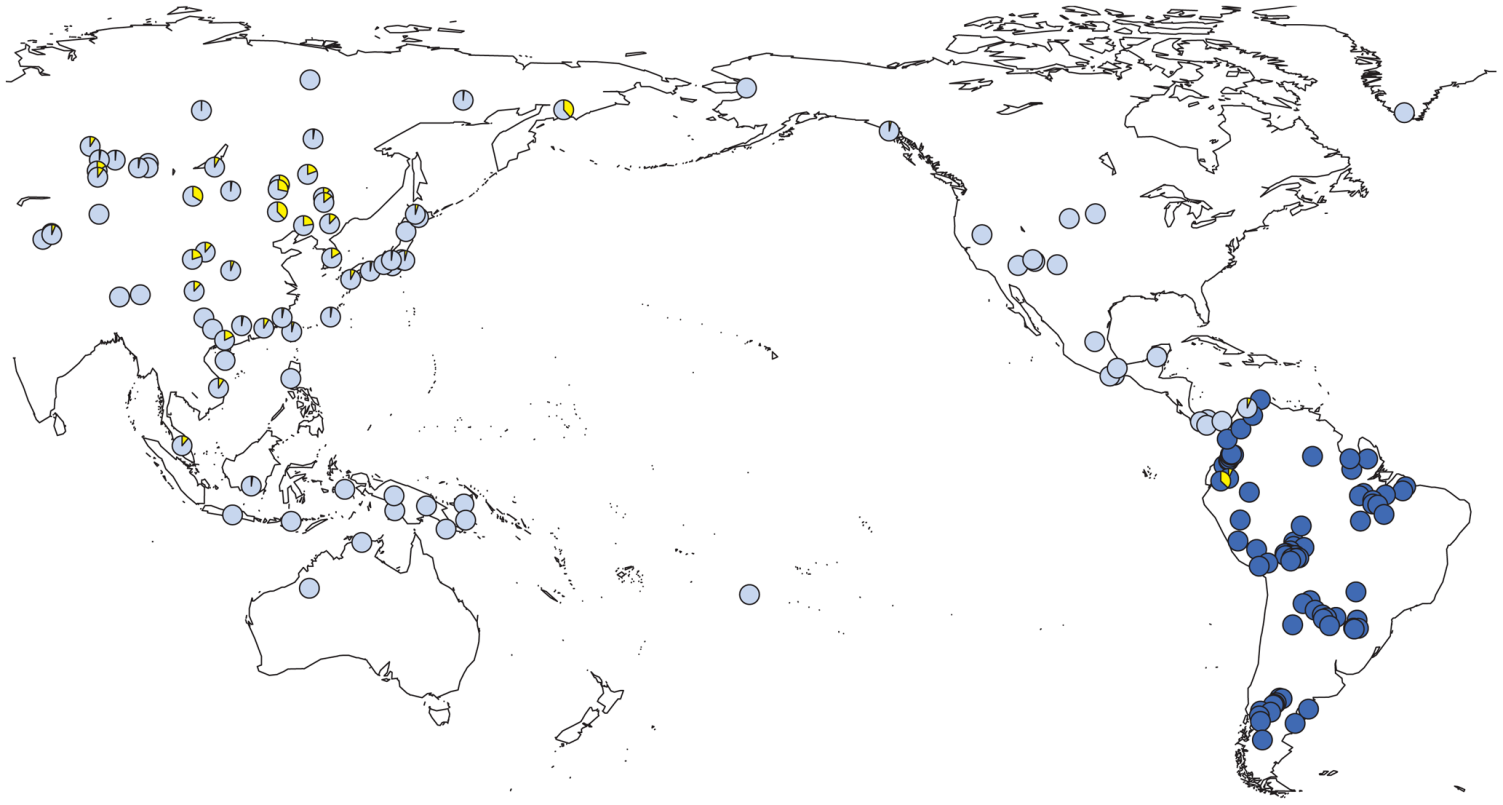
Prevalence of Y-SNP haplogroup C-M217 (C3*) around the Pacific Ocean. Light blue: previous studies; dark blue: present study; yellow: relative frequency of C-M217 (C3*) carriers.

**Figure 5:**
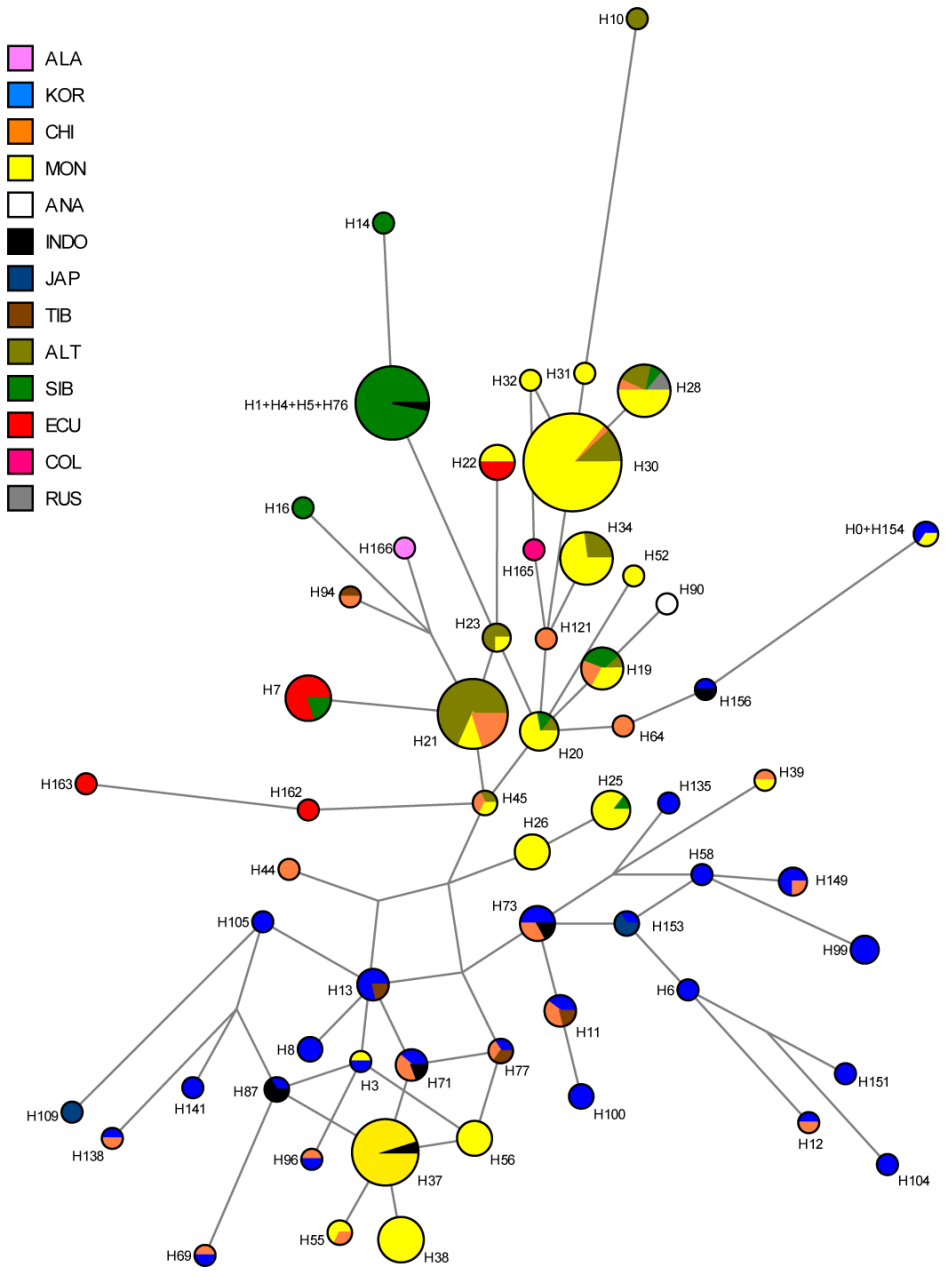
Median-joining network of 167 different Asian and American Y-STR haplotypes carrying Y-SNP haplogroup C3* (from this and previously published studies). The median-joining network is based upon markers DYS19, DYS389I, DYS389II-DYS389I, DYS390, DYS391, DYS392, DYS393 and DYS439 (see Materials and Methods for details). ALA: Alaskan; KOR: Korean; CHI: Chinese, including Daur, Uygur, Manchu; MON: Mongolian, including Kalmyk, Tuva, Buryat; ANA: Anatolian; INDO: Vietnamese, Thai, Malaysian, Indonesian, Philippines; JAP: Japanese; TIB: Tibetan, Nepalese; ALT: Altaian, including Kazakh, Uzbek; SIB: Teleut, Khamnigan, Evenk, Koryak; ECU: Ecuadorian, including Waorani, Lowland Kichwa, COL: Colombia, including Wayuu; RUS: Russian.

We observed a considerable number of C3* haplogroup carriers in our study (n = 14). These were confined to the northwest where C3* was found at substantial frequency in two culturally very distinct native groups from Ecuador, namely the Kichwa (26%) and the Waorani (7.5%). The C3* haplogroup was absent from all other samples. Previously published data [20], [25], [66], [67], [70], [75]–[77] indicate that C3* occurs at a high frequency throughout continental East Asia (Figure 4) and is most prevalent in Kamchatka (38% in Koryaks) and in Outer and Inner Mongolia (36% and 38%, respectively). At the Pacific coast, the average C3* frequency is higher in Korea (10%) than in Japan (3%), with the notable exception of 15% for the Ainu from Hokkaido, representing the aboriginal people of Japan. In striking contrast, this haplogroup is apparently absent from the whole of North and Central America, with the exception of a single C3* carrier of self-reported indigenous ancestry from Southeast Alaska [67], as well as from Melanesia east of Borneo and Polynesia. We performed a median-joining network analysis of the Y-STR haplotypes of the 14 C3* carriers in our study and of 396 carriers identified in previous reports [66], [67], [70], [78]–[83]. In the resulting network (Figure 5), native South American C3* carriers from the present study (marked in red in Figure 5) belonged to separate and rather distant clusters at the periphery of the network, suggesting that the time of the last contact between these two groups predated the time of the initial colonization of the Americas. The Alaskan Tlingit C3* haplotype H166 (marked in pink) is between four and five steps away from the Ecuadorians, but is connected to the same frequent haplotype, H21 via a quasi-median. The most frequent Ecuadorian C3* chromosome H7 (occurring eight times in the Kichwa) shared an identical 8-locus haplotype with two Koryak samples from Kamchatka. The other three Kichwa haplotypes were related to this prevalent type by a one-step mutation at DYS391 (H162; occurring twice) and by two steps at DYS391 and DYS439 (H163; occurring once). This cluster was connected to the core of the network via a quasi-median, thereby highlighting its substantial distance to common Asian types. The three identical Waorani haplotypes differed from three identical Mongolians by a single step mutation only and grouped together with these in haplotype H22 for the small marker set plus DYS439. The putative C3* haplotypes of the Colombian Wayuu [66] were only distantly related to the Ecuadorians (H165).

### Time to the most recent common ancestor (TMRCA) of C3* chromosomes

The median TMRCA estimate for the haplogroup C3* chromosomes in our data was between 168 and 206 generations (1^st^ quartile: 113 to 135 generations; 3^rd^ quartile: 252 to 317 generations), depending upon the employed population growth model and prior population size. Assuming a generation time of 30 years, this would imply an MRCA for the 14 C3* chromosomes at 5040 to 6180 YBP. Similarly, the posterior mean of population parameters N_c_ and N_a_ fell short of their prior counterparts by a factor of 5 to 25, judged by the respective median (Table 2). Inclusion of the single Tlingit C3* chromosome from Alaska [67] increased the TMRCA estimate to a median between 202 and 249 generations (1^st^ quartile: 140 to 168 generations; 3^rd^ quartile: 294 to 734 generations). The model-specific change in median TMRCA estimate ranged from 34 to 43, corresponding to a time difference of 1020 to 1290 years.

**Table 2:** Estimation of the TMRCA (in generations) of C3* Y chromosomes.

	BATWING model 0		BATWING model 2	
Prior N_c_ (N_a_)	380	2000	380	2000
Haplogroup C3* chromosomes from present study				
**TMRCA**	192 (127–292)	206 (135–317)	168 (113–252)	176 (117–265)
**Posterior N_c_ (N_a_)**	98 (64–118)	108 (70–168)	72 (49–108)	77 (51–117)
Inclusion of a previously published C3* chromosome from Alaska [67]				
**TMRCA**	231 (158–341)	249 (168–374)	202 (140–294)	214 (147–316)
**Posterior N_c_ (N_a_)**	140 (95–163)	156 (104–234)	99 (69–145)	108 (73–160)

Prior N_c_ (N_a_): mean of the prior distribution adopted for randomly sampling the two population size parameters in BATWING (N_c_ for model 0, N_a_ for model 2). The results were obtained from 2 million BATWING runs per combination of haplogroup, distribution parameter and population model. Given are the median and, in parentheses, the inter-quartile range of the TMRCA (in generations) and of the posterior mean of N_c_ and N_a_, each time taken over the last 1 million runs. The first million runs were used for burn-in.

### Historic migration of C3* carriers

If haplogroups Q and C3* both entered the American continent from Asia at the same time 15,000 YBP, then C3* would have been expected to be more widespread than has been reported so far. We employed three simplified models of population divergence (see Materials and Methods for details) to ascertain which migration rates likely prevailed between the subpopulations preceding C3* carriers (designated SA/C+) and non-carriers (SA/C− and NA/C−, see Figures S10, S11, S12). When considering South America alone (scenario SA), the median of the migration rate into population SA/C− was 0.023 (inter-quartile range 0.001–0.036), while that into SA/C+ was 0.092 (0.072–0.147). Similar results were obtained when South American and North American non-carriers were collapsed into one population (scenario AA), with a median migration rate into SA/C− and NA/C− of 0.027 (0.010–0.065), and into SA/C+ of 0.112 (0.073–0.154). Assuming a ten-fold larger effective population size, albeit in a smaller number of simulations, (see Figures S13 and S14) led to very similar results, with a median migration rate into SA/C− (scenario SA-10x) of 0.034 (0.024–0.045) and into SA/C− and NA/C− (scenario AA-10x) of 0.011 (0.004–0.018). Consideration of three populations (scenario BA) yielded median migration rates from the other two populations combined of 0.079 (0.075–0.121) into SA/C−, 0.058 (0.019–0.107) into NA/C−, and 0.140 (0.095–0.157) into SA/C+. The median migration rate into the common ancestral population of SA/C− and NA/C− was 0.117 (0.067–0.164). It should be remembered, however, that all migration rates into one of the non-carrier ancestral populations included migration from the other non-carriers, which explains why these rates are substantially higher than with the two-population models.

## Discussion

In the largest population genetic study of native South Americans to date, we investigated the continent-wide pattern of Y-chromosomal variation exhibited by both short tandem repeats (Y-STRs) and single nucleotide polymorphisms (Y-SNPs). The analyzed sample comprised 1011 males from 50 different ethnic groups, each belonging to one of 26 different language groups. The 81 sampling sites were chosen so as to represent the main habitats in South America, i.e. the Andes, the Caribbean Coast, the Amazon region, the Central Plateau, the Gran Chaco and Patagonia. Since our main interest was to infer if and how the extant pattern of genetic variation may reflect the demographic history of the continent, we deliberately excluded samples with Y-SNP haplotypes of likely European or African origin.

### 

#### Decoupling

Our study revealed that the extant Y-chromosomal genetic variation among native South America males is lacking any clear structure that could sensibly be related to their continent-wide geographic and linguistic relationships. Notable genetic similarity was only observed over a few hundred kilometers, and only (narrowly defined) language group, but not (broadly defined) language class, was found to explain some of the Y-chromosomal genetic variation observed. Such decoupling contrasts with the situation on other continents, particularly Europe and Asia, where many instances of a strong correlation between genetics on the one hand, and geography and spoken language on the other, have been reported. Random resampling from a pool of European Y chromosomes adopting the same number of sampling sites and the same distribution of the sampling size per site as in the present study yielded consistently higher levels of Y-STR genetic variation explicable by geographic structure. Therefore, the observed decoupling of Y-chromosomal genetic variation from geography in native South America males is unlikely to be due to the particular sampling scheme of our study. Despite a few regional examples of decoupling as, for example, in the Caucasus [84], “parallel linguistic and allele-frequency change” is generally considered to be a rule [85] to which pre-Columbian South America would thus be an exception at continental scale.

Our observations highlight the fact that a correlation between genetic and geographic/cultural structure can be expected only under very specific conditions. In particular, Europe and Asia have been characterized by long periods of human habitation, with major movements occurring in due consideration of geographic and cultural barriers. In contrast, pre-Columbian South America is likely to have experienced a very different demographic history, with a comparatively recent arrival of the first humans, followed by very rapid dispersal over long distances, subsequent periods of extended geographic isolation of small groups, and substantial increases in population density only comparatively shortly before the European conquest. In other words, colonization may have been too fast relative to the subsequent time of habitation, and population expansion too recent, to establish and/or maintain a considerably structured pattern of Y-chromosomal genetic variation.

The above view is supported by different characteristics of our Y-STR data: a locally confined autocorrelation of repeat size, little geographic differentiation of the gene pool, limited correlation between language group (but not class) and genetic variation, and the emergence of clumpy star-like haplotype networks. Localized genetic autocorrelation is consistent with a localized isolation-by-distance model. In other words, our data suggest that the spread of male lineages among native South Americans, if any, was geographically confined. Since the autocorrelation of repeat size is virtually lost when only Q1a3a carriers are considered, the little structure that is there likely reflects the migration of other Y-SNP haplogroups. The accrual of star-like Y-STR networks for Q haplotypes further supports the view of an autochthonous evolution of small populations (Figures S4, S5, S6, S7). It is therefore likely that the Q1a3a carriers who entered into South America were already quite heterogeneous in terms of their Y-STR haplotypes. Finally, broad language class according to Greenberg [34] and Ruhlen [44] correlated poorly with Y-chromosomal genetic similarity as well, although it cannot be excluded that this is partially due to an inadequacy of the classification *per se* [45]–[47].

Our study was confined to Y-chromosomal genetic variation, which implies that the observed decoupling of genetics from geography and language, and the conclusions drawn from it, may apply only to the male lineages of native South Americans. If the cultural characteristics of these people and their ancestors were such that a different demography ensued for males and females, for example, by stronger patrilocality than matrilocality, then our results cannot be generalized so as to embrace the whole genealogy. However, previous studies at a smaller scale than ours revealed a striking similarity between the levels of differentiation of the South American native Y-chromosomal and mitochondrial DNA pool [86]. Moreover, the observed decoupling of genes and geography/language is also consistent with previous reports based upon autosomal STRs [56], [87]. Therefore, it may be concluded that a model of rapid colonization and subsequent long-term isolation of small groups applied not only to the male lineages in pre-Columbian South America, but to the population as a whole.

### Presence of Y-SNP haplogroup C-M217 (C3*) in South America

The presence of Y-SNP haplogroup C-M217 (C3*) in the northwest of South America, and its concomitant absence from most of North and Central America, are intriguing in view of the high prevalence of this haplogroup in Central, East and Northeast Asia. Given the large population size of native North Americans, it appears unlikely that the early settlers of America carried C3* with them, and that the haplogroup got lost by genetic drift in the north, but not in the south. In fact, the locally confined occurrence of C3* in South America would require migration rates into the ancestral C3* and Q carrier populations that are so low (most likely only 2.5% out of the C3* carrier population) that they are hardly compatible with a long period of joint immigration from Asia. Instead, an independent introduction of C3* into South America appears plausible not the least because it would be consistent with the observed pattern of locally confined Y-STR autocorrelation as well. This view is further supported by the comparatively recent coalescence of the 14 C3* haplotypes from the present study, which appears to have occurred some 200 generations ago, corresponding to 6000 years. The above notwithstanding, inclusion of an isolated Tlingit C3* haplotype found in Alaska prolonged the coalescence time estimate by no more that approximately 40 generations which means that a North American origin of the Ecuadorian C3* haplotypes, albeit less likely *prima facie*, cannot be ruled out.

In view of the above, two scenarios for the introduction of C3* into Ecuador seem credible: (i) one or more late migratory waves that quickly passed North and Central America without leaving a trace of C3*, and (ii) long-distance contact with East Asia. As regards the second scenario, there appears to be at least some archaeological evidence for a pre-Columbian contact between East Asia and South America [43]. In particular, the similarity of ceramic artifacts found in both regions led to the hypothesis of a trans-Pacific connection between the middle Jōmon culture of Kyushu (Japan) and the littoral Valdivia culture in Ecuador at 4400–3300 BC. In view of the close proximity of the spotty C3* cluster to the Valdivia site, which was considered at the time to represent the earliest pottery in the New World [40], it may well be that C3* was introduced into the northwest of South America from East Asia by sea, either along the American west coast or across the Pacific (with some help by major currents). The considerable differences between the extant Y-STR haplotypes of Ecuadorian and Asian C3* carriers would clearly be explicable in terms of their long divergence time. The differences between C3* chromosomes carried by different ethnic groups in Ecuador, on the other hand, highlight that population splits followed by limited gene flow are characteristic of the genetic structure of South American natives [88].

Of the 14 C3* haplotypes observed, 11 belonged to Lowland Kichwa today living in geographic proximity or even in the same villages as the Waorani of which three men from different families carried identical C3* haplotypes. It is important to realize that the Waorani, who were known for their extreme ferocity against invaders, were the only human inhabitants of a region of approximately 20,000 km^2^ east of the Andes between the Napo and Curaray rivers before their first peaceful contact with outsiders in 1958 [89]. A post-contact introduction of the C3* haplogroup from a Kichwa ancestor to the Waorani families can be excluded according to the genealogical record. The difference of 10 to 16 mutational steps between the Waorani haplotype and the Kichwa cluster comprising four more closely related haplotypes (see Table S5) corroborates our view that even geographically neighboring ethnic groups survived for a long time in isolation from each other.

### Conclusion

In summary, our study revealed that the Y-chromosomal genetic variation of South American natives lacks a clear structure that could be related to the continent-wide geographic and linguistic relationship, suggesting a history of rapid peopling and subsequent evolution in small groups. Moreover, it appears unlikely that the South American natives are descendants of a single terrestrial wave of migration. Instead of being confined to a major founding lineage of the Q branch of the human phylogeny, as has been widely held to be the case in the past [60], the continent hosts other Asian haplogroups as well (e.g. those belonging to the basal C3* clade). Further characterization of their distribution is likely to provide new insights into the demographic history of South America.

## Materials and Methods

### DNA samples and linguistic classification

The present study complies with the ethical principles of the 2000 Helsinki Declaration of the World Medical Association. The current study was approved by the institutional review board of the Institute of Legal Medicine and Forensic Sciences Berlin under protocol authorization number 11-2010/02. Buccal swabs, liquid saliva and capillary blood spotted on FTA cards were obtained from 1452 South American males. Of these, 441 individuals carried Y-SNP haplogroups other than Q or C (including clades B, E, G, I, J, R, and T as well as subgroups within these clades). Such samples were considered as being of post-Columbian European or African origin and were excluded from further analysis. Typical East Asian or Oceanian lineages other than C (e.g. D, O or M) were not detected. Only a single Y chromosome with haplogroup R1a, common in many parts of Eurasia, was observed and was excluded from further analysis because of the impossibility to distinguish a pre- from a post-Columbian origin. The remaining 1011 males were deemed of native American ancestry adopting the widely accepted view that all Paleoindian ancestors originated from East Asia [90]. These individuals were ascertained at 81 sampling sites in seven South American states, namely Argentina, Bolivia, Brazil, Colombia, Ecuador, Peru and Venezuela (Figure 1). Samples were gathered by the authors and their collaborators for research purposes only. All samples were fully anonymized. Written informed consent was obtained from all participants prior to the study. Data collected in the field included tribal affiliation, population size, geographic location of the home settlement, language spoken and patrilineal relationships. The latter information was used to exclude likely close relatives and individuals of mixed ancestry. Linguistic group classifications were based upon Campbell's scheme [46] whereas language classes (Andean, Chibchan-Paezan, Equatorial-Tucanoan, Ge-Pano-Carib) were assigned following Ruhlen [44] (see Table S1).

### DNA typing

All wet lab analyses were performed at the authors' institutions in Porto (Portugal), Buenos Aires (Argentina), Belém (Brazil) and Berlin (Germany). DNA was extracted following standard protocols using either Chelex or phenol-chloroform-based methods or magnetic bead extraction with QIAsymphony (Qiagen, GmbH Hilden, Germany). A total of 919 samples (91%) were typed for the 17 Y-STRs of the AmpFlSTR Yfiler kit, following the manufacturer's instructions (AmpFlSTR Yfiler PCR Amplification kit, Applied Biosystems, Foster City, USA). Although duplicated marker DYS385ab was typed in the whole sample, the respective genotypes were disregarded in all subsequent analyses due to ambiguous allele assignment. The remaining 15 markers will henceforth be referred to as the 'large marker set' (DYS19, DYS389I/II, DYS390, DYS391, DYS392, DYS393, DYS437, DYS438, DYS439, DYS448, DYS456, DYS458, DYS635 and YGATA H4). Complete genotype information on these markers was available for 874 samples (86% of the total). Another 92 older samples (9%) could not be retyped for the large marker set for logistic reasons. They had been characterized before in Buenos Aires and Belém for the so-called ‘minimal haplotype’, either by two multiplex PCRs [91] or according to protocols published by Palha and coworkers [92], [93]. The corresponding seven markers (DYS19, DYS389I/II, DYS390, DYS391, DYS392 and DYS393) will henceforth be referred to as the ‘small marker set’ here. PCR product separation and detection was carried out on different ABI genetic analyzers (Applied Biosystems, Foster City, USA) including ABI310, ABI3100 and ABI3130 machines. Alleles were identified by means of the reference ladder provided with each kit and following recommendations by the DNA Commission of the International Society of Forensic Genetics (ISFG) [94]. In the present study, allele designations for DYS389I and DYS389II refer to the repeat numbers at individual loci rather than the repeat numbers revealed by the multiplex genotyping method. Comprehensive marker genotype information is provided in Table S5.

All samples were subjected to a first round of Y-SNP analysis using mainly the ABI PRISM *SNaPshot* system (Applied Biosystems, Foster City, USA) to genotype a panel of phylogenetic markers that define the most frequent haplogroups in South America (M42, M207, M242, M168, M3, M145, M174, M213, RPS4Y711, M45, P170 and M9) [68], [95]. Those 441 samples belonging to Y-SNP haplogroups other than Q and C were excluded from further analysis (see above). In the Porto laboratory, all remaining samples were typed by the *SNaPshot* multiplex reaction (‘multiplex Q’) that allows genotyping of Y-SNPs M242, P36.2, M346, M3, M19, M194 and M199. In Berlin, a similar *SNaPshot* multiplex (‘SA SpecQ’) was used to subtype haplogroup Q for Y-SNPs M19, M194, P292, M3 and M199 [68]. Since haplogroup C occurs in both Asia and North America (in the form of C3b*, defined by Y-SNP P39), we assumed that some C lineages were autochthonous as well. Therefore, another multiplex (‘SA SpecC’) was established in Berlin to allow further subtyping of haplogroup C (mainly as subclade C3*, defined by Y-SNP M217). The full *SNaPshot* panel includes M407, M48, P53.1, M217, P62, RPS4Y711, M93, M86 and P39. Instead of the *SNaPshot* assay, the Buenos Aires institute used real time PCR followed by High Resolution Melting Analysis for Y-SNP genotyping [96]. The Y-SNP haplogroup nomenclature used here follows the recommendations by the Y Chromosome Consortium [65]. The data were checked for invalid alleles, duplicates and missing genotypes using in-house scripts for the R software v2.14.0 [97]. See Figure S9 for an illustration of the phylogenies of haplogroups C3* and Q*.

All genotypes generated in the present study are provided in Table S5 and were logged in the online Y Chromosome Haplotype Reference Database (YHRD), maintained by the Institute of Legal Medicine and Forensic Sciences, Charité - University Medicine, Berlin, Germany (www.yhrd.org).

### Reference samples

For some analyses, we also drew upon external data resources. Thus, information on the C3* haplogroup frequency in Asia and the Americas was obtained from 6,562 haplotypes reported in 109 different population genetic studies [20], [24], [66], [69], [70], [75]–[77]. In fact, 316 of these Y chromosomes (4.8%) were reported to carry haplogroup C3*. The corresponding Y-STR haplotypes were also extracted from the literature as well as from YHRD data submissions [66], [70], [78]–[83] to allow comparative network analyses jointly with our own data (see Table S6). For network analyses and estimation of the time to the most recent common ancestor (see below), we included the genotype data of a recently published C3* haplotype from Southeast Alaska with self-reported indigenous ancestry [67] (sample #2 from Table S8 of the original publication).

### Statistical analysis

Allele and haplotype frequencies were estimated by counting. Gene diversity and haplotype diversity were calculated following Nei [98], [99]. The R software v2.14.0 [97] was used for statistical analysis unless indicated otherwise, and to create graphs. The association between haplogroup and language group or class was measured by Cramér's contingency coefficient V [100] and tested for statistical significance using Fisher's exact test with 100 million simulations, as implemented in the *fisher.test* function of R.

#### Analysis of molecular variance (AMOVA)

Genetic relationships between different groups of males were quantified by means of R_ST_, thereby taking the evolutionary distance between individual Y-STR haplotypes into account [101], [102]. Groups were defined according to sampling site, linguistic group or class affiliation, geographic origin or Y-SNP haplogroup, respectively. We employed Arlequin v 3.5.1.2 [103] to estimate R_ST_ and for significance testing of R_ST_>0 using randomization (1000 replicates per comparison). Samples carrying a deletion at one or more markers were excluded from the analysis of the respective marker set.

In order to assess if and how the occasionally small sample size per site in our study may have affected our scientific conclusions, we performed a series of AMOVAs on European samples that were typed for the small marker set and for which haplotypes were logged in YHRD. A total of 27,585 haplotypes from 214 sites were available for analysis (release 39 from February 17, 2012). In order to take recent migration into account, we deliberately excluded metropolises with more than 500,000 residents (see Table S4). The sampling sites were then assigned to one of six geographic regions (see Figure S8) broadly defined according to a previous Y-STR-based classification [7]. We then generated 100 random subsets featuring the same number of sampling sites and the same distribution of the sample size per site as our South American data. An AMOVA was then performed for each sub-sample. Analysis of the complete European data set turned out unfeasible with Arlequin due to computer memory limitations. Instead, we generated 100 random subsamples of the full data set each time limiting the sample size per site by 50, and the AMOVA results were then averaged over replicates.

#### Spatial autocorrelation analysis

Spatial autocorrelation analysis of haplotypes, treating the repeat number of each Y-STR as the primary observation, was performed by way of Moran's I [104] using an in-house program. Moran's I measures the correlation between the Y-STR repeat numbers of haplotypes sampled at a given geographical distance. Pair-wise great circle distances (GCD) between sites were determined from Cartesian coordinates as obtained from the 2004 version of the CIA World Factbook and other publicly available sources. Autocorrelation was evaluated over 11 distance classes, namely one class for haplotypes sampled at the same site (i.e. GCD equal to zero) and ten additional classes with equal numbers of observations, covering the whole range of non-zero inter-site distances in our data. The statistical significance of Moran's I was assessed by means of a permutation test reshuffling samples over locations in 10,000 replicates.

#### Multidimensional scaling analysis

Multidimensional scaling (MDS) analysis was based upon pair-wise R_ST_ between sampling sites as estimated with Arlequin (see above), and was carried out with the *cmdscale* function of R v2.14.0 [97]. Plots of the first two MDS components, C1 and C2, capturing the most and second most R_ST_-defined variation, were generated with R using in-house scripts.

#### Median-joining network analysis

Median-joining Y-STR networks [105] were calculated for C3* and Q haplotypes with NETWORK v4.6.1.0, and edited using NETWORK Publisher v1.3.0.0 (Fluxus Technology Ltd). The analyses were based upon the small marker set for Q chromosomes (n = 977, excluding deletions and duplications) and the small marker set plus DYS439 for 167 South American and Asian reference C3* chromosomes, respectively. In the network construction, each marker was weighted by the inverse of its marker-specific mutation rate (see Table S2).

To investigate further the genetic relationship between the 14 Amerindian C3* haplogroup carriers from Ecuador detected in our study and those reported from Asia, Alaska and Colombia (the latter without full typing of markers downstream of M-217), we drew upon previously published Y-STR data on 396 carriers of C3*(xC3a-f). In particular, we included 154 samples from Zegura et al. [66], five from Cinnioglu et al. [79], one from Chang et al. [78], two from Gayden et al. [80], 62 from Kim S.H. et al. [81], 90 from Malyarchuk et al. [70], 56 from Kim Y.J. et al. [82], 25 from Kwak et al. [83] and a single chromosome (sample #2) from Schurr et al. [67]. The network analysis was based upon markers DYS19, DYS389I, DYS389II-DYS389I, DYS390, DYS391, DYS392, DYS393 and DYS439; no genotypes were missing in the analyzed samples. Markers were again weighted by the inverse of their specific mutation rate. We applied the star-contraction, median-joining and maximum-parsimony options of the NETWORK software in our analyses.

#### Time to the most recent common ancestor (TMRCA)

We used BATWING [106] for estimating the TMRCA for all C3* haplotypes from the present study. In a second analysis, we additionally included a recently published C3* haplotype of self-reported indigenous ancestry (sample #2) from Southeast Alaska [67]. The same marker set was used for TMRCA estimation as employed in the network analyses (see above). We ran BATWING under different assumptions, each time excluding migration and adopting either a constant population size N_c_ (model 0 in BATWING) or an ancestral population size N_a_, followed by exponential growth after some scaled time point β before present (model 2). Both the population size and the marker-specific mutation rates were sampled from appropriate Γ distributions. The mean population size N_c_ in model 0 and the ancestral population size N_a_ in model 2 were set to either 380, 1000, 1200 or 2000, following previous suggestions [29]. For model 2, we assumed a Γ(1,200) prior on the population growth rate α (corresponding to an expected rate of 0.005) and a Γ(2,1) prior on β (corresponding to an expected scaled time of 2 units). For each model and parameter combination, BATWING was run two million times, with the first one million runs treated as a burn-in that was not included in the subsequent analyses, and with 50 attempted changes to the tree (treebetN value) and 10 changes in the model parameters (Nbetsamp value) per run. BATWING runs were checked for a low autocorrelation between iterations using diagnostic tools provided with the software.

#### Historic migration of C3* carriers

In view of the restricted local occurrence of C3* haplogroups in South America and their concomitant absence from North and Central America, we set out to determine which migration rates between appropriately sized ancestral populations would have yielded a similar pattern of genetic differentiation. To this end, we considered three simplified models of population divergence. In scenario SA (‘South America only’), we combined those two sampling sites where C3* carriers were found into a single extant population of 84 individuals (14 C3*, 70 Q*, designated SA/C+) while the remaining 927 Q* carriers were merged into a single second population (SA/C−). Both populations were then assumed to have diverged 12,000 YBP (i.e. the likely time of the first human entry into South America). In scenario BA (‘both Americas’), we instead assumed that the ancestors of SA/C− diverged from those of the 355 confirmed Q* carriers from Mexico and North America [67], [69] (designated NA/C−) at 12,000 YBP whereas their joint common ancestral population diverged from the ancestors of SA/C+ at 15,000 YBP (i.e. the likely time of first human entry into the American continent). Finally, in scenario AA (‘all Americas’), we assumed that the ancestors of SA/C+ split from those of SA/C− and NA/C− at 15,000 YBP, with no additional divergence event thereafter. The likely population histories were then inferred using a coalescent model that allowed for migration between populations. Approximate Bayesian computation as implemented in the popABC software [107] was used to obtain the posterior distribution of the migration rates under the respective model. To this end, we simulated 500 million datasets each for scenarios SA and AA, and 300 million data sets for BA, accepting limits imposed by the computer memory required. A uniform prior on [0,0.2] was used for each migration rate. The mutation rate between the two haplogroups was set to 10^−8^ to emulate a SNP that defines haplogroups. The fixed effective population sizes were set equal to the corresponding sample sizes. We assumed a generation time of 30 years and a population growth rate per generation of 0.5%. This led to ancestral population sizes of 138 in scenario SA, 174 and 113 in scenario BA, and 113 in scenario AA. The posterior distribution of each migration rate was obtained from those 100 simulations that were closest to the observed data for two summary statistics, namely the number of alleles per population and the gene diversity. We used only the 100 closest simulations to estimate the posterior distributions of migration rates in order to avoid bias. In fact, the actually observed separation between C3* carriers and non-carriers represented such an extreme and rare event that it not even distantly resembled the vast majority of simulations. Note that, in three-population scenario BA, migration into one population combines migration from both other populations. For example, the migration rate into SA/C− combines migration from SA/C+ and from NA/C−. We repeated the above analyses assuming ten-fold larger effective population sizes (scenarios SA-10x and AA-10x). Owing to computing restrictions, however, we were only able to simulate 50 million datasets for each scenario, i.e. ten times less than for the original scenarios. The posterior distribution of the migration rate was then estimated from those 10 simulations with summary statistics closest to those observed in the real data. With scenario BA, a ten-fold increase of the effective population size turned out to be computationally infeasible.

#### Generation of geographic maps

Geographic maps were generated in R v2.14.0 [97] using packages *maps* v2.2-2, *mapproj* v1.1-8.3 and *mapdata* v2.2-0. The latter included an amended version of the CIA World Data Bank II. Pie charts were generated using the *draw.circle* and *floating.pie* functions of the *plotrix* v3.3-1 package.

### Web resources

R software: http://www.r-project.org/

*map*, *mapdata*, *mapproj*, *plotrix* packages: http://cran.r-project.org/

NETWORK software: http://www.fluxus-engineering.com/sharenet.htm

Arlequin software: http://cmpg.unibe.ch/software/arlequin35/

BATWING software: http://www.mas.ncl.ac.uk/~nijw/

popABC software: http://code.google.com/p/popabc/

YHRD: http://www.yhrd.org/

CIA World Data Bank II: http://www.evl.uic.edu/pape/data/WDB/

## Supporting Information

Figure S1 Language group per sampling site. Groups of spoken language at each sampling site. Language classification follows Ruhlen [44]. Yellow: Andean languages; green: Chibchan-Paezan languages; red: Equatorial-Tucanoan languages; blue: Ge-Pano-Carib languages; pink: Wao-Tiriro isolate.
(PDF)

Figure S2 Geographic clustering of sampling sites. Sampling sites were assigned to two types of geography-based cluster. Fine clustering A: A1 (“Patagonia”), A2 (“Central South America”), A3 (“El Beni/Rondonia”), A4 (“Northwest South America”), A5 (“Northern Amazon”) and A6 (“Southern Amazon”). Broad clustering B: B1 (“Highland”), B2 (“North Lowland”) and B3 (“South Lowland”).
(PDF)

Figure S3 Spatial autocorrelation analysis within geographic clusters. Spatial autocorrelation analyses were carried out separately for each geographic cluster. Please refer to main text for cluster definition and Figure S2 and Table S1 for cluster assignment of sampling sites. Top: fine clustering A; bottom: broad clustering B; filled circles: significant autocorrelation (P<0.05); empty circles: non-significant autocorrelation.
(PDF)

Figure S4 Median-joining network of 977 South American Y-STR haplotypes carrying Y-SNP haplogroup Q (data from this publication). The median–joining network was based upon markers DYS19, DYS389I, DYS389II, DYS390, DYS391, DYS392, DYS393 (see Materials and Methods for details). Color coding according to geography-based fine clustering A.
(PDF)

Figure S5 Median-joining network of 977 South American Y-STR haplotypes carrying Y-SNP haplogroup Q (data from this publication). Color coding according to geography-based broad clustering B (see Figure S4 for more details).
(PDF)

Figure S6 Median-joining network of 977 South American Y-STR haplotypes carrying Y-SNP haplogroup Q (data from this publication). Color coding according to haplogroup (see Figure S4 for more details).
(PDF)

Figure S7 Median-joining network of 977 South American Y-STR haplotypes carrying Y-SNP haplogroup Q (data from this publication). Color coding according to language classification following Ruhlen [44] (see Figure S4 for more details).
(PDF)

Figure S8 European sampling sites from the Y Chromosome Haplotype Reference Database (YHRD). For each European sampling site used in the comparative AMOVA analysis of European Y-chromosomal genetic diversity, its geographic location and its assignment to six broad geographic regions is depicted.
(PDF)

Figure S9 Y-SNP subgroups to C3* and Q*. Updated human Y-chromosomal haplogroup tree according to [65]. Where available, RefSeq numbers (according to NCBI dbSNP build37.3) of haplogroup-defining SNPs are given in parentheses.
(PDF)

Figure S10 Migration rate distribution under scenario SA. Shown are the prior and ABC-derived posterior distributions of migration rates under scenario SA. In this scenario, all individuals of the two sampling sites with C3* carriers were merged into one population (SA/C+) and all remaining individuals were merged into a single second population (SA/C−; see Materials and Methods in the main text for details). A: migration rate into SA/C+; B: migration rate into SA/C−. White bars: prior distribution (obtained from 10,000 randomly selected simulated datasets); black bars: posterior distribution (obtained from those 100 simulated datasets that were closest to the original dataset with respect to the number of alleles and the gene diversity in a population.
(PDF)

Figure S11 Migration rate distribution under scenario AA. Shown are the prior and ABC-derived posterior distributions of migration rates under scenario AA. In this scenario, all individuals of the two sampling sites with C3* carriers were merged into one population (SA/C+) and all remaining individuals were merged (SA/C−) together with all Q* carriers from two previous studies on North and Central American natives (NA/C−) into a single second population (see Materials and Methods in the main text for details). A: migration rate into SA/C+; B: migration rate into joint population of SA/C− and NA/C−. White bars: prior distribution (obtained from 10,000 randomly selected simulated datasets); black bars: posterior distribution (obtained from those 100 simulated datasets that were closest to the original dataset with respect to the number of alleles and the gene diversity in a population.
(PDF)

Figure S12 Migration rate distribution under scenario BA. Shown are the prior and ABC-derived posterior distributions of migration rates under scenario BA. In this scenario, all individuals of the two sampling sites with C3* carriers were merged into one population (SA/C+) and all remaining individuals from our study were merged into a single second population (SA/C−). All Q* carriers from two previous studies on North and Central American natives were combined in a third population (NA/C−; see Materials and Methods in the main text for details). A: migration rate into SA/C+; B: migration rate into SA/C−; C: migration rate into NA/C−; D: migration rate into the ancestral population of SA/C− and NA/C−. White bars: prior distribution (obtained from 10,000 randomly selected simulated datasets); black bars: posterior distribution (obtained from those 100 simulated datasets that were closest to the original dataset with respect to number of alleles and gene diversity in a population.
(PDF)

Figure S13 Migration rate distribution under scenario SA-10x. Shown are the prior and ABC-derived posterior distributions of migration rates under scenario SA. In this scenario, all individuals of the two sampling sites with C3* carriers were merged into one population (SA/C+) and all remaining individuals were merged into a single second population (SA/C−; see Materials and Methods in the main text for details). A: migration rate into SA/C+; B: migration rate into SA/C−. White bars: prior distribution (obtained from 10,000 randomly selected simulated datasets); black bars: posterior distribution (obtained from those 10 simulated datasets that were closest to the original dataset with respect to the number of alleles and the gene diversity in a population.
(PDF)

Figure S14 Migration rate distribution under scenario AA-10x. Shown are the prior and ABC-derived posterior distributions of migration rates under scenario AA. In this scenario, all individuals of the two sampling sites with C3* carriers were merged into one population (SA/C+) and all remaining individuals were merged (SA/C−) together with all Q* carriers from two previous studies on North and Central American natives (NA/C−) into a single second population (see Materials and Methods in the main text for details). A: migration rate into SA/C+; B: migration rate into joint population of SA/C− and NA/C−. White bars: prior distribution (obtained from 10,000 randomly selected simulated datasets); black bars: posterior distribution (obtained from those 10 simulated datasets that were closest to the original dataset with respect to the number of alleles and the gene diversity in a population.
(PDF)

Table S1 Sampling site characteristics. For each site, the sample size, geographic location, tribal and linguistic assignment and haplotype characteristics are given.
(DOCX)

Table S2 Mutation rate estimates for Y-STRs studied. For each marker, the average mutation rate used in this study in this study is given according to http://www.yhrd.org/Research/Loci (release 42, January 11, 2013).
(DOCX)

Table S3 Y-SNP haplogroup and language group. Cross-tabulation of haplogroup of carried haplotype *vs* group of spoken language per individual. Results are based on all 1011 samples.
(DOCX)

Table S4 European sampling sites for comparative analysis. For each sampling site that has been used in the comparative analysis with South-America, the country and region of origin as well as its geographic position, its regional assignment following [7] and the sample size are given. Sites from cities with more than 500,000 inhabitants were not included in the analysis.
(DOCX)

Table S5 Haplotypes of all 1011 studied Amerindian samples.
(XLS)

Table S6 Haplotypes of all included C3* reference samples from the literature.
(XLS)

## References

[1] Cavalli-Sforza LL (1997) Genes, peoples, and languages. Proceedings of the National Academy of Sciences of the United States of America 94: 7719–7724. 9223254 10.1073/pnas.94.15.7719 PMC33682

[2] Comas D, Bosch E, Calafell F (2008) Human genetics and languages. Chichester: Wiley.

[3] Jobling MA, Hurles ME, Tyler-Smith C (2004) Human Evolutionary Genetics: Origins, Peoples and Disease: Garland Publishing.

[4] Barbujani G, Sokal RR (1990) Zones of sharp genetic change in Europe are also linguistic boundaries. Proceedings of the National Academy of Sciences of the United States of America 87: 1816–1819. 2308939 10.1073/pnas.87.5.1816 PMC53574

[5] Lao O, Lu TT, Nothnagel M, Junge O, Freitag-Wolf S, et al (2008) Correlation between genetic and geographic structure in Europe. Current biology : CB 18: 1241–1248. 18691889 10.1016/j.cub.2008.07.049

[6] Novembre J, Johnson T, Bryc K, Kutalik Z, Boyko AR, et al (2008) Genes mirror geography within Europe. Nature 456: 98–101. 18758442 10.1038/nature07331 PMC2735096

[7] Roewer L, Croucher PJ, Willuweit S, Lu TT, Kayser M, et al (2005) Signature of recent historical events in the European Y-chromosomal STR haplotype distribution. Human genetics 116: 279–291. 15660227 10.1007/s00439-004-1201-z

[8] Rosser ZH, Zerjal T, Hurles ME, Adojaan M, Alavantic D, et al (2000) Y-chromosomal diversity in Europe is clinal and influenced primarily by geography, rather than by language. American journal of human genetics 67: 1526–1543. 11078479 10.1086/316890 PMC1287948

[9] Kayser M, Lao O, Anslinger K, Augustin C, Bargel G, et al (2005) Significant genetic differentiation between Poland and Germany follows present-day political borders, as revealed by Y-chromosome analysis. Human genetics 117: 428–443. 15959808 10.1007/s00439-005-1333-9

[10] Lahermo P, Sajantila A, Sistonen P, Lukka M, Aula P, et al (1996) The genetic relationship between the Finns and the Finnish Saami (Lapps): analysis of nuclear DNA and mtDNA. Am J Hum Genet 58: 1309–1322. 8651309 PMC1915079

[11] Ploski R, Wozniak M, Pawlowski R, Monies DM, Branicki W, et al (2002) Homogeneity and distinctiveness of Polish paternal lineages revealed by Y chromosome microsatellite haplotype analysis. Human genetics 110: 592–600. 12107446 10.1007/s00439-002-0728-0

[12] Perez-Lezaun A, Calafell F, Comas D, Mateu E, Bosch E, et al (1999) Sex-specific migration patterns in Central Asian populations, revealed by analysis of Y-chromosome short tandem repeats and mtDNA. Am J Hum Genet 65: 208–219. 10364534 10.1086/302451 PMC1378092

[13] Poloni ES, Semino O, Passarino G, Santachiara-Benerecetti AS, Dupanloup I, et al (1997) Human genetic affinities for Y-chromosome P49a,f/TaqI haplotypes show strong correspondence with linguistics. Am J Hum Genet 61: 1015–1035. 9346874 10.1086/301602 PMC1716025

[14] Kalaydjieva L, Calafell F, Jobling MA, Angelicheva D, de Knijff P, et al (2001) Patterns of inter- and intra-group genetic diversity in the Vlax Roma as revealed by Y chromosome and mitochondrial DNA lineages. European journal of human genetics : EJHG 9: 97–104. 11313742 10.1038/sj.ejhg.5200597

[15] Larmuseau MH, Vanoverbeke J, Gielis G, Vanderheyden N, Larmuseau HF, et al (2012) In the name of the migrant father-Analysis of surname origins identifies genetic admixture events undetectable from genealogical records. Heredity 109: 90–95. 22511074 10.1038/hdy.2012.17 PMC3400745

[16] Rodig H, Roewer L, Gross A, Richter T, de Knijff P, et al (2008) Evaluation of haplotype discrimination capacity of 35 Y-chromosomal short tandem repeat loci. Forensic science international 174: 182–188. 17543484 10.1016/j.forsciint.2007.04.223

[17] El-Sibai M, Platt DE, Haber M, Xue Y, Youhanna SC, et al (2009) Geographical structure of the Y-chromosomal genetic landscape of the Levant: a coastal-inland contrast. Annals of human genetics 73: 568–581. 19686289 10.1111/j.1469-1809.2009.00538.x PMC3312577

[18] Haber M, Platt DE, Badro DA, Xue Y, El-Sibai M, et al (2011) Influences of history, geography, and religion on genetic structure: the Maronites in Lebanon. European journal of human genetics : EJHG 19: 334–340. 21119711 10.1038/ejhg.2010.177 PMC3062011

[19] Jin HJ, Tyler-Smith C, Kim W (2009) The peopling of Korea revealed by analyses of mitochondrial DNA and Y-chromosomal markers. PLoS ONE 4: e4210 doi:10.1371/journal.pone.0004210. 19148289 10.1371/journal.pone.0004210 PMC2615218

[20] Xue Y, Zerjal T, Bao W, Zhu S, Shu Q, et al (2006) Male demography in East Asia: a north-south contrast in human population expansion times. Genetics 172: 2431–2439. 16489223 10.1534/genetics.105.054270 PMC1456369

[21] de Filippo C, Barbieri C, Whitten M, Mpoloka SW, Gunnarsdottir ED, et al (2011) Y-chromosomal variation in sub-Saharan Africa: insights into the history of Niger-Congo groups. Molecular biology and evolution 28: 1255–1269. 21109585 10.1093/molbev/msq312 PMC3561512

[22] Wood ET, Stover DA, Ehret C, Destro-Bisol G, Spedini G, et al (2005) Contrasting patterns of Y chromosome and mtDNA variation in Africa: evidence for sex-biased demographic processes. European journal of human genetics : EJHG 13: 867–876. 15856073 10.1038/sj.ejhg.5201408

[23] Hagelberg E, Kayser M, Nagy M, Roewer L, Zimdahl H, et al (1999) Molecular genetic evidence for the human settlement of the Pacific: analysis of mitochondrial DNA, Y chromosome and HLA markers. Philosophical transactions of the Royal Society of London Series B, Biological sciences 354: 141–152. 10091254 10.1098/rstb.1999.0367 PMC1692446

[24] Kayser M, Brauer S, Cordaux R, Casto A, Lao O, et al (2006) Melanesian and Asian origins of Polynesians: mtDNA and Y chromosome gradients across the Pacific. Molecular biology and evolution 23: 2234–2244. 16923821 10.1093/molbev/msl093

[25] Kayser M, Brauer S, Weiss G, Schiefenhovel W, Underhill P, et al (2003) Reduced Y-chromosome, but not mitochondrial DNA, diversity in human populations from West New Guinea. American journal of human genetics 72: 281–302. 12532283 10.1086/346065 PMC379223

[26] Kayser M, Choi Y, van Oven M, Mona S, Brauer S, et al (2008) The impact of the Austronesian expansion: evidence from mtDNA and Y chromosome diversity in the Admiralty Islands of Melanesia. Molecular biology and evolution 25: 1362–1374. 18390477 10.1093/molbev/msn078

[27] Mona S, Tommaseo-Ponzetta M, Brauer S, Sudoyo H, Marzuki S, et al (2007) Patterns of Y-chromosome diversity intersect with the Trans-New Guinea hypothesis. Molecular biology and evolution 24: 2546–2555. 17846104 10.1093/molbev/msm187

[28] Kayser M, Krawczak M, Excoffier L, Dieltjes P, Corach D, et al (2001) An extensive analysis of Y-chromosomal microsatellite haplotypes in globally dispersed human populations. American journal of human genetics 68: 990–1018. 11254455 10.1086/319510 PMC1275652

[29] Shi W, Ayub Q, Vermeulen M, Shao RG, Zuniga S, et al (2010) A worldwide survey of human male demographic history based on Y-SNP and Y-STR data from the HGDP-CEPH populations. Molecular biology and evolution 27: 385–393. 19822636 10.1093/molbev/msp243 PMC2806244

[30] Forster P, Renfrew C (2011) Evolution. Mother tongue and Y chromosomes. Science 333: 1390–1391. 21903800 10.1126/science.1205331

[31] Hurles ME, Veitia R, Arroyo E, Armenteros M, Bertranpetit J, et al (1999) Recent male-mediated gene flow over a linguistic barrier in Iberia, suggested by analysis of a Y-chromosomal DNA polymorphism. Am J Hum Genet 65: 1437–1448. 10521311 10.1086/302617 PMC1288297

[32] Bosch E, Calafell F, Gonzalez-Neira A, Flaiz C, Mateu E, et al (2006) Paternal and maternal lineages in the Balkans show a homogeneous landscape over linguistic barriers, except for the isolated Aromuns. Ann Hum Genet 70: 459–487. 16759179 10.1111/j.1469-1809.2005.00251.x

[33] Goebel T, Waters MR, O'Rourke DH (2008) The late Pleistocene dispersal of modern humans in the Americas. Science 319: 1497–1502. 18339930 10.1126/science.1153569

[34] Greenberg JH (1987) Language in the Americas. Stanford, CA: Stanford University Press.

[35] Lanata JL, Martino L, Osella A, Garcia-Herbst A (2008) Demographic conditions necessary to colonize new spaces: the case for early human dispersal in the Americas. World Archaeology 40: 520–537.

[36] Rothhammer F, Silva C (1989) Peopling of Andean South America. American journal of physical anthropology 78: 403–410. 2648861 10.1002/ajpa.1330780308

[37] Waters MR, Stafford TW Jr, McDonald HG, Gustafson C, Rasmussen M, et al (2011) Pre-Clovis mastodon hunting 13,800 years ago at the Manis site, Washington. Science 334: 351–353. 22021854 10.1126/science.1207663

[38] Dillehay TD, Ramirez C, Pino M, Collins MB, Rossen J, et al (2008) Monte Verde: seaweed, food, medicine, and the peopling of South America. Science 320: 784–786. 18467586 10.1126/science.1156533

[39] Salzano FM, Callegari-Jacques SM (1988) South American Indians: A case study in evolution. Oxford: Clarendon Press.

[40] Silverman H, Isbell WE (2008) Handbook of South American Archaeology. New York: Springer.

[41] Balter M (2008) Archaeology. Ancient algae suggest sea route for first Americans. Science 320: 729. 18467561 10.1126/science.320.5877.729

[42] Kirk RL (1979) Genetic differentiation in Australia and its bearing on the origin of the first Americans. In: Laughlin WS, Harper AB, editors. The first Americans: Origins, affinities, and adaptations. Stuttgart: Gustav Fischer. pp. 211–237.

[43] Estrada E, Meggers BJ, Evans C (1962) Possible Transpacific Contact on the Coast of Ecuador. Science 135: 371–372. 17782632 10.1126/science.135.3501.371

[44] Ruhlen M (1991) A Guide to the World Languages. Stanford, CA: Stanford University Press.

[45] Goddard I (1996) Languages. Handbook of North American Indians. Washington, DC: Smithsonian Institution.

[46] Campbell L (1997) American Indian languages: the historical linguistics of Native America. New York: Oxford University Press.

[47] Mithun M (1999) The languages of native North America. Cambridge, UK: Cambridge University Press.

[48] Bolnick DA, Shook BA, Campbell L, Goddard I (2004) Problematic use of Greenberg's linguistic classification of the Americas in studies of Native American genetic variation. American journal of human genetics 75: 519–522. 15284953 10.1086/423452 PMC1182033

[49] Hunley KL, Cabana GS, Merriwether DA, Long JC (2007) A formal test of linguistic and genetic coevolution in native Central and South America. American journal of physical anthropology 132: 622–631. 17205551 10.1002/ajpa.20542

[50] Yang NN, Mazieres S, Bravi C, Ray N, Wang S, et al (2010) Contrasting patterns of nuclear and mtDNA diversity in Native American populations. Annals of human genetics 74: 525–538. 20887376 10.1111/j.1469-1809.2010.00608.x

[51] Crawford MH (1998) The origins of Native Americans: Cambridge University Press.

[52] Fagundes NJ, Kanitz R, Eckert R, Valls AC, Bogo MR, et al (2008) Mitochondrial population genomics supports a single pre-Clovis origin with a coastal route for the peopling of the Americas. American journal of human genetics 82: 583–592. 18313026 10.1016/j.ajhg.2007.11.013 PMC2427228

[53] Kitchen A, Miyamoto MM, Mulligan CJ (2008) A three-stage colonization model for the peopling of the Americas. PLoS ONE 3: e1596 doi:10.1371/journal.pone.0001596. 18270583 10.1371/journal.pone.0001596 PMC2223069

[54] Tamm E, Kivisild T, Reidla M, Metspalu M, Smith DG, et al (2007) Beringian standstill and spread of Native American founders. PLoS ONE 2: e829 doi:10.1371/journal.pone.0000829. 17786201 10.1371/journal.pone.0000829 PMC1952074

[55] Tarazona-Santos E, Carvalho-Silva DR, Pettener D, Luiselli D, De Stefano GF, et al (2001) Genetic differentiation in South Amerindians is related to environmental and cultural diversity: evidence from the Y chromosome. American journal of human genetics 68: 1485–1496. 11353402 10.1086/320601 PMC1226135

[56] Wang S, Lewis CM, Jakobsson M, Ramachandran S, Ray N, et al (2007) Genetic variation and population structure in native Americans. PLoS Genet 3: e185 doi:10.1371/journal.pgen.0030185. 18039031 10.1371/journal.pgen.0030185 PMC2082466

[57] Bortolini MC, Salzano FM, Thomas MG, Stuart S, Nasanen SP, et al (2003) Y-chromosome evidence for differing ancient demographic histories in the Americas. American journal of human genetics 73: 524–539. 12900798 10.1086/377588 PMC1180678

[58] Heckenberger MJ, Kuikuro A, Kuikuro UT, Russell JC, Schmidt M, et al (2003) Amazonia 1492: pristine forest or cultural parkland? Science 301: 1710–1714. 14500979 10.1126/science.1086112

[59] Lell JT, Sukernik RI, Starikovskaya YB, Su B, Jin L, et al (2002) The dual origin and Siberian affinities of Native American Y chromosomes. American journal of human genetics 70: 192–206. 11731934 10.1086/338457 PMC384887

[60] Pena SD, Santos FR, Bianchi NO, Bravi CM, Carnese FR, et al (1995) A major founder Y-chromosome haplotype in Amerindians. Nature genetics 11: 15–16. 7550307 10.1038/ng0995-15

[61] Underhill PA, Jin L, Zemans R, Oefner PJ, Cavalli-Sforza LL (1996) A pre-Columbian Y chromosome-specific transition and its implications for human evolutionary history. Proc Natl Acad Sci U S A 93: 196–200. 8552603 10.1073/pnas.93.1.196 PMC40205

[62] Bailliet G, Ramallo V, Muzzio M, Garcia A, Santos MR, et al (2009) Brief communication: Restricted geographic distribution for Y-Q* paragroup in South America. American journal of physical anthropology 140: 578–582. 19591214 10.1002/ajpa.21133

[63] Bisso-Machado R, Jota MS, Ramallo V, Paixao-Cortes VR, Lacerda DR, et al (2011) Distribution of Y-chromosome Q lineages in Native Americans. American journal of human biology : the official journal of the Human Biology Council 23: 563–566. 21544893 10.1002/ajhb.21173

[64] Jota MS, Lacerda DR, Sandoval JR, Vieira PP, Santos-Lopes SS, et al (2011) A new subhaplogroup of native American Y-Chromosomes from the Andes. American journal of physical anthropology 146: 553–559. 21913173 10.1002/ajpa.21519

[65] Karafet TM, Mendez FL, Meilerman MB, Underhill PA, Zegura SL, et al (2008) New binary polymorphisms reshape and increase resolution of the human Y chromosomal haplogroup tree. Genome Res 18: 830–838. 18385274 10.1101/gr.7172008 PMC2336805

[66] Zegura SL, Karafet TM, Zhivotovsky LA, Hammer MF (2004) High-resolution SNPs and microsatellite haplotypes point to a single, recent entry of Native American Y chromosomes into the Americas. Molecular biology and evolution 21: 164–175. 14595095 10.1093/molbev/msh009

[67] Schurr TG, Dulik MC, Owings AC, Zhadanov SI, Gaieski JB, et al (2012) Clan, language, and migration history has shaped genetic diversity in Haida and Tlingit populations from Southeast Alaska. American journal of physical anthropology 148: 422–435. 22549307 10.1002/ajpa.22068 PMC4335652

[68] Geppert M, Baeta M, Nunez C, Martinez-Jarreta B, Zweynert S, et al (2011) Hierarchical Y-SNP assay to study the hidden diversity and phylogenetic relationship of native populations in South America. Forensic science international Genetics 5: 100–104. 20932815 10.1016/j.fsigen.2010.08.016

[69] Sandoval K, Moreno-Estrada A, Mendizabal I, Underhill PA, Lopez-Valenzuela M, et al (2012) Y-chromosome diversity in Native Mexicans reveals continental transition of genetic structure in the Americas. Am J Phys Anthropol 148: 395–405. 22576278 10.1002/ajpa.22062

[70] Malyarchuk B, Derenko M, Denisova G, Wozniak M, Grzybowski T, et al (2010) Phylogeography of the Y-chromosome haplogroup C in northern Eurasia. Annals of human genetics 74: 539–546. 20726964 10.1111/j.1469-1809.2010.00601.x

[71] Toscanini U, Gusmao L, Berardi G, Gomes V, Amorim A, et al (2011) Male lineages in South American native groups: evidence of M19 traveling south. American journal of physical anthropology 146: 188–196. 21826635 10.1002/ajpa.21562

[72] Ruiz-Linares A, Ortiz-Barrientos D, Figueroa M, Mesa N, Munera JG, et al (1999) Microsatellites provide evidence for Y chromosome diversity among the founders of the New World. Proceedings of the National Academy of Sciences of the United States of America 96: 6312–6317. 10339584 10.1073/pnas.96.11.6312 PMC26878

[73] Kamp C, Hirschmann P, Voss H, Huellen K, Vogt PH (2000) Two long homologous retroviral sequence blocks in proximal Yq11 cause AZFa microdeletions as a result of intrachromosomal recombination events. Human molecular genetics 9: 2563–2572. 11030762 10.1093/hmg/9.17.2563

[74] Dulik MC, Owings AC, Gaieski JB, Vilar MG, Andre A, et al (2012) Y-chromosome analysis reveals genetic divergence and new founding native lineages in Athapaskan- and Eskimoan-speaking populations. Proc Natl Acad Sci U S A 109: 8471–8476. 22586127 10.1073/pnas.1118760109 PMC3365193

[75] Hammer MF, Karafet TM, Park H, Omoto K, Harihara S, et al (2006) Dual origins of the Japanese: common ground for hunter-gatherer and farmer Y chromosomes. Journal of human genetics 51: 47–58. 16328082 10.1007/s10038-005-0322-0

[76] Nonaka I, Minaguchi K, Takezaki N (2007) Y-chromosomal binary haplogroups in the Japanese population and their relationship to 16 Y-STR polymorphisms. Annals of human genetics 71: 480–495. 17274803 10.1111/j.1469-1809.2006.00343.x

[77] Tajima A, Hayami M, Tokunaga K, Juji T, Matsuo M, et al (2004) Genetic origins of the Ainu inferred from combined DNA analyses of maternal and paternal lineages. Journal of human genetics 49: 187–193. 14997363 10.1007/s10038-004-0131-x

[78] Chang YM, Perumal R, Keat PY, Kuehn DL (2007) Haplotype diversity of 16 Y-chromosomal STRs in three main ethnic populations (Malays, Chinese and Indians) in Malaysia. Forensic science international 167: 70–76. 16457976 10.1016/j.forsciint.2006.01.002

[79] Cinnioglu C, King R, Kivisild T, Kalfoglu E, Atasoy S, et al (2004) Excavating Y-chromosome haplotype strata in Anatolia. Human genetics 114: 127–148. 14586639 10.1007/s00439-003-1031-4

[80] Gayden T, Mirabal S, Cadenas AM, Lacau H, Simms TM, et al (2009) Genetic insights into the origins of Tibeto-Burman populations in the Himalayas. Journal of human genetics 54: 216–223. 19282873 10.1038/jhg.2009.14

[81] Kim SH, Han MS, Kim W (2010) Y chromosome homogeneity in the Korean population. International journal of legal medicine 124: 653–657. 20714743 10.1007/s00414-010-0501-1

[82] Kim YJ, Shin DJ, Kim JM, Jin HJ, Kwak KD, et al (2001) Y-chromosome STR haplotype profiling in the Korean population. Forensic science international 115: 231–237. 11074178 10.1016/s0379-0738(00)00332-7

[83] Kwak KD, Jin HJ, Shin DJ, Kim JM, Roewer L, et al (2005) Y-chromosomal STR haplotypes and their applications to forensic and population studies in east Asia. International journal of legal medicine 119: 195–201. 15856270 10.1007/s00414-004-0518-4

[84] Yunusbayev B, Metspalu M, Jarve M, Kutuev I, Rootsi S, et al (2012) The caucasus as an asymmetric semipermeable barrier to ancient human migrations. Molecular biology and evolution 29: 359–365. 21917723 10.1093/molbev/msr221

[85] Barbujani G (1997) DNA variation and language affinities. American journal of human genetics 61: 1011–1014. 9345113 10.1086/301620 PMC1716036

[86] Fuselli S, Tarazona-Santos E, Dupanloup I, Soto A, Luiselli D, et al (2003) Mitochondrial DNA diversity in South America and the genetic history of Andean highlanders. Molecular biology and evolution 20: 1682–1691. 12832631 10.1093/molbev/msg188

[87] Kohlrausch FB, Callegari-Jacques SM, Tsuneto LT, Petzl-Erler ML, Hill K, et al (2005) Geography influences microsatellite polymorphism diversity in Amerindians. American journal of physical anthropology 126: 463–470. 15386223 10.1002/ajpa.20042

[88] Reich D, Patterson N, Campbell D, Tandon A, Mazieres S, et al (2012) Reconstructing Native American population history. Nature 488: 370–374. 22801491 10.1038/nature11258 PMC3615710

[89] Beckerman S, Erickson PI, Yost J, Regalado J, Jaramillo L, et al (2009) Life histories, blood revenge, and reproductive success among the Waorani of Ecuador. Proc Natl Acad Sci U S A 106: 8134–8139. 19433797 10.1073/pnas.0901431106 PMC2688884

[90] Schurr TG (2004) The peopling of the New World: Perspectives from molecular anthropology. Annu Rev Anthropol 33: 551–583.

[91] Marino M, Sala A, Corach D (2007) Genetic attributes of the YHRD minimal haplotype in 10 provinces of Argentina. Forensic science international Genetics 1: 129–133. 19083743 10.1016/j.fsigen.2007.01.013

[92] Palha Tde J, Rodrigues EM, Dos Santos SE (2007) Y-chromosomal STR haplotypes in a population from the Amazon region, Brazil. Forensic science international 166: 233–239. 16439086 10.1016/j.forsciint.2005.12.012

[93] Palha TJ, Rodrigues EM, dos Santos SE (2010) Y-STR haplotypes of Native American populations from the Brazilian Amazon region. Forensic science international Genetics 4: e121–123. 20457062 10.1016/j.fsigen.2009.12.003

[94] Gusmao L, Butler JM, Carracedo A, Gill P, Kayser M, et al (2006) DNA Commission of the International Society of Forensic Genetics (ISFG): an update of the recommendations on the use of Y-STRs in forensic analysis. Forensic science international 157: 187–197. 15913936 10.1016/j.forsciint.2005.04.002

[95] Beleza S, Gusmao L, Lopes A, Alves C, Gomes I, et al (2006) Micro-phylogeographic and demographic history of Portuguese male lineages. Annals of human genetics 70: 181–194. 16626329 10.1111/j.1529-8817.2005.00221.x

[96] Zuccarelli G, Alechine E, Caputo M, Bobillo C, Corach D, et al (2011) Rapid screening for Native American mitochondrial and Y-chromosome haplogroups detection in routine DNA analysis. Forensic science international Genetics 5: 105–108. 20880766 10.1016/j.fsigen.2010.08.018

[97] R Development Core Team (2011) R: A language and environment for statistical computing. R Foundation for Statistical Computing, Vienna, Austria.

[98] Nei M (1987) Molecular evolutionary genetics. New York: Columbia University Press.

[99] Nei M, Tajima F (1981) DNA polymorphism detectable by restriction endonucleases. Genetics 97: 145–163. 6266912 10.1093/genetics/97.1.145 PMC1214380

[100] Cramér H (1946) Mathematical Methods of Statistics. Princeton, NJ: Princeton University Press.

[101] Excoffier L, Smouse PE (1994) Using allele frequencies and geographic subdivision to reconstruct gene trees within a species: molecular variance parsimony. Genetics 136: 343–359. 8138170 10.1093/genetics/136.1.343 PMC1205785

[102] Excoffier L, Smouse PE, Quattro JM (1992) Analysis of molecular variance inferred from metric distances among DNA haplotypes: application to human mitochondrial DNA restriction data. Genetics 131: 479–491. 1644282 10.1093/genetics/131.2.479 PMC1205020

[103] Excoffier L, Lischer HE (2010) Arlequin suite ver 3.5: a new series of programs to perform population genetics analyses under Linux and Windows. Molecular ecology resources 10: 564–567. 21565059 10.1111/j.1755-0998.2010.02847.x

[104] Sokal RR, Oden NL (1978) Spatial autocorrelation in biology. 1. Methodology. Biol J Linn Soc 10: 199–228.

[105] Bandelt HJ, Forster P, Rohl A (1999) Median-joining networks for inferring intraspecific phylogenies. Molecular biology and evolution 16: 37–48. 10331250 10.1093/oxfordjournals.molbev.a026036

[106] Wilson IJ, Weale ME, Balding DJ (2003) Inferences from DNA data: population histories, evolutionary processes and forensic match probabilities. Journal of the Royal Statistical Society: Series A 166: 155–188.

[107] Lopes JS, Balding D, Beaumont MA (2009) PopABC: a program to infer historical demographic parameters. Bioinformatics 25: 2747–2749. 19679678 10.1093/bioinformatics/btp487

