# Supplementary figures and images for: Continent-Wide Decoupling of Y-Chromosomal Genetic Variation from Language and Geography in Native South Americans
Source: PLoS Genet. 2013 Apr 11;9(4):e1003460. doi: 10.1371/journal.pgen.1003460 (PMC3623769; doi:10.1371/journal.pgen.1003460)

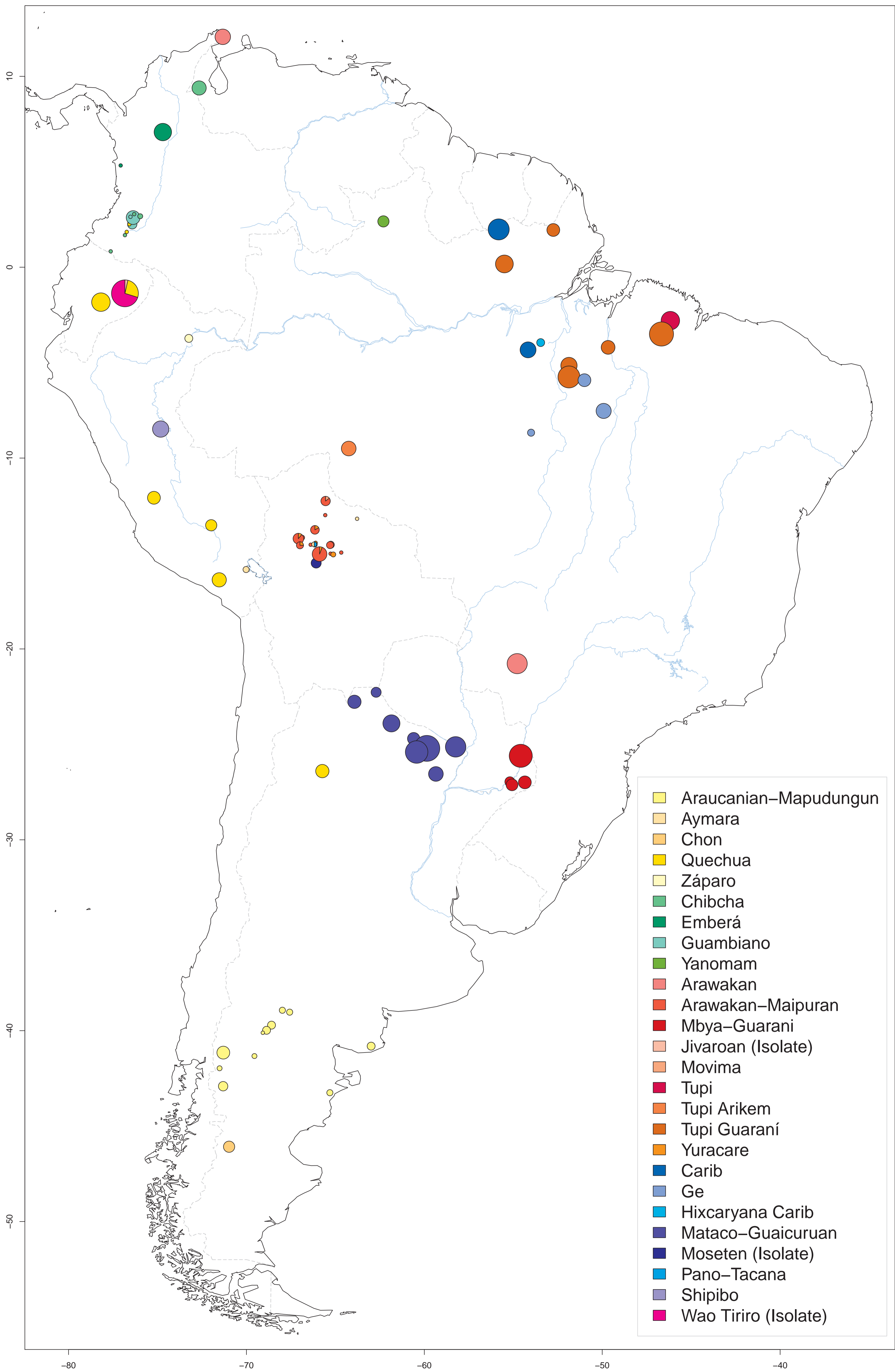

Supplement: Figure S1 — Language group per sampling site. Groups of spoken language at each sampling site. Language classification follows Ruhlen [44]. Yellow: Andean languages; green: Chibchan-Paezan languages; red: Equatorial-Tucanoan languages; blue: Ge-Pano-Carib languages; pink: Wao-Tiriro isolate. (PDF) [file pgen.1003460.s001.pdf]

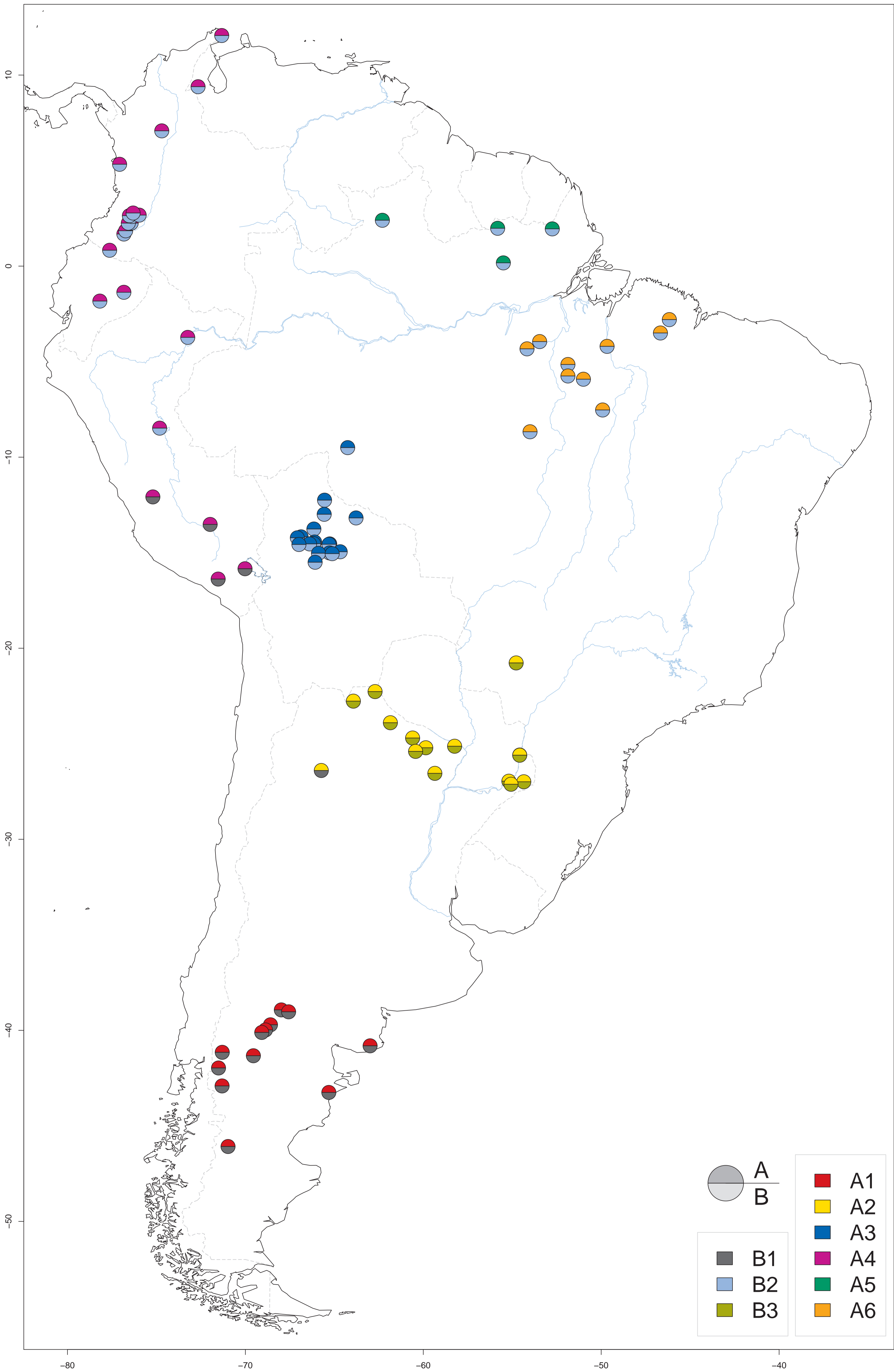

Supplement: Figure S2 — Geographic clustering of sampling sites. Sampling sites were assigned to two types of geography-based cluster. Fine clustering A: A1 (“Patagonia”), A2 (“Central South America”), A3 (“El Beni/Rondonia”), A4 (“Northwest South America”), A5 (“Northern Amazon”) and A6 (“Southern Amazon”). Broad clustering B: B1 (“Highland”), B2 (“North Lowland”) and B3 (“South Lowland”). (PDF) [file pgen.1003460.s002.pdf]

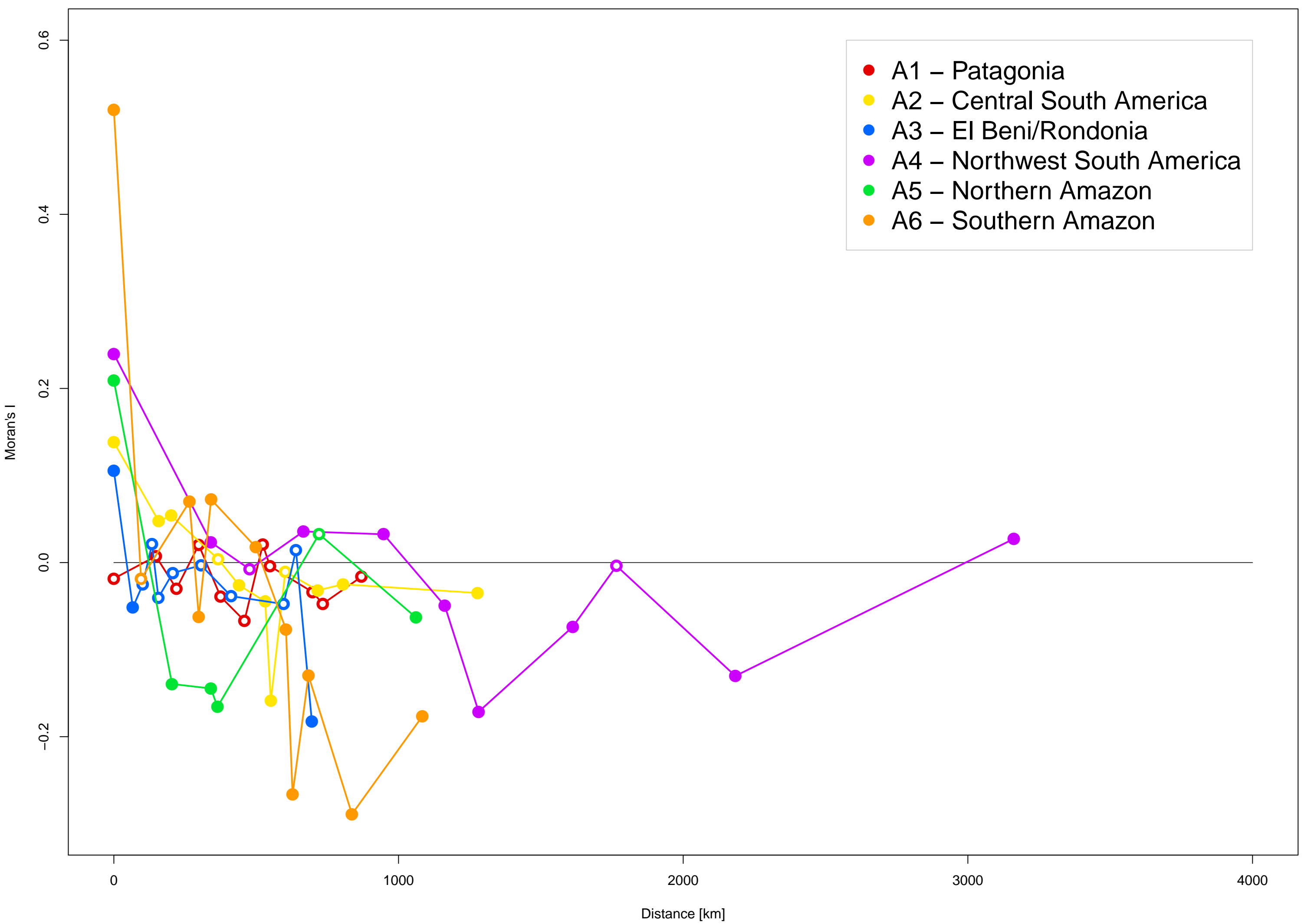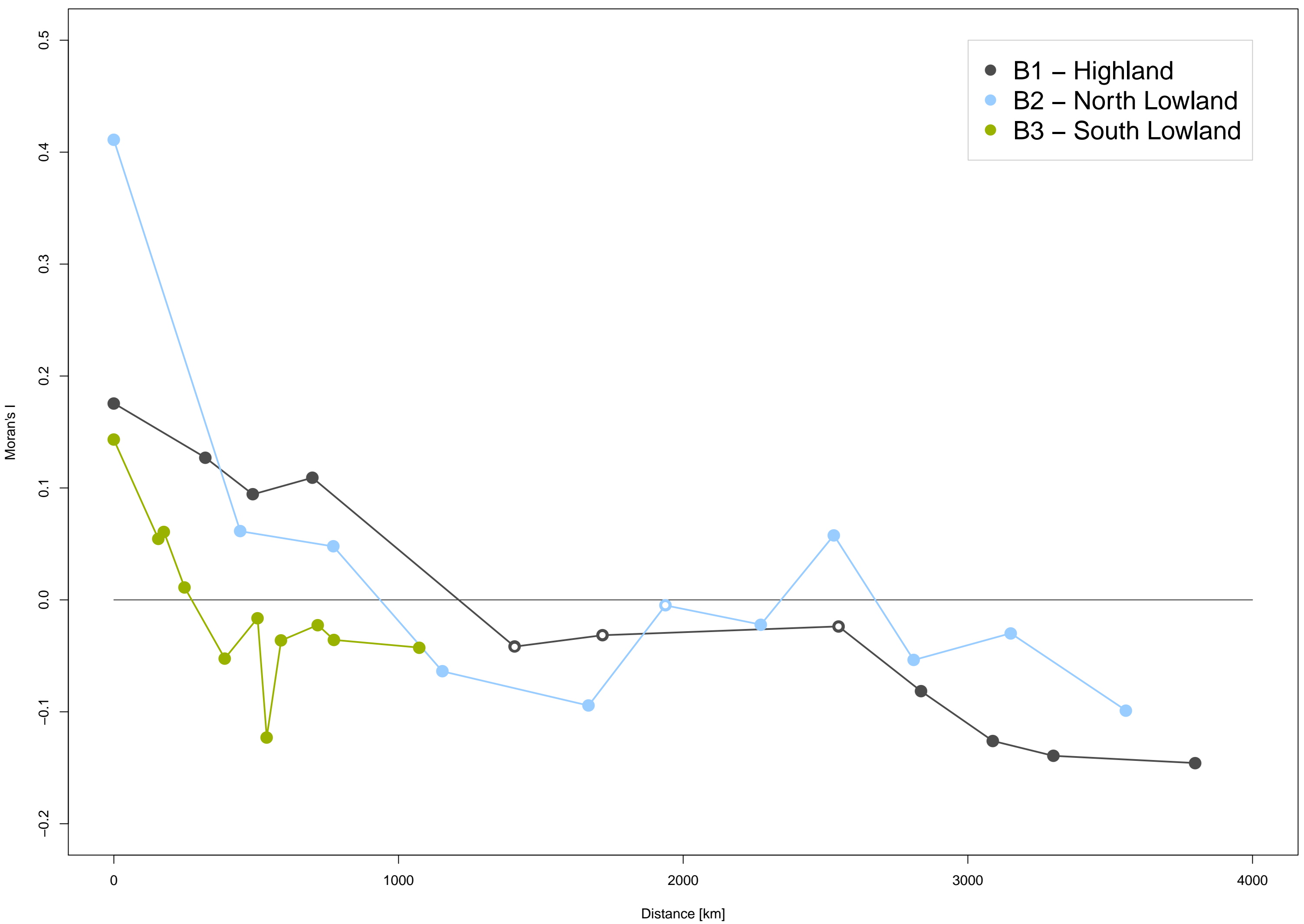

Supplement: Figure S3 — Spatial autocorrelation analysis within geographic clusters. Spatial autocorrelation analyses were carried out separately for each geographic cluster. Please refer to main text for cluster definition and Figure S2 and Table S1 for cluster assignment of sampling sites. Top: fine clustering A; bottom: broad clustering B; filled circles: significant autocorrelation (P<0.05); empty circles: non-significant autocorrelation. (PDF) [file pgen.1003460.s003.pdf]

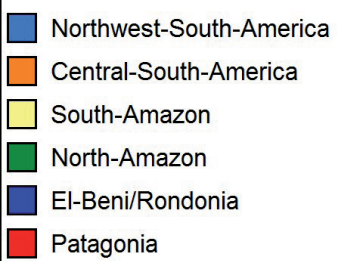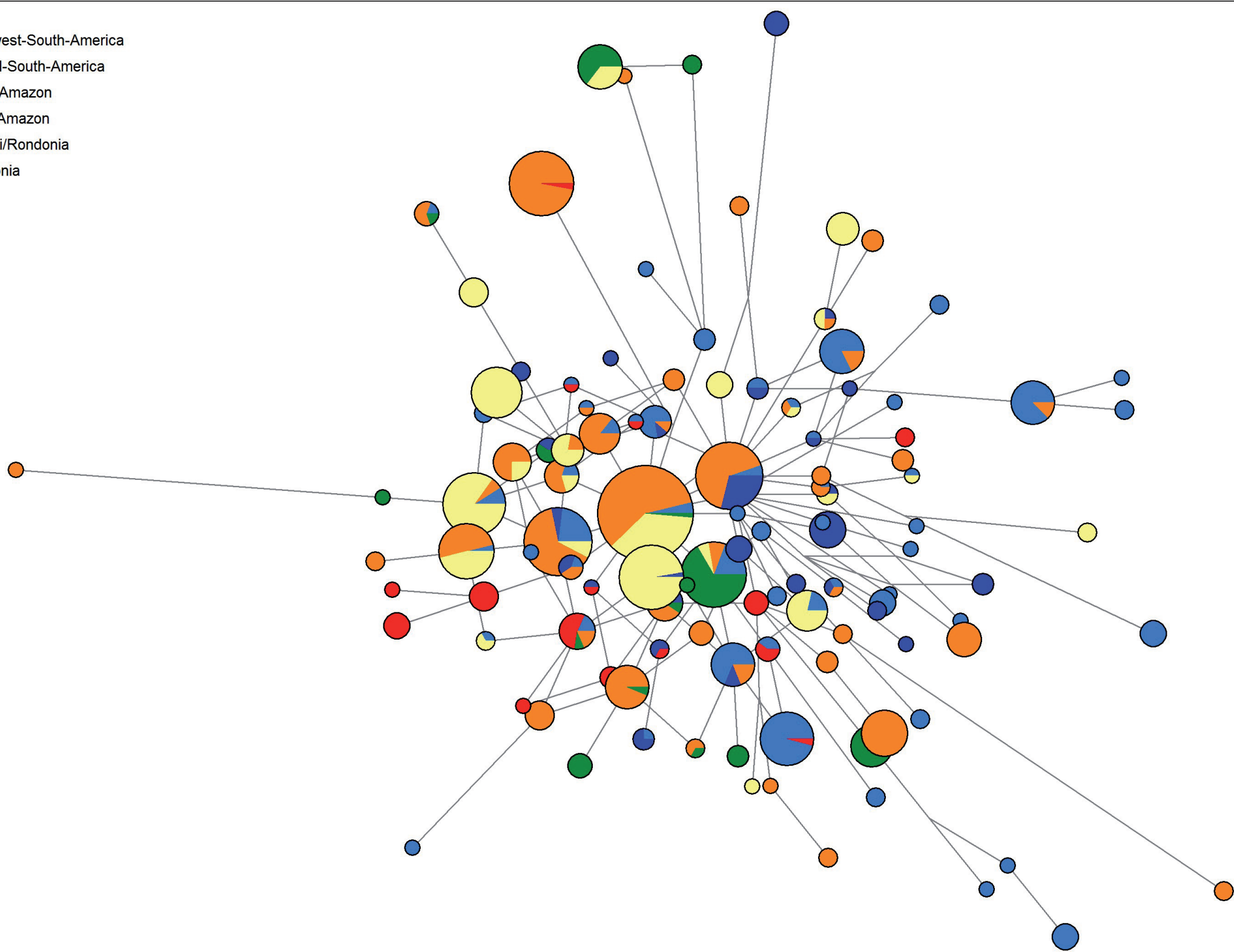

Supplement: Figure S4 — Median-joining network of 977 South American Y-STR haplotypes carrying Y-SNP haplogroup Q (data from this publication). The median–joining network was based upon markers DYS19, DYS389I, DYS389II, DYS390, DYS391, DYS392, DYS393 (see Materials and Methods for details). Color coding according to geography-based fine clustering A. (PDF) [file pgen.1003460.s004.pdf]

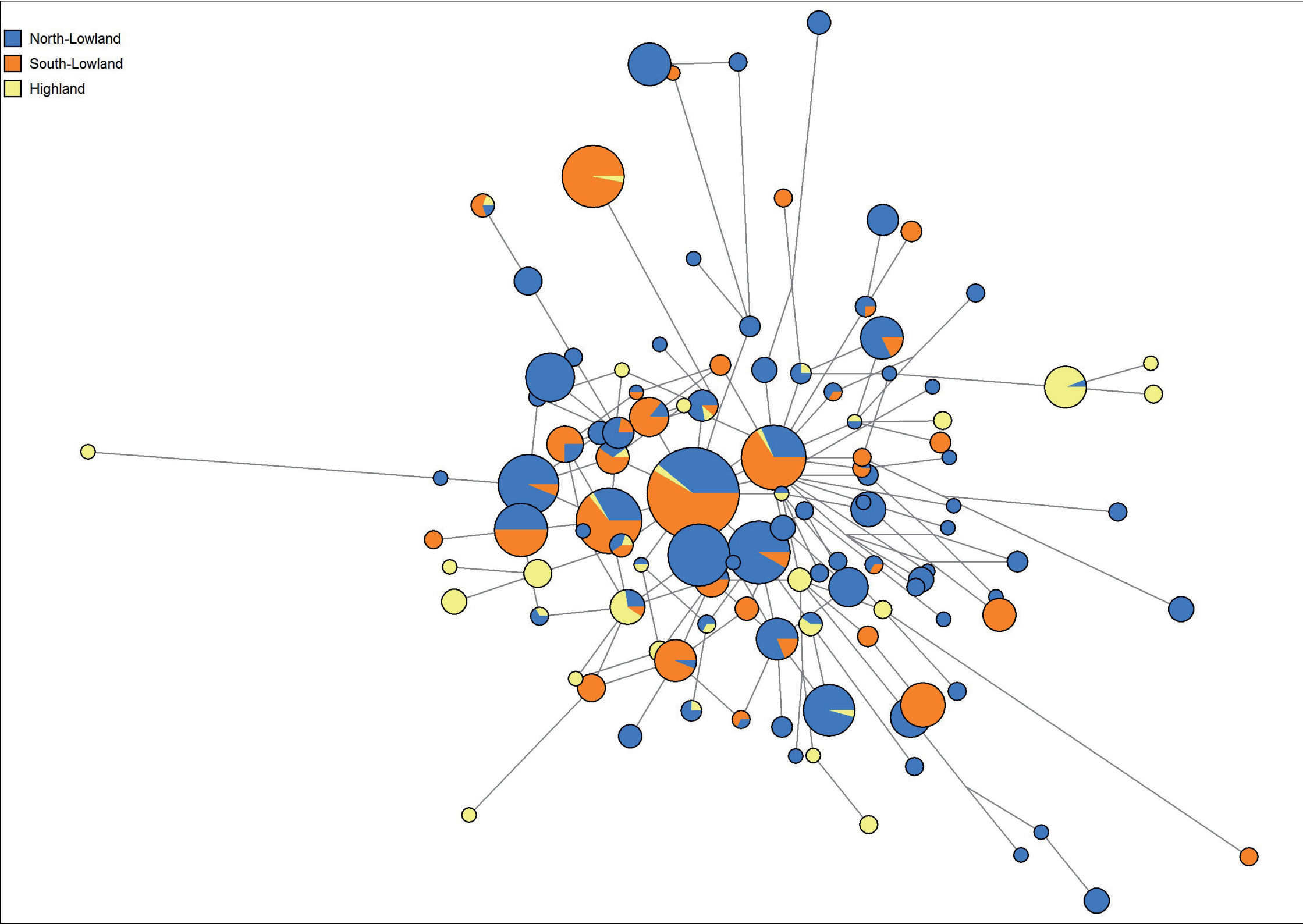

Supplement: Figure S5 — Median-joining network of 977 South American Y-STR haplotypes carrying Y-SNP haplogroup Q (data from this publication). Color coding according to geography-based broad clustering B (see Figure S4 for more details). (PDF) [file pgen.1003460.s005.pdf]

Q1a3a  
Q1a3  
Q1a3a1

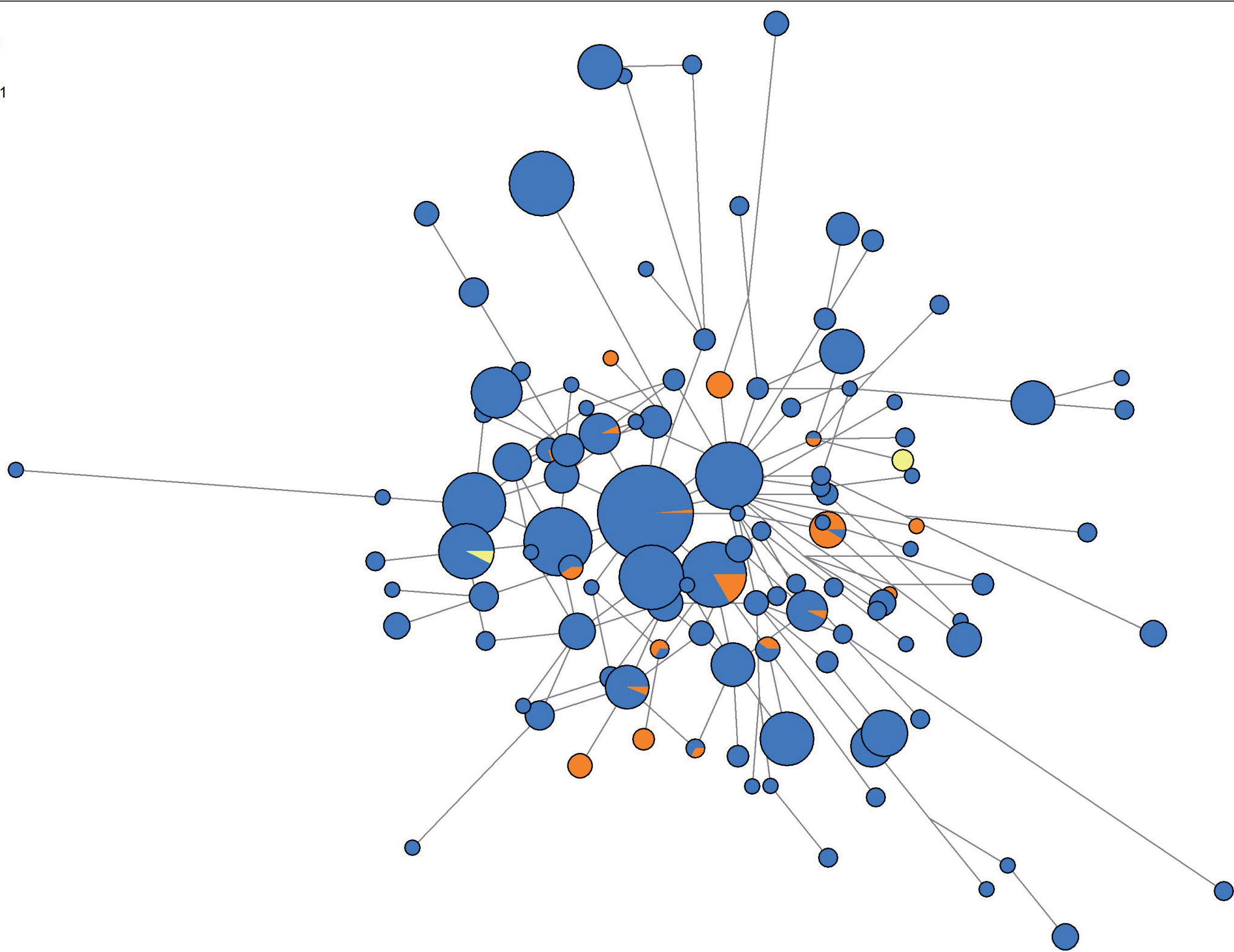

Supplement: Figure S6 — Median-joining network of 977 South American Y-STR haplotypes carrying Y-SNP haplogroup Q (data from this publication). Color coding according to haplogroup (see Figure S4 for more details). (PDF) [file pgen.1003460.s006.pdf]

- Equatorial-Tucanoan
- Chibchan-Paezan
- Ge-Pano-Carib
- Andean
- Isolate

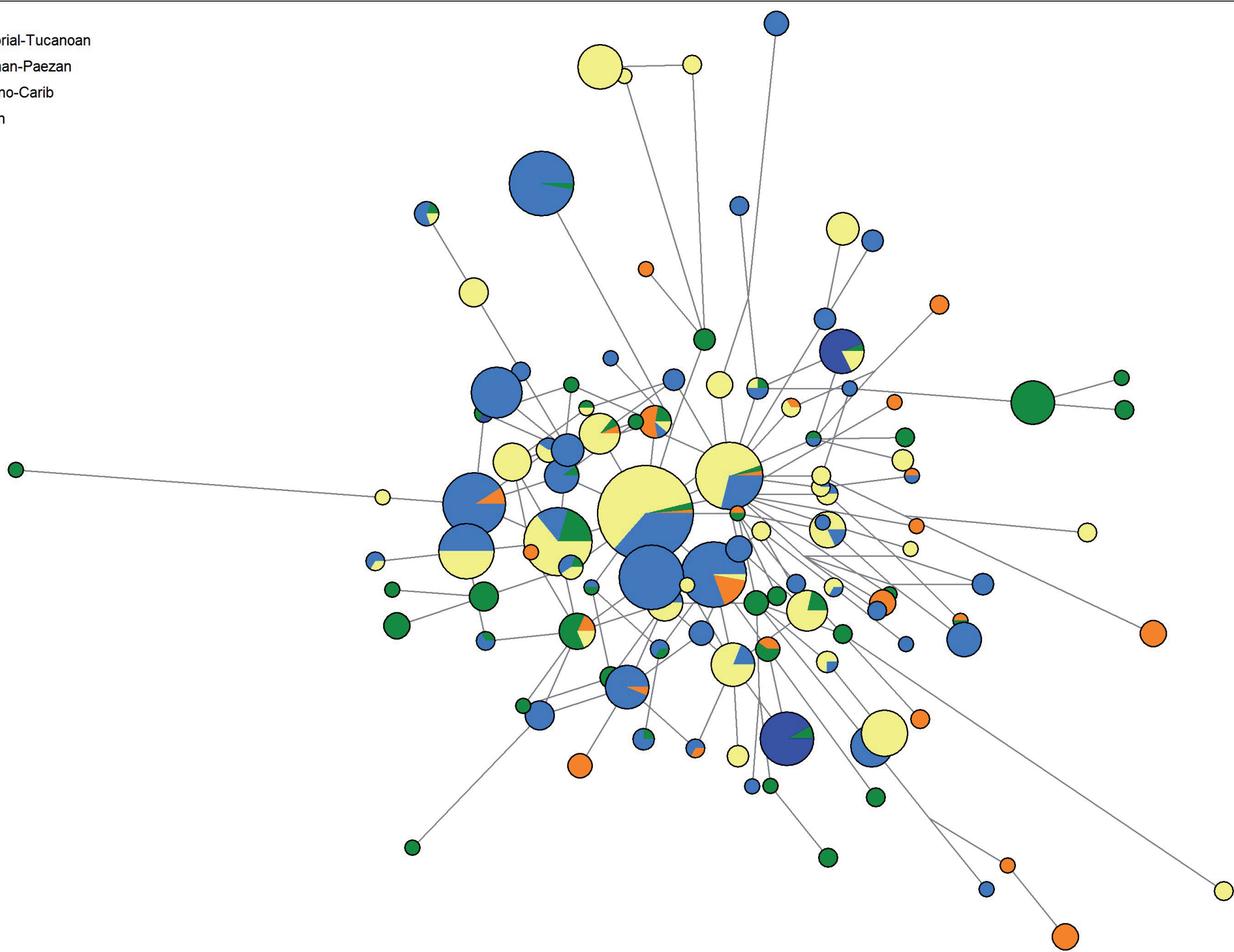

Supplement: Figure S7 — Median-joining network of 977 South American Y-STR haplotypes carrying Y-SNP haplogroup Q (data from this publication). Color coding according to language classification following Ruhlen [44] (see Figure S4 for more details). (PDF) [file pgen.1003460.s007.pdf]

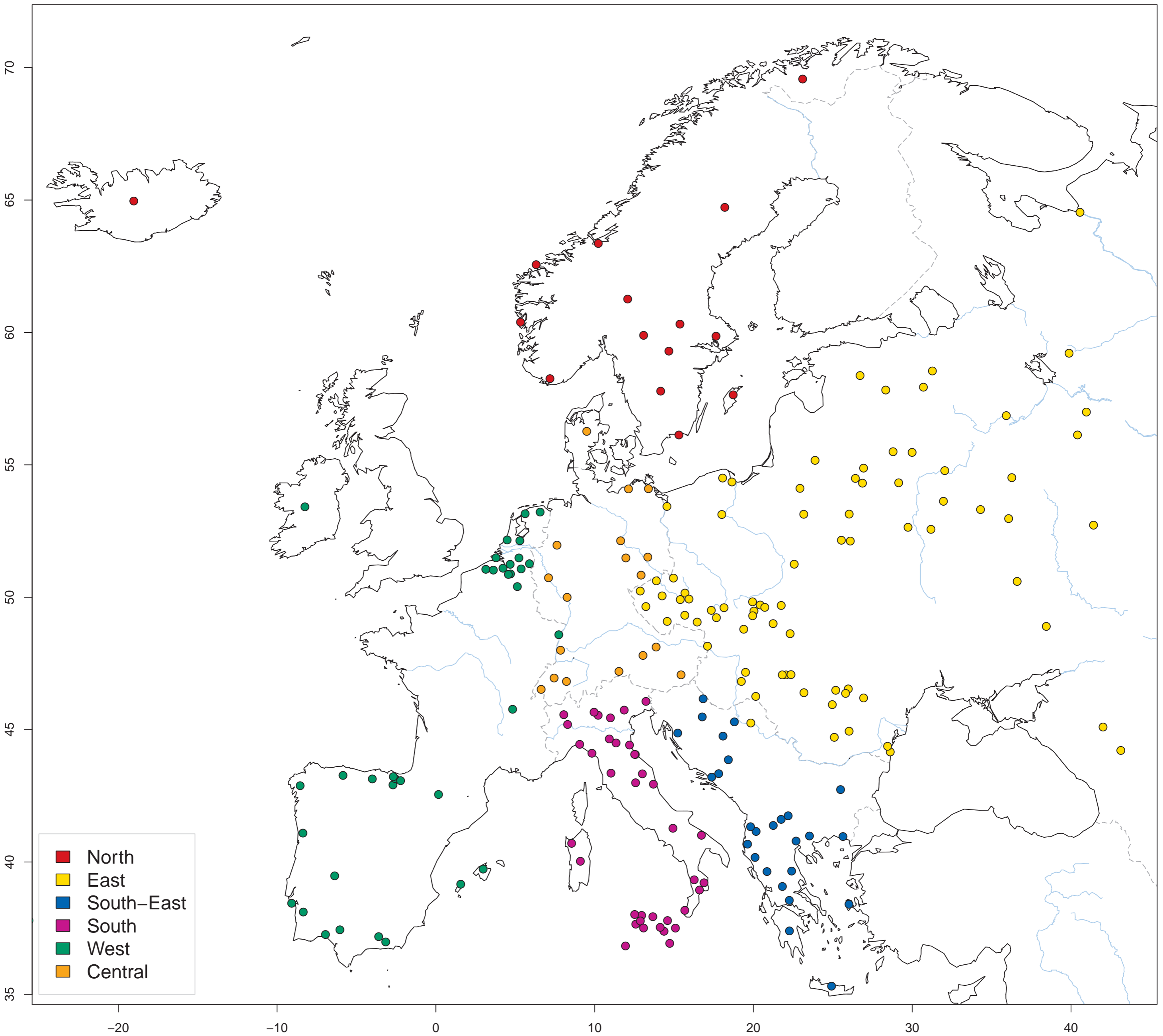

Supplement: Figure S8 — European sampling sites from the Y Chromosome Haplotype Reference Database (YHRD). For each European sampling site used in the comparative AMOVA analysis of European Y-chromosomal genetic diversity, its geographic location and its assignment to six broad geographic regions is depicted. (PDF) [file pgen.1003460.s008.pdf]

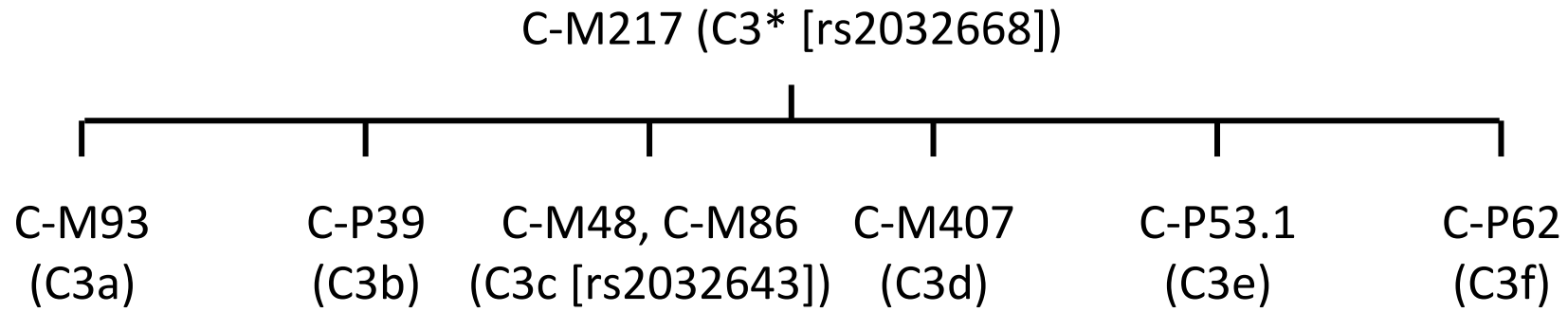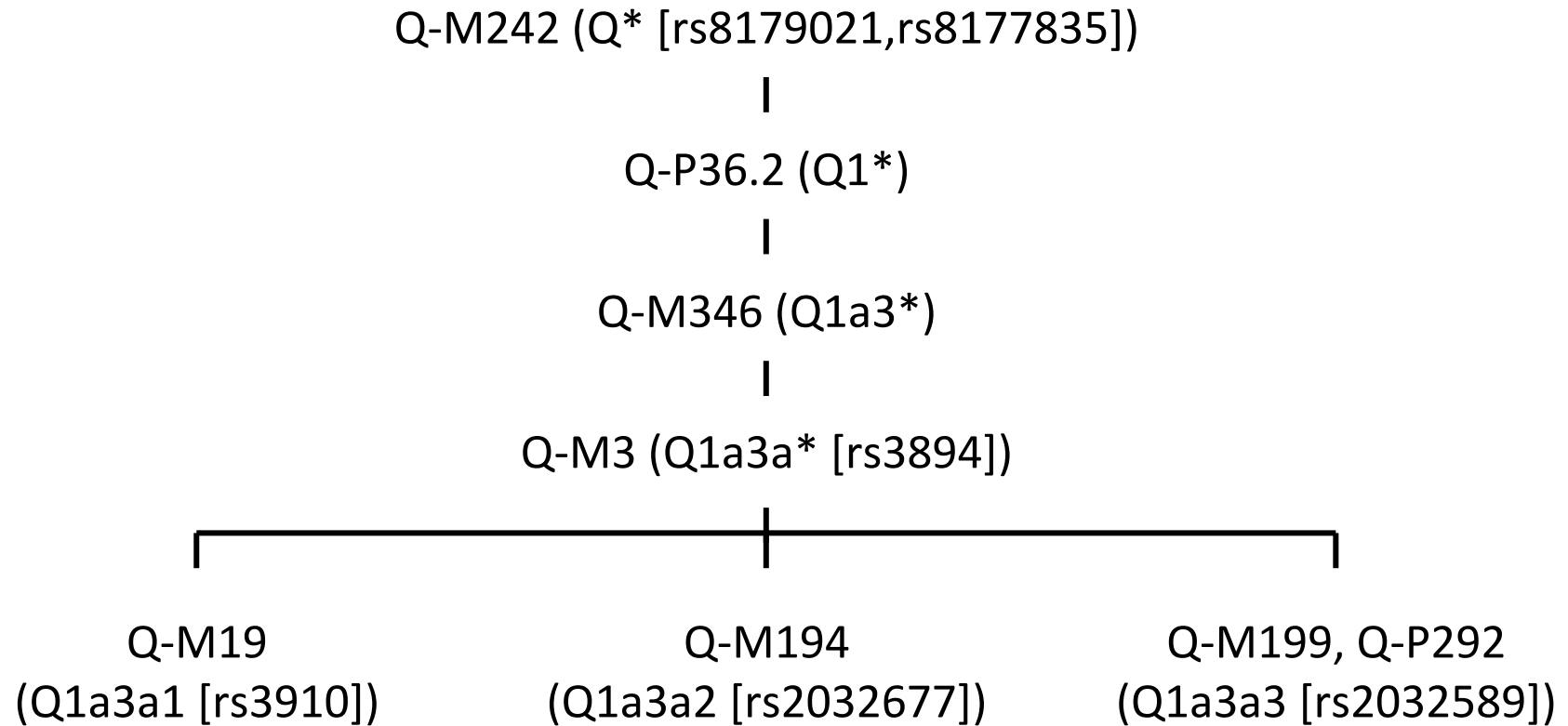

Supplement: Figure S9 — Y-SNP subgroups to C3* and Q*. Updated human Y-chromosomal haplogroup tree according to [65]. Where available, RefSeq numbers (according to NCBI dbSNP build37.3) of haplogroup-defining SNPs are given in parentheses. (PDF) [file pgen.1003460.s009.pdf]

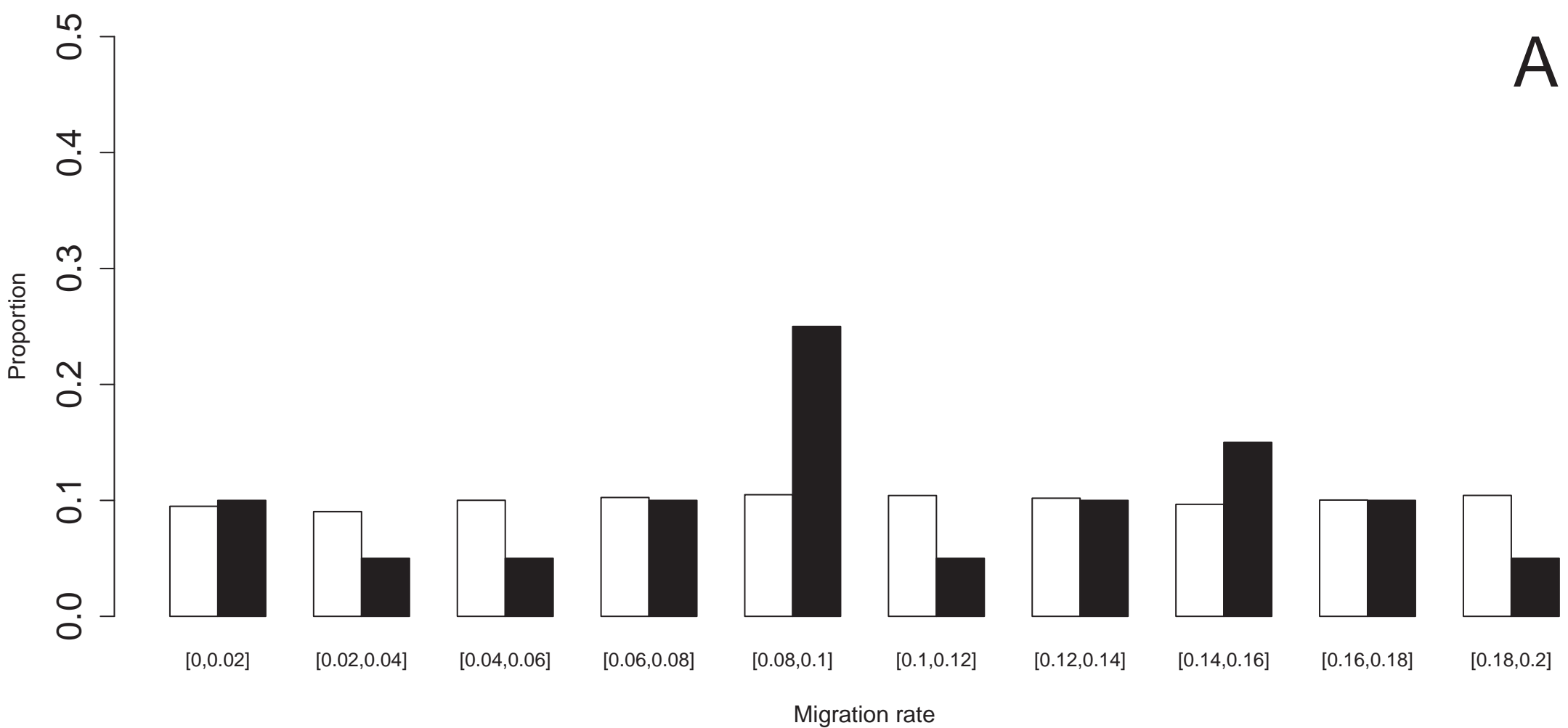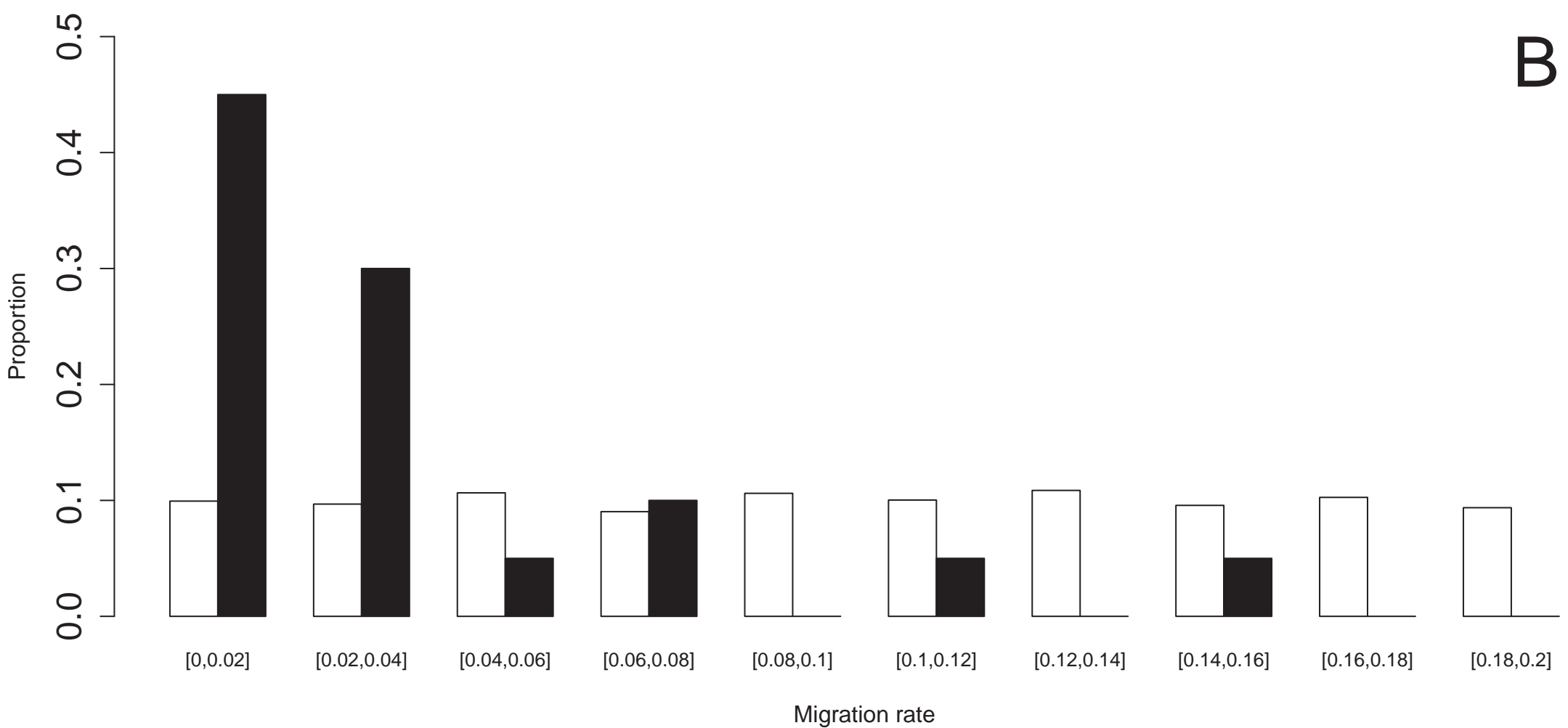

Supplement: Figure S10 — Migration rate distribution under scenario SA. Shown are the prior and ABC-derived posterior distributions of migration rates under scenario SA. In this scenario, all individuals of the two sampling sites with C3* carriers were merged into one population (SA/C+) and all remaining individuals were merged into a single second population (SA/C−; see Materials and Methods in the main text for details). A: migration rate into SA/C+; B: migration rate into SA/C−. White bars: prior distribution (obtained from 10,000 randomly selected simulated datasets); black bars: posterior distribution (obtained from those 100 simulated datasets that were closest to the original dataset with respect to the number of alleles and the gene diversity in a population. (PDF) [file pgen.1003460.s010.pdf]

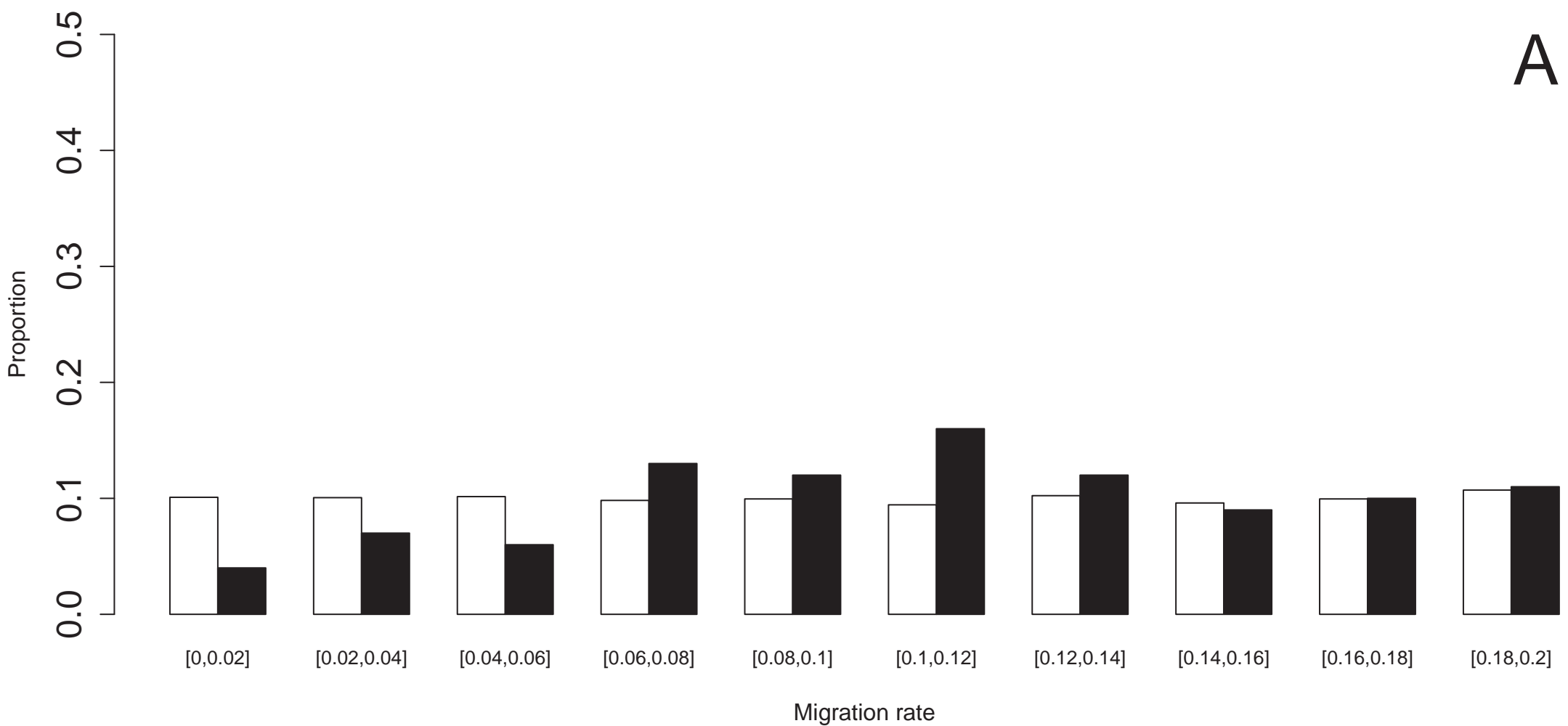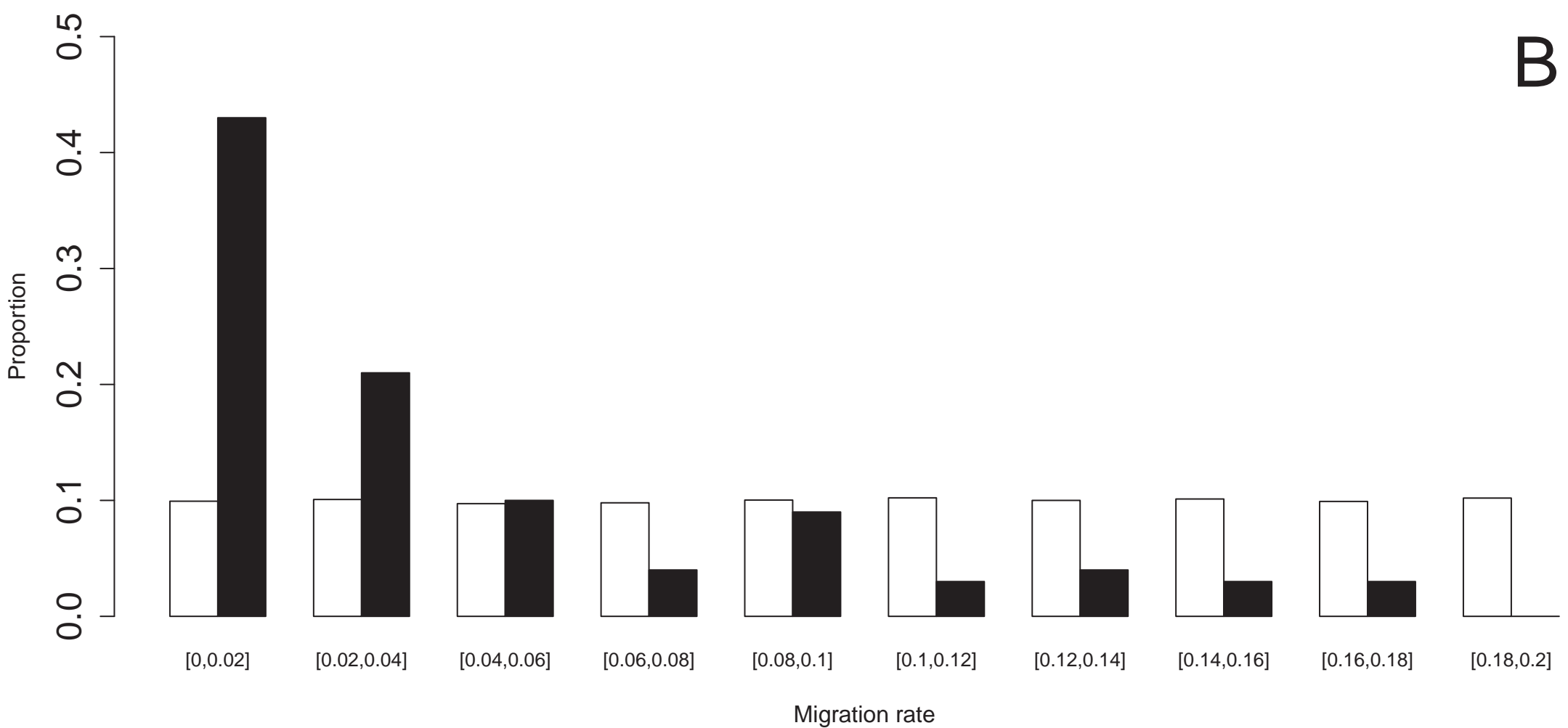

Supplement: Figure S11 — Migration rate distribution under scenario AA. Shown are the prior and ABC-derived posterior distributions of migration rates under scenario AA. In this scenario, all individuals of the two sampling sites with C3* carriers were merged into one population (SA/C+) and all remaining individuals were merged (SA/C−) together with all Q* carriers from two previous studies on North and Central American natives (NA/C−) into a single second population (see Materials and Methods in the main text for details). A: migration rate into SA/C+; B: migration rate into joint population of SA/C− and NA/C−. White bars: prior distribution (obtained from 10,000 randomly selected simulated datasets); black bars: posterior distribution (obtained from those 100 simulated datasets that were closest to the original dataset with respect to the number of alleles and the gene diversity in a population. (PDF) [file pgen.1003460.s011.pdf]

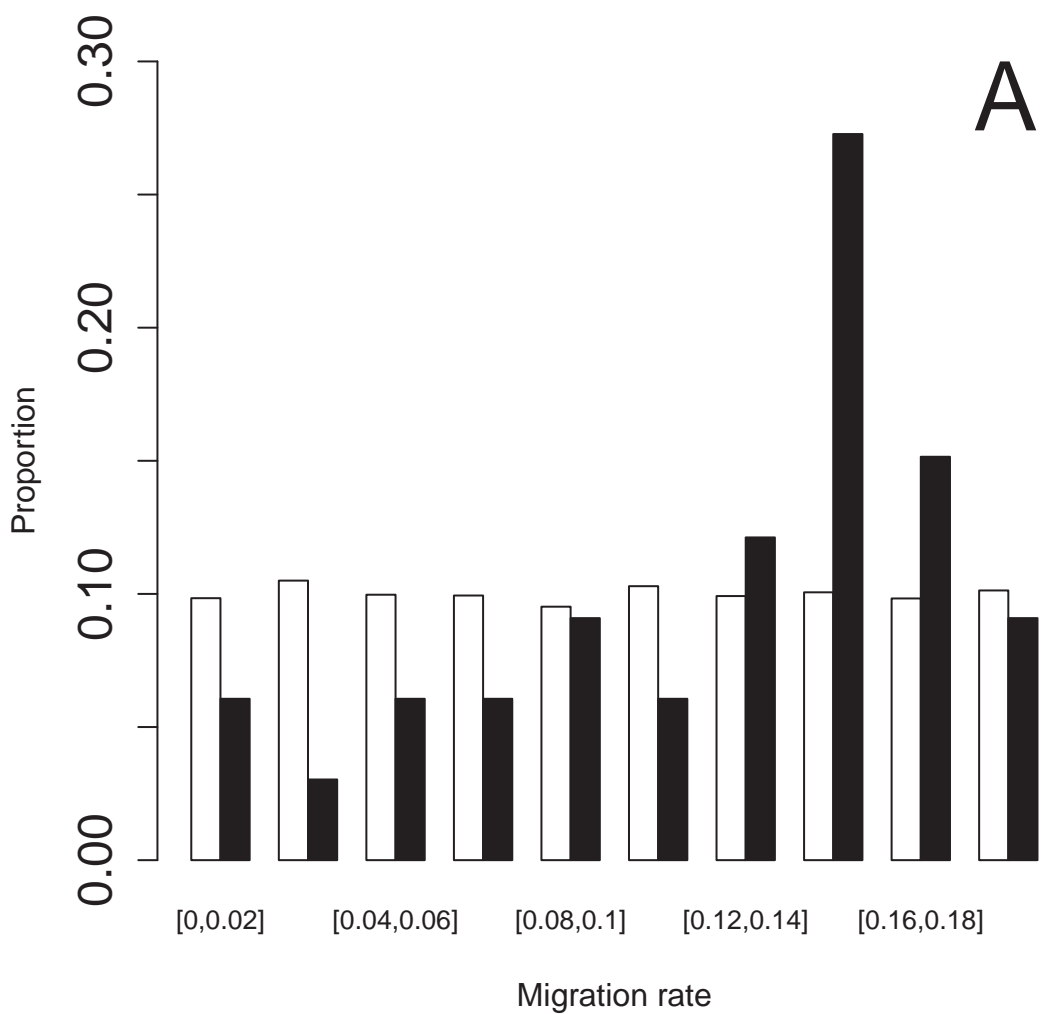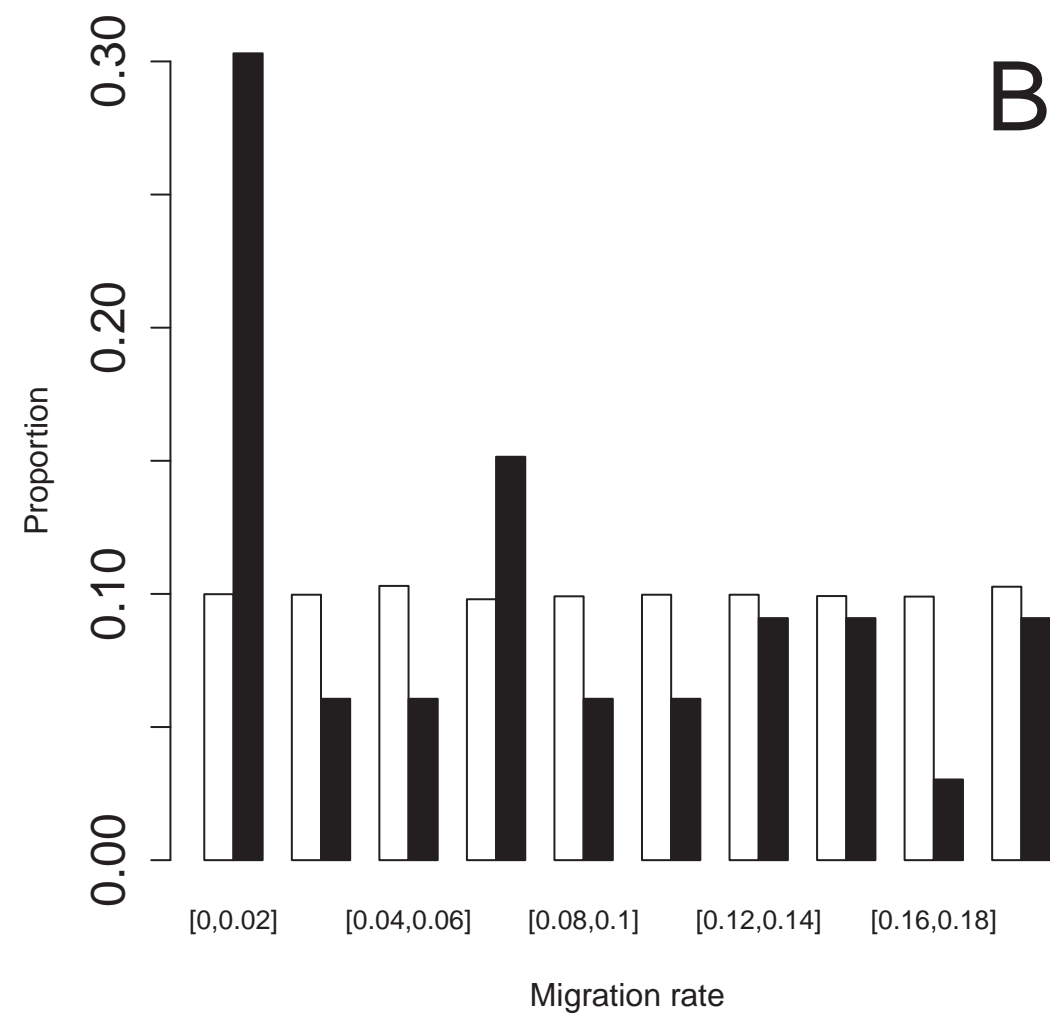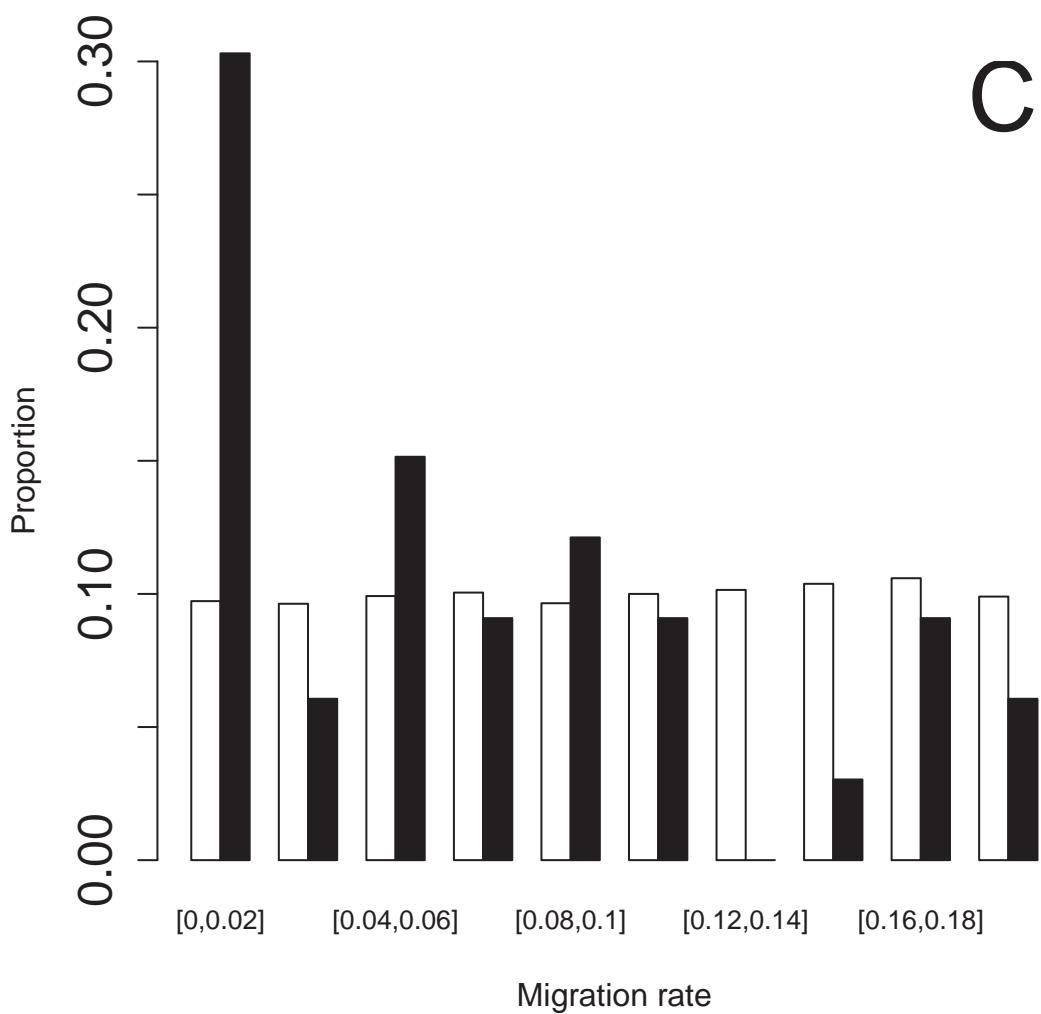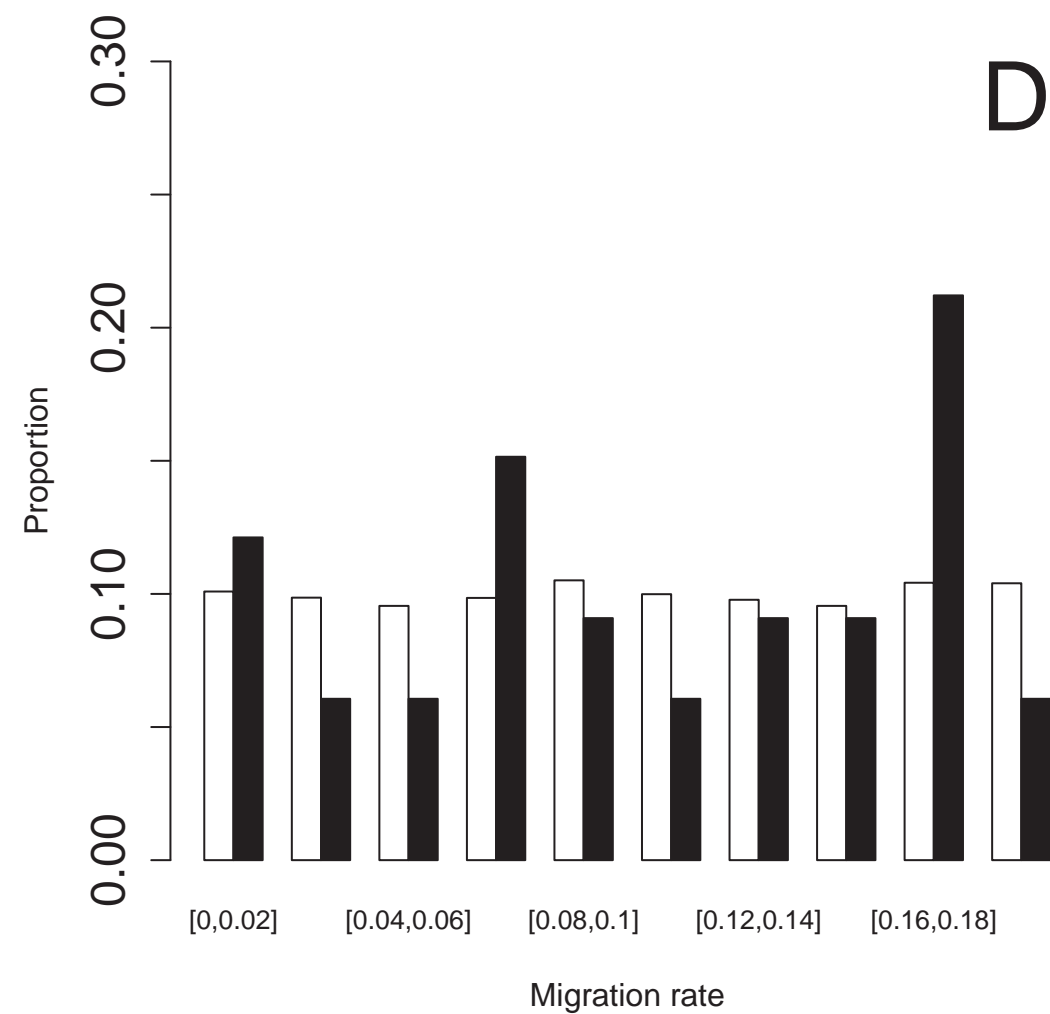

Supplement: Figure S12 — Migration rate distribution under scenario BA. Shown are the prior and ABC-derived posterior distributions of migration rates under scenario BA. In this scenario, all individuals of the two sampling sites with C3* carriers were merged into one population (SA/C+) and all remaining individuals from our study were merged into a single second population (SA/C−). All Q* carriers from two previous studies on North and Central American natives were combined in a third population (NA/C−; see Materials and Methods in the main text for details). A: migration rate into SA/C+; B: migration rate into SA/C−; C: migration rate into NA/C−; D: migration rate into the ancestral population of SA/C− and NA/C−. White bars: prior distribution (obtained from 10,000 randomly selected simulated datasets); black bars: posterior distribution (obtained from those 100 simulated datasets that were closest to the original dataset with respect to number of alleles and gene diversity in a population. (PDF) [file pgen.1003460.s012.pdf]

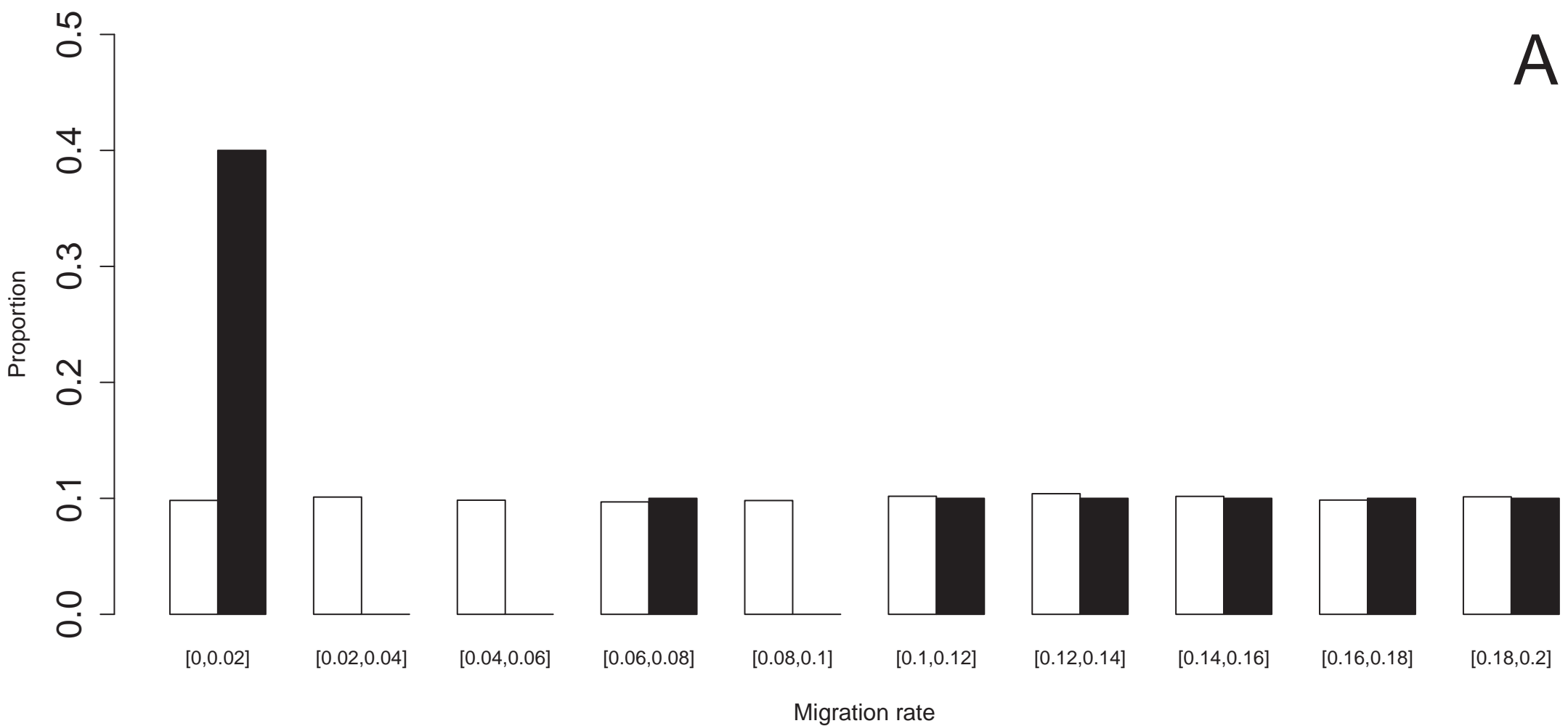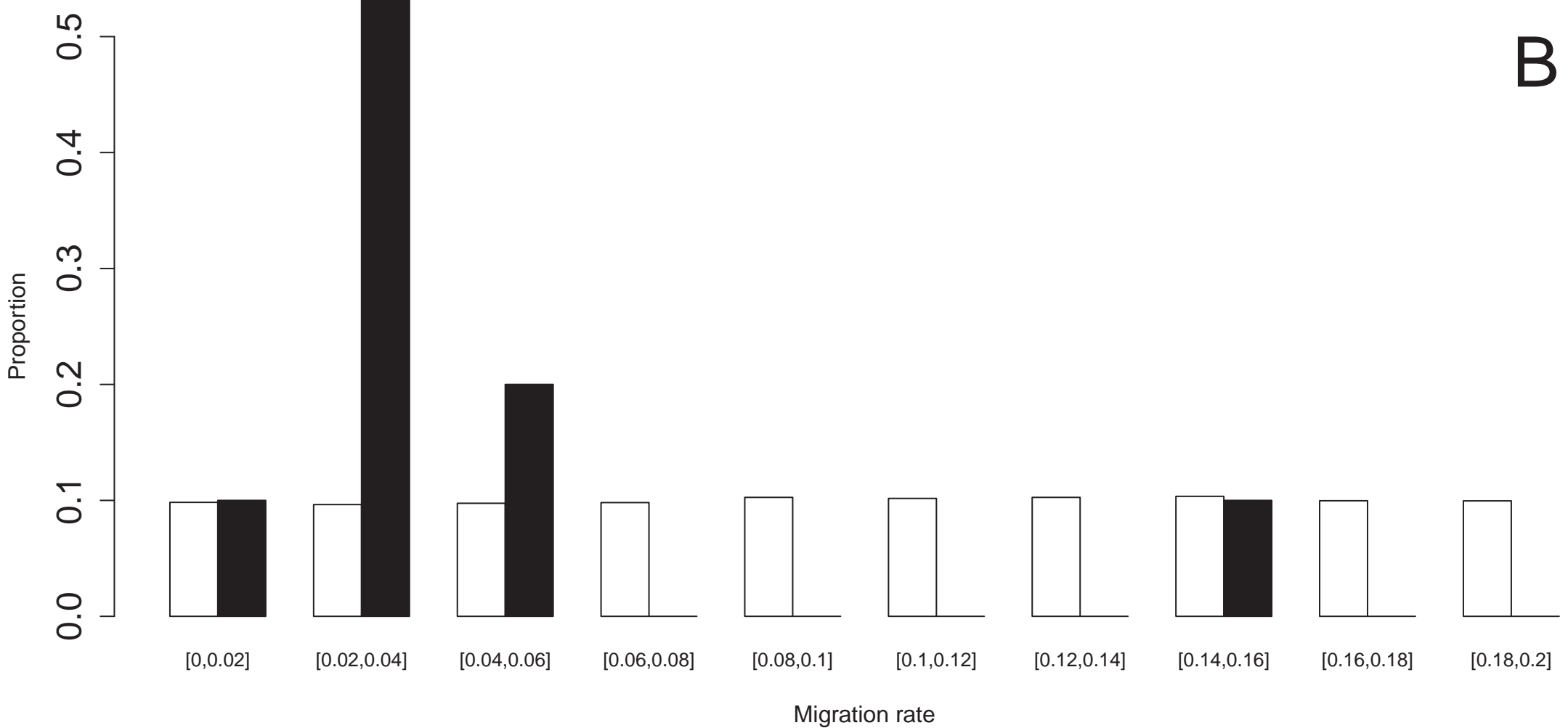

Supplement: Figure S13 — Migration rate distribution under scenario SA-10x. Shown are the prior and ABC-derived posterior distributions of migration rates under scenario SA. In this scenario, all individuals of the two sampling sites with C3* carriers were merged into one population (SA/C+) and all remaining individuals were merged into a single second population (SA/C−; see Materials and Methods in the main text for details). A: migration rate into SA/C+; B: migration rate into SA/C−. White bars: prior distribution (obtained from 10,000 randomly selected simulated datasets); black bars: posterior distribution (obtained from those 10 simulated datasets that were closest to the original dataset with respect to the number of alleles and the gene diversity in a population. (PDF) [file pgen.1003460.s013.pdf]

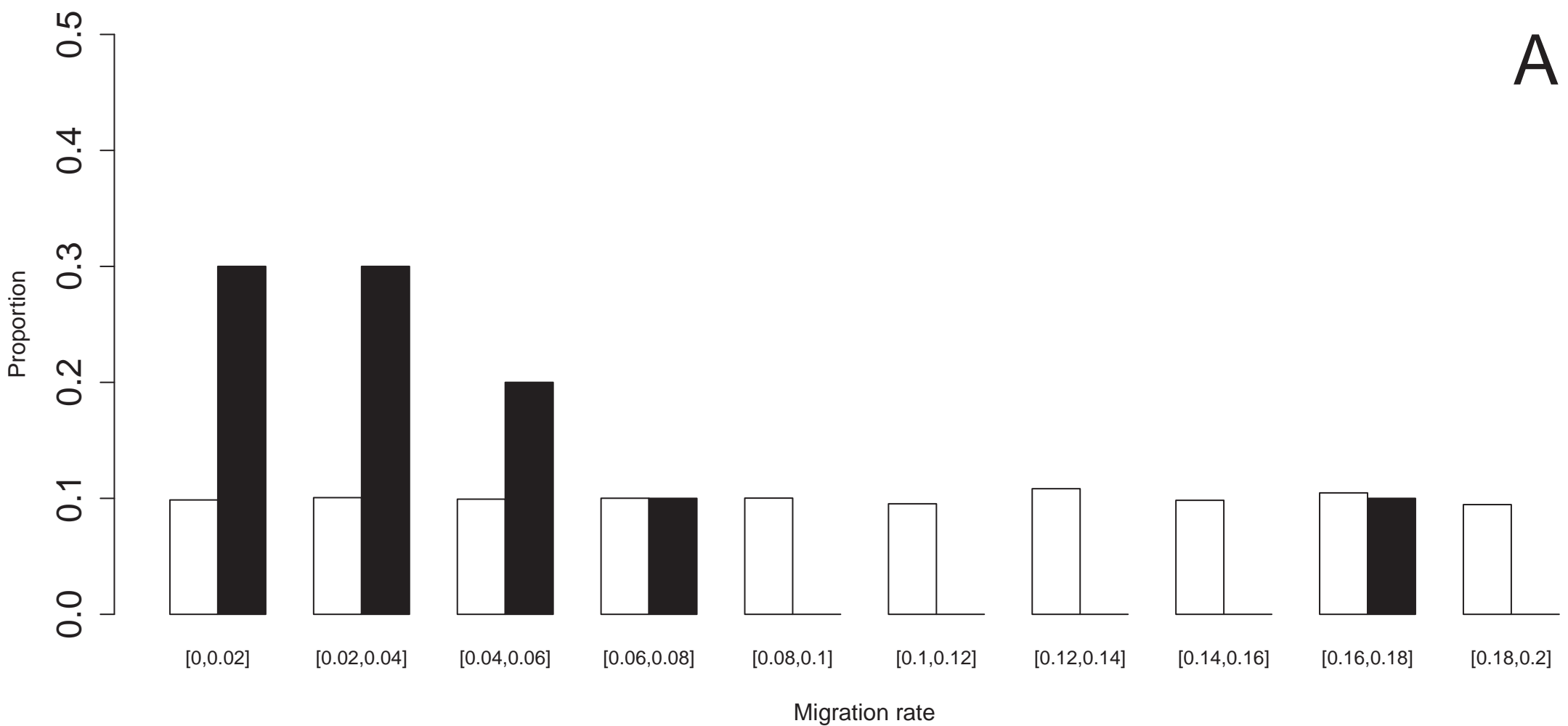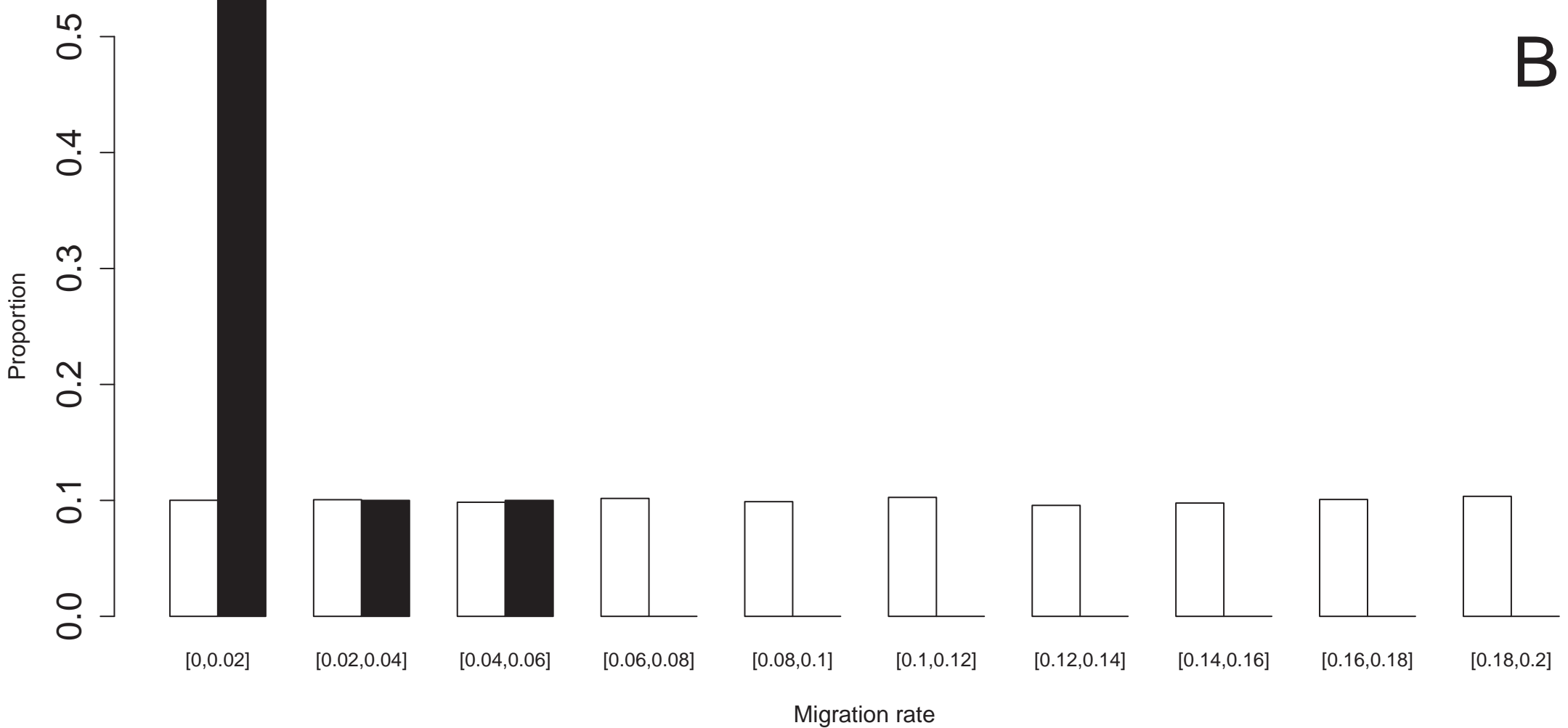

Supplement: Figure S14 — Migration rate distribution under scenario AA-10x. Shown are the prior and ABC-derived posterior distributions of migration rates under scenario AA. In this scenario, all individuals of the two sampling sites with C3* carriers were merged into one population (SA/C+) and all remaining individuals were merged (SA/C−) together with all Q* carriers from two previous studies on North and Central American natives (NA/C−) into a single second population (see Materials and Methods in the main text for details). A: migration rate into SA/C+; B: migration rate into joint population of SA/C− and NA/C−. White bars: prior distribution (obtained from 10,000 randomly selected simulated datasets); black bars: posterior distribution (obtained from those 10 simulated datasets that were closest to the original dataset with respect to the number of alleles and the gene diversity in a population. (PDF) [file pgen.1003460.s014.pdf]
