# Supplementary material for: Continent-Wide Decoupling of Y-Chromosomal Genetic Variation from Language and Geography in Native South Americans
Source: PLoS Genet. 2013 Apr 11;9(4):e1003460. doi: 10.1371/journal.pgen.1003460 (PMC3623769; doi:10.1371/journal.pgen.1003460)
Supplement: Table S1 — Sampling site characteristics. For each site, the sample size, geographic location, tribal and linguistic assignment and haplotype characteristics are given. (DOCX) [file pgen.1003460.s015.docx]

|  | **Geographic position** | | **Sampling site features** | | | | **Language** | | **Haplotypes** | | | **Geo-Cluster** | |
| --- | --- | --- | --- | --- | --- | --- | --- | --- | --- | --- | --- | --- | --- |
| **Site** | **Longi-tude** | **Lati-tude** | **Country** | **Province** | **Size** | **Ethnic group** | **Group** | **Language class** | **Haplogroup** | **Unique haplotypes** | **Haplotype diversity** | **A** | **B** |
| 1 | -76.83 | -1.37 | Ecuador | Pastaza | 57 | Kichwa(15), Shuar(2), Waorani(40) | Jivaroan(2), Quechua(15), Wao-Tiriro(40) | Andean(15), Equatorial-Tucanoan(2), Isolate(40) | C3*(4), Q1a3a(53) | 12 | 0.83 | 4 | 2 |
| 2 | -78.18 | -1.83 | Ecuador [Kichwa] |  | 27 | Kichwa(27) | Quechua(27) | Andean(27) | C3*(10), Q1a3(3), Q1a3a(14) | 14 | 0.95 | 4 | 2 |
| 3 | -75.20 | -12.08 | Perú | Junín | 13 | Huanca(13) | Quechua(13) | Andean(13) | Q1a3a(13) | 10 | 0.95 | 4 | 1 |
| 4 | -65.55 | -12.25 | Bolivia | El Beni | 7 | Ignaciano(1), Mojeño(2), Movima(1), Trinitario(3) | Arawakan-Maipuran(6), Movima(1) | Equatorial-Tucanoan(7) | Q1a3(2), Q1a3a(5) | 5 | 0.97 | 3 | 2 |
| 5 | -65.57 | -12.99 | Bolivia | El Beni | 1 | Trinitario(1) | Arawakan-Maipuran(1) | Equatorial-Tucanoan(1) | Q1a3(1) | 1 | 0.00 | 3 | 2 |
| 6 | -63.78 | -13.18 | Bolivia | El Beni | 1 | Aymara(1) | Aymara(1) | Andean(1) | Q1a3(1) | 1 | 0.00 | 3 | 2 |
| 7 | -71.98 | -13.52 | Perú | Cuzco | 10 | Chumbivilca(10) | Quechua(10) | Andean(10) | Q1a3(1), Q1a3a(9) | 7 | 0.91 | 4 | 1 |
| 8 | -66.15 | -13.76 | Bolivia | El Beni | 6 | Ignaciano(4), Mojeño(1), Yuracare(1) | Arawakan-Maipuran(5), Yuracare(1) | Equatorial-Tucanoan(6) | Q1a3(2), Q1a3a(4) | 6 | 1.00 | 3 | 2 |
| 9 | -66.87 | -14.17 | Bolivia | El Beni | 2 | Mojeño(1), Trinitario(1) | Arawakan-Maipuran(2) | Equatorial-Tucanoan(2) | Q1a3a(2) | 2 | 1.00 | 3 | 2 |
| 10 | -67.08 | -14.22 | Bolivia | ElBeni | 9 | Trinitario(8), Yuracare(1) | Arawakan-Maipuran(8), Yuracare(1) | Equatorial-Tucanoan(9) | Q1a3(2), Q1a3a(7) | 8 | 0.99 | 3 | 2 |
| 11 | -66.11 | -14.44 | Bolivia | El Beni | 1 | Yuracare(1) | Yuracare(1) | Equatorial-Tucanoan(1) | Q1a3a(1) | 1 | 0.00 | 3 | 2 |
| 12 | -66.19 | -14.52 | Bolivia | El Beni | 2 | Aymara(1), Borjano(1) | Aymara(1), Pano-Tacana(1) | Andean(1), Ge-Pano-Carib(1) | Q1a3a(2) | 2 | 1.00 | 3 | 2 |
| 13 | -66.16 | -14.53 | Bolivia | El Beni | 2 | Chimane(2) | Moseten(2) | Ge-Pano-Carib(2) | Q1a3(2) | 1 | 0.00 | 3 | 2 |
| 14 | -65.26 | -14.54 | Bolivia | El Beni | 4 | Trinitario(4) | Arawakan-Maipuran(4) | Equatorial-Tucanoan(4) | Q1a3(1), Q1a3a(3) | 4 | 1.00 | 3 | 2 |
| 15 | -66.39 | -14.54 | Bolivia | El Beni | 1 | Trinitario(1) | Arawakan-Maipuran(1) | Equatorial-Tucanoan(1) | Q1a3(1) | 1 | 0.00 | 3 | 2 |
| 16 | -65.30 | -14.56 | Bolivia | El Beni | 4 | Mojeño(3), Trinitario(1) | Arawakan-Maipuran(4) | Equatorial-Tucanoan(4) | Q1a3(2), Q1a3a(2) | 2 | 1.00 | 3 | 2 |
| 17 | -66.99 | -14.57 | Bolivia | El Beni | 4 | Mojeño(1), Trinitario(2), Yuracare(1) | Arawakan-Maipuran(3), Yuracare(1) | Equatorial-Tucanoan(4) | Q1a3a(4) | 3 | 0.94 | 3 | 2 |
| 18 | -64.67 | -14.95 | Bolivia | El Beni | 1 | Mojeño(1) | Arawakan-Maipuran(1) | Equatorial-Tucanoan(1) | Q1a3a(1) | 1 | 0.00 | 3 | 2 |
| 19 | -65.27 | -15.01 | Bolivia | El Beni | 1 | Ignaciano(1) | Arawakan-Maipuran(1) | Equatorial-Tucanoan(1) | Q1a3a(1) | 1 | 0.00 | 3 | 2 |
| 20 | -65.89 | -15.03 | Bolivia | El Beni | 17 | Mojeño(1), Trinitario(15), Yuracare(1) | Arawakan-Maipuran(16), Yuracare(1) | Equatorial-Tucanoan(17) | Q1a3(1), Q1a3a(16) | 11 | 0.98 | 3 | 2 |
| 21 | -65.12 | -15.05 | Bolivia | El Beni | 2 | Yuracare(2) | Yuracare(2) | Equatorial-Tucanoan(2) | Q1a3a(2) | 1 | 0.00 | 3 | 2 |
| 22 | -66.08 | -15.50 | Bolivia | El Beni | 8 | Chimane(8) | Moseten(8) | Ge-Pano-Carib(8) | Q1a3(7), Q1a3a(1) | 2 | 0.44 | 3 | 2 |
| 23 | -70.02 | -15.84 | Perú | Puno | 3 | Puno(3) | Aymara(3) | Andean(3) | Q1a3a(3) | 3 | 1.00 | 4 | 1 |
| 24 | -71.53 | -16.38 | Perú | Arequipa | 16 | Chuquibamba(16) | Quechua(16) | Andean(16) | Q1a3a(16) | 11 | 0.94 | 4 | 1 |
| 25 | -46.17 | -2.80 | Brazil | Maranhão | 27 | Urubu-Kaapor(27) | Tupi(27) | Equatorial-Tucanoan(27) | Q1a3a(27) | 5 | 0.41 | 6 | 2 |
| 26 | -54.78 | -20.77 | Brazil | Mato Grosso do Sul | 32 | Terena(32) | Arawakan(32) | Equatorial-Tucanoan(32) | Q1a3a(32) | 8 | 0.83 | 2 | 3 |
| 27 | -62.71 | -22.27 | Argentina | Salta | 8 | Wichi(8) | Mataco-Guaicuruan(8) | Ge-Pano-Carib(8) | Q1a3a(8) | 5 | 0.86 | 2 | 3 |
| 28 | -63.93 | -22.77 | Argentina | Formosa | 14 | Wichi(14) | Mataco-Guaicuruan(14) | Ge-Pano-Carib(14) | Q1a3a(14) | 5 | 0.88 | 2 | 3 |
| 29 | -61.85 | -23.90 | Argentina | Formosa | 23 | Wichi(23) | Mataco-Guaicuruan(23) | Ge-Pano-Carib(23) | Q1a3a(23) | 8 | 0.82 | 2 | 3 |
| 30 | -60.60 | -24.70 | Argentina | Formosa | 12 | Pilaga-Labomba(12) | Mataco-Guaicuruan(12) | Ge-Pano-Carib(12) | Q1a3a(12) | 7 | 0.97 | 2 | 3 |
| 31 | -58.24 | -25.13 | Argentina | Formosa | 33 | Toba(33) | Mataco-Guaicuruan(33) | Ge-Pano-Carib(33) | Q1a3a(33) | 9 | 0.89 | 2 | 3 |
| 32 | -59.86 | -25.21 | Argentina | Formosa | 53 | Pilaga(53) | Mataco-Guaicuruan(53) | Ge-Pano-Carib(53) | Q1a3a(53) | 16 | 0.94 | 2 | 3 |
| 33 | -60.43 | -25.40 | Argentina | Chaco | 40 | Toba(40) | Mataco-Guaicuruan(40) | Ge-Pano-Carib(40) | Q1a3a(34), Q1a3a1(6) | 19 | 0.96 | 2 | 3 |
| 34 | -54.58 | -25.59 | Argentina | Misiones | 10 | Guarani(10) | Mbya-Guarani(10) | Equatorial-Tucanoan(10) | Q1a3a(10) | 5 | 0.85 | 2 | 3 |
| 35 | -54.58 | -25.60 | Argentina | Misiones | 42 | Guarani(42) | Mbya-Guarani(42) | Equatorial-Tucanoan(42) | Q1a3a(42) | 11 | 0.85 | 2 | 3 |
| 36 | -65.73 | -26.40 | Argentina | Tucumán | 14 | Colla(14) | Quechua(14) | Andean(14) | Q1a3a(14) | 7 | 0.98 | 2 | 1 |
| 37 | -59.35 | -26.55 | Argentina | Chaco | 17 | Toba(17) | Mataco-Guaicuruan(17) | Ge-Pano-Carib(17) | Q1a3(1), Q1a3a(16) | 8 | 0.88 | 2 | 3 |
| 38 | -55.19 | -26.96 | Argentina | Misiones | 8 | Guarani(8) | Mbya-Guarani(8) | Equatorial-Tucanoan(8) | Q1a3a(8) | 2 | 0.44 | 2 | 3 |
| 39 | -54.35 | -26.99 | Argentina | Misiones | 13 | Guarani(13) | Mbya-Guarani(13) | Equatorial-Tucanoan(13) | Q1a3a(13) | 10 | 0.97 | 2 | 3 |
| 40 | -55.07 | -27.12 | Argentina | Misiones | 11 | Guarani(11) | Mbya-Guarani(11) | Equatorial-Tucanoan(11) | Q1a3a(11) | 6 | 0.91 | 2 | 3 |
| 41 | -46.67 | -3.50 | Brazil | Maranhão | 46 | Awa-Guajá(46) | TupiGuaraní(46) | Equatorial-Tucanoan(46) | Q1a3a(46) | 5 | 0.75 | 6 | 2 |
| 42 | -73.24 | -3.73 | Perú | Loreto | 5 | Iquito(5) | Záparo(5) | Andean(5) | Q1a3a(5) | 5 | 1.00 | 4 | 2 |
| 43 | -53.46 | -3.95 | Brazil | Pará | 5 | Mapuera(5) | HixcaryanaCarib(5) | Ge-Pano-Carib(5) | Q1a3a(5) | 4 | 0.96 | 6 | 2 |
| 44 | -67.98 | -38.93 | Argentina | Rio Negro | 3 | Mapuche(3) | Araucanian-Mapudungun(3) | Andean(3) | Q1a3a(3) | 3 | 1.00 | 1 | 1 |
| 45 | -67.57 | -39.03 | Argentina | Rio Negro | 3 | Mapuche(3) | Araucanian-Mapudungun(3) | Andean(3) | Q1a3a(3) | 3 | 1.00 | 1 | 1 |
| 46 | -68.59 | -39.71 | Argentina | Rio Negro | 5 | Mapuche(5) | Araucanian-Mapudungun(5) | Andean(5) | Q1a3a(5) | 5 | 1.00 | 1 | 1 |
| 47 | -68.87 | -39.98 | Argentina | Rio Negro | 5 | Mapuche(5) | Araucanian-Mapudungun(5) | Andean(5) | Q1a3a(5) | 5 | 1.00 | 1 | 1 |
| 48 | -49.67 | -4.20 | Brazil | Pará | 15 | Asurini(15) | TupiGuaraní(15) | Equatorial-Tucanoan(15) | Q1a3a(15) | 5 | 0.88 | 6 | 2 |
| 49 | -54.17 | -4.33 | Brazil | Pará | 20 | Arara(20) | Carib(20) | Ge-Pano-Carib(20) | Q1a3(7), Q1a3a(13) | 3 | 0.88 | 6 | 2 |
| 50 | -69.08 | -40.11 | Argentina | Rio Negro | 1 | Mapuche(1) | Araucanian-Mapudungun(1) | Andean(1) | Q1a3a(1) | 1 | 0.00 | 1 | 1 |
| 51 | -62.99 | -40.81 | Argentina | Rio Negro | 5 | Mapuche(5) | Araucanian-Mapudungun(5) | Andean(5) | Q1a3a(5) | 3 | 0.84 | 1 | 1 |
| 52 | -71.30 | -41.15 | Argentina | Rio Negro | 13 | Mapuche(13) | Araucanian-Mapudungun(13) | Andean(13) | Q1a3a(13) | 8 | 0.95 | 1 | 1 |
| 53 | -69.55 | -41.33 | Argentina | Rio Negro | 2 | Mapuche(2) | Araucanian-Mapudungun(2) | Andean(2) | Q1a3a(2) | 2 | 1.00 | 1 | 1 |
| 54 | -71.51 | -41.97 | Argentina | Rio Negro | 2 | Mapuche(2) | Araucanian-Mapudungun(2) | Andean(2) | Q1a3a(2) | 2 | 1.00 | 1 | 1 |
| 55 | -71.31 | -42.91 | Argentina | Chubut | 7 | Mapuche(7) | Araucanian-Mapudungun(7) | Andean(7) | Q1a3a(7) | 7 | 1.00 | 1 | 1 |
| 56 | -65.31 | -43.25 | Argentina | Chubut | 3 | Mapuche(3) | Araucanian-Mapudungun(3) | Andean(3) | Q1a3a(3) | 3 | 1.00 | 1 | 1 |
| 57 | -70.97 | -46.08 | Argentina | Chubut | 10 | Tehuelche(10) | Chon(10) | Andean(10) | Q1a3a(10) | 9 | 0.99 | 1 | 1 |
| 58 | -51.87 | -5.15 | Brazil | Pará | 21 | Ipixuna(21) | TupiGuaraní(21) | Equatorial-Tucanoan(21) | Q1a3a(21) | 2 | 0.18 | 6 | 2 |
| 59 | -51.87 | -5.75 | Brazil | Pará | 38 | Parakana(38) | TupiGuaraní(38) | Equatorial-Tucanoan(38) | Q1a3a(38) | 4 | 0.26 | 6 | 2 |
| 60 | -51.00 | -5.92 | Brazil | Pará | 13 | Kayapó-Xikrin(13) | Ge(13) | Ge-Pano-Carib(13) | Q1a3a(13) | 6 | 0.80 | 6 | 2 |
| 61 | -49.92 | -7.53 | Brazil | Pará | 18 | Gaviao(18) | Ge(18) | Ge-Pano-Carib(18) | Q1a3a(12), Q1a3a - del(6) | 5 | 0.94 | 6 | 2 |
| 62 | -74.82 | -8.48 | Perú | Ucayali | 21 | Shipibo-Conibo(21) | Shipibo(21) | Ge-Pano-Carib(21) | Q1a3a(21) | 10 | 0.86 | 4 | 2 |
| 63 | -54.00 | -8.67 | Brazil | Pará | 4 | Kayapó-Kararao(4) | Ge(4) | Ge-Pano-Carib(4) | Q1a3a(4) | 1 | 0.00 | 6 | 2 |
| 64 | -64.25 | -9.50 | Brazil | Rondonia | 17 | Karitiana(17) | TupiArikem(17) | Equatorial-Tucanoan(17) | Q1a3a(17) | 5 | 0.67 | 3 | 2 |
| 65 | -55.50 | 0.17 | Brazil | Pará | 25 | Zoé(25) | TupiGuaraní(25) | Equatorial-Tucanoan(25) | Q1a3a(25) | 2 | 0.15 | 5 | 2 |
| 66 | -77.63 | 0.83 | Colombia | Nariño | 1 | Pastos(1) | Chibcha(1) | Chibchan-Paezan(1) | Q1a3a(1) | 1 | 0.00 | 4 | 2 |
| 67 | -76.83 | 1.68 | Colombia | Cauca | 1 | Nasa(1) | Chibcha(1) | Chibchan-Paezan(1) | Q1a3a(1) | 1 | 0.00 | 4 | 2 |
| 68 | -76.73 | 1.85 | Colombia | Cauca | 1 | Yanacona(1) | Quechua(1) | Andean(1) | Q1a3a(1) | 1 | 0.00 | 4 | 2 |
| 69 | -52.75 | 1.95 | Brazil | Amapá | 13 | Waiapy(13) | TupiGuaraní(13) | Equatorial-Tucanoan(13) | Q1a3a(13) | 1 | 0.00 | 5 | 2 |
| 70 | -55.82 | 1.98 | Brazil | Amapá | 35 | Tiriyo(35) | Carib(35) | Ge-Pano-Carib(35) | Q1a3(2), Q1a3a(33) | 16 | 0.92 | 5 | 2 |
| 71 | -71.33 | 12.07 | Venezuela | Guajira | 19 | Wayuu(19) | Arawakan(19) | Equatorial-Tcanoan(19) | Q1a3a(19) | 16 | 0.99 | 4 | 2 |
| 72 | -76.42 | 2.25 | Colombia | Cauca | 7 | Coconuco(7) | Guambiano(7) | Chibchan-Paezan(7) | Q1a3a(7) | 4 | 0.93 | 4 | 2 |
| 73 | -76.58 | 2.25 | Colombia | Cauca | 1 | Yanacona(1) | Quechua(1) | Andean(1) | Q1a3a(1) | 1 | 0.00 | 4 | 2 |
| 74 | -62.30 | 2.40 | Brazil | Roraima | 10 | Yanomami(10) | Yanomam(10) | Chibchan-Paezan(10) | Q1a3(9), Q1a3a(1) | 6 | 0.84 | 5 | 2 |
| 75 | -76.37 | 2.60 | Colombia | Cauca | 15 | Guambiano(15) | Guambiano(15) | Chibchan-Paezan(15) | Q1a3(9), Q1a3a(6) | 8 | 0.89 | 4 | 2 |
| 76 | -76.52 | 2.64 | Colombia | Cauca | 1 | Guambiano(1) | Guambiano(1) | Chibchan-Paezan(1) | Q1a3a(1) | 1 | 0.00 | 4 | 2 |
| 77 | -75.97 | 2.67 | Colombia | Cauca | 2 | Nasa(2) | Chibcha(2) | Chibchan-Paezan(2) | Q1a3a(2) | 2 | 1.00 | 4 | 2 |
| 78 | -76.31 | 2.78 | Colombia | Cauca | 1 | Nasa(1) | Chibcha(1) | Chibchan-Paezan(1) | Q1a3a(1) | 1 | 0.00 | 4 | 2 |
| 79 | -77.07 | 5.33 | Colombia | Chocó | 1 | Embera(1) | Emberá(1) | Chibchan-Paezan(1) | Q1a3a(1) | 1 | 0.00 | 4 | 2 |
| 80 | -74.70 | 7.08 | Colombia | Antioquia | 24 | Embera-Chamí(24) | Emberá(24) | Chibchan-Paezan(24) | Q1a3(4), Q1a3a(20) | 9 | 0.95 | 4 | 2 |
| 81 | -72.66 | 9.39 | Venezuela | Zulia | 16 | BariBoxi(16) | Chibcha(16) | Chibchan-Paezan(16) | Q1a3a(16) | 5 | 0.87 | 4 | 2 |
