# Supplementary material for: Continent-Wide Decoupling of Y-Chromosomal Genetic Variation from Language and Geography in Native South Americans
Source: PLoS Genet. 2013 Apr 11;9(4):e1003460. doi: 10.1371/journal.pgen.1003460 (PMC3623769; doi:10.1371/journal.pgen.1003460)
Supplement: Table S2 — Mutation rate estimates for Y-STRs studied. For each marker, the average mutation rate used in this study in this study is given according to http://www.yhrd.org/Research/Loci (release 42, January 11, 2013). (DOCX) [file pgen.1003460.s016.docx]

| **Marker** | **Mutation rate** (×10^-3^) | **Weight in median-joining network analysis** |
| --- | --- | --- |
| DYS19 | 2.3 | 4 |
| DYS389I | 2.5 | 4 |
| DYS389II | 3.6 | 3 |
| DYS390 | 2.1 | 5 |
| DYS391 | 2.6 | 4 |
| DYS392 | 0.4 | 25 |
| DYS393 | 1.0 | 10 |
| DYS437 | 1.2 | 8 |
| DYS438 | 0.3 | 33 |
| DYS439 | 5.2 | 2 |
| DYS448 | 1.5 | 7 |
| DYS456 | 4.2 | 2 |
| DYS458 | 6.4 | 2 |
| DYS635 | 3.5 | 3 |
| GATA H4 | 2.4 | 4 |
