# Supplementary material for: Continent-Wide Decoupling of Y-Chromosomal Genetic Variation from Language and Geography in Native South Americans
Source: PLoS Genet. 2013 Apr 11;9(4):e1003460. doi: 10.1371/journal.pgen.1003460 (PMC3623769; doi:10.1371/journal.pgen.1003460)
Supplement: Table S3 — Y-SNP haplogroup and language group. Cross-tabulation of haplogroup of carried haplotype vs group of spoken language per individual. Results are based on all 1011 samples. (DOCX) [file pgen.1003460.s017.docx]

| **Language group** | **Haplogroup** | | | | |
| --- | --- | --- | --- | --- | --- |
|  | C3* | Q1a3 | Q1a3a | Q1a3a - del | Q1a3a1 |
| Araucanian-Mapudungun | 0 | 0 | 49 | 0 | 0 |
| Arawakan | 0 | 0 | 51 | 0 | 0 |
| Arawakan-Maipuran | 0 | 11 | 41 | 0 | 0 |
| Aymara | 0 | 1 | 4 | 0 | 0 |
| Carib | 0 | 9 | 46 | 0 | 0 |
| Chibcha | 0 | 0 | 21 | 0 | 0 |
| Chon | 0 | 0 | 10 | 0 | 0 |
| Emberá | 0 | 4 | 21 | 0 | 0 |
| Ge | 0 | 0 | 29 | 6 | 0 |
| Guambiano | 0 | 9 | 14 | 0 | 0 |
| Hixcaryana Carib | 0 | 0 | 5 | 0 | 0 |
| Jivaroan | 0 | 0 | 2 | 0 | 0 |
| Mataco-Guaicuruan | 0 | 1 | 193 | 0 | 6 |
| Mbya-Guarani | 0 | 0 | 84 | 0 | 0 |
| Moseten | 0 | 9 | 1 | 0 | 0 |
| Movima | 0 | 0 | 1 | 0 | 0 |
| Pano-Tacana | 0 | 0 | 1 | 0 | 0 |
| Quechua | 11 | 4 | 82 | 0 | 0 |
| Shipibo | 0 | 0 | 21 | 0 | 0 |
| Tupi | 0 | 0 | 27 | 0 | 0 |
| Tupi Arikem | 0 | 0 | 17 | 0 | 0 |
| Tupi Guaraní | 0 | 0 | 158 | 0 | 0 |
| Wao Tiriro | 3 | 0 | 37 | 0 | 0 |
| Yanomam | 0 | 9 | 1 | 0 | 0 |
| Yuracare | 0 | 1 | 6 | 0 | 0 |
| Záparo | 0 | 0 | 5 | 0 | 0 |
