# Supplementary material for: Continent-Wide Decoupling of Y-Chromosomal Genetic Variation from Language and Geography in Native South Americans
Source: PLoS Genet. 2013 Apr 11;9(4):e1003460. doi: 10.1371/journal.pgen.1003460 (PMC3623769; doi:10.1371/journal.pgen.1003460)
Supplement: Table S4 — European sampling sites for comparative analysis. For each sampling site that has been used in the comparative analysis with South-America, the country and region of origin as well as its geographic position, its regional assignment following [7] and the sample size are given. Sites from cities with more than 500,000 inhabitants were not included in the analysis. (DOCX) [file pgen.1003460.s018.docx]

| **Site** | **Country** | **Region** | **Longitude** | **Latitude** | **Geographic region** | **Size** |
| --- | --- | --- | --- | --- | --- | --- |
| 1 | Albania |  | 20.17 | 41.15 | South-East | 397 |
| 2 | Albania | AndonPoci | 20.10 | 40.17 | South-East | 19 |
| 3 | Albania | Dukasi | 19.63 | 40.67 | South-East | 39 |
| 4 | Albania | Tirana | 19.82 | 41.33 | South-East | 30 |
| 5 | Austria | Graz | 15.44 | 47.07 | Central | 65 |
| 6 | Austria | Oberöstereich | 13.87 | 48.12 | Central | 178 |
| 7 | Austria | Salzburg | 13.04 | 47.80 | Central | 176 |
| 8 | Austria | Tyrol | 11.53 | 47.20 | Central | 230 |
| 9 | Belarus | Baranavičy | 26.03 | 53.14 | East | 52 |
| 10 | Belarus | Haradok | 29.99 | 55.47 | East | 36 |
| 11 | Belarus | Ivanava | 25.54 | 52.15 | East | 35 |
| 12 | Belarus | Klimavičy | 31.96 | 53.62 | East | 32 |
| 13 | Belarus | Krupki | 29.15 | 54.32 | East | 22 |
| 14 | Belarus | Maladziečna | 26.87 | 54.31 | East | 27 |
| 15 | Belarus | Miadziel | 26.94 | 54.87 | East | 42 |
| 16 | Belarus | Pinsk | 26.10 | 52.12 | East | 117 |
| 17 | Belarus | Polack | 28.79 | 55.49 | East | 26 |
| 18 | Belarus | Smarhoń | 26.42 | 54.49 | East | 45 |
| 19 | Belarus | Svietlahorsk | 29.74 | 52.64 | East | 40 |
| 20 | Belarus | Vietka | 31.18 | 52.56 | East | 15 |
| 21 | Belgium | Antwerpen | 4.68 | 51.24 | West | 302 |
| 22 | Belgium |  | 4.23 | 51.10 | West | 113 |
| 23 | Belgium | Leuven | 4.70 | 50.88 | West | 113 |
| 24 | Belgium | Limburg | 5.37 | 51.07 | West | 58 |
| 25 | Belgium | Oost-Vlaanderen | 3.62 | 51.02 | West | 88 |
| 26 | Belgium | Vlaams-Brabant | 4.57 | 50.86 | West | 158 |
| 27 | Belgium | Walloon | 5.13 | 50.40 | West | 47 |
| 28 | Belgium | West-Vlaanderen | 3.15 | 51.05 | West | 106 |
| 29 | Bosnia and Herzegowina | Doboj-Banja Luka-Bjeljina | 18.08 | 44.75 | South-East | 31 |
| 30 | Bosnia and Herzegowina | Mostar | 17.81 | 43.34 | South-East | 34 |
| 31 | Bosnia and Herzegowina | Sarajevo | 18.42 | 43.86 | South-East | 35 |
| 32 | Bulgaria |  | 25.49 | 42.73 | South-East | 122 |
| 33 | Croatia | Central Croatia | 16.78 | 45.48 | South-East | 220 |
| 34 | Croatia | East Croatia | 18.8 | 45.29 | South-East | 220 |
| 35 | Croatia | North Croatia | 16.83 | 46.16 | South-East | 220 |
| 36 | Croatia | South Croatia | 17.37 | 43.20 | South-East | 220 |
| 37 | Croatia | West Croatia | 15.23 | 44.87 | South-East | 220 |
| 38 | Czech Republic | Central Bohemia | 14.25 | 50.05 | East | 455 |
| 39 | Czech Republic |  | 15.39 | 49.91 | East | 169 |
| 40 | Czech Republic | Hradec Kralove | 15.69 | 50.16 | East | 49 |
| 41 | Czech Republic | Karlovy Vary | 12.87 | 50.23 | East | 31 |
| 42 | Czech Republic | Liberec | 14.96 | 50.72 | East | 45 |
| 43 | Czech Republic | Moravia-Silesia | 18.15 | 49.60 | East | 115 |
| 44 | Czech Republic | Olomouc | 17.35 | 49.50 | East | 53 |
| 45 | Czech Republic | Pardubice | 15.94 | 49.93 | East | 84 |
| 46 | Czech Republic | Plzen | 13.23 | 49.65 | East | 62 |
| 47 | Czech Republic | Southern Bohemia | 14.57 | 49.09 | East | 111 |
| 48 | Czech Republic | Southern Moravia | 16.46 | 49.06 | East | 216 |
| 49 | Czech Republic | Usti nad Labem | 13.89 | 50.62 | East | 87 |
| 50 | Czech Republic | Vysocina | 15.68 | 49.32 | East | 40 |
| 51 | Czech Republic | Zlin | 17.67 | 49.23 | East | 64 |
| 52 | Denmark |  | 9.50 | 56.26 | Central | 248 |
| 53 | Estonia | Tartu | 26.71 | 58.37 | East | 133 |
| 54 | France | Lyon | 4.83 | 45.77 | West | 125 |
| 55 | France | Strasbourg | 7.74 | 48.58 | West | 99 |
| 56 | Germany | Bonn | 7.10 | 50.73 | Central | 90 |
| 57 | Germany | Chemnitz | 12.92 | 50.83 | Central | 833 |
| 58 | Germany | Freiburg | 7.85 | 48.00 | Central | 433 |
| 59 | Germany |  | 13.35 | 51.51 | Central | 30 |
| 60 | Germany | Greifswald | 13.39 | 54.10 | Central | 208 |
| 61 | Germany | Halle | 11.97 | 51.48 | Central | 234 |
| 62 | Germany | Magdeburg | 11.64 | 52.13 | Central | 283 |
| 63 | Germany | Mainz | 8.27 | 50.00 | Central | 104 |
| 64 | Germany | Muenster | 7.63 | 51.96 | Central | 196 |
| 65 | Germany | Rostock | 12.13 | 54.09 | Central | 774 |
| 66 | Greece | Central Greece | 22.26 | 38.55 | South-East | 14 |
| 67 | Greece | Chios | 26.02 | 38.40 | South-East | 16 |
| 68 | Greece | Crete | 24.92 | 35.31 | South-East | 8 |
| 69 | Greece | Epirus | 20.85 | 39.64 | South-East | 14 |
| 70 | Greece |  | 21.82 | 39.07 | South-East | 182 |
| 71 | Greece | Macedonia | 22.69 | 40.79 | South-East | 28 |
| 72 | Greece | Northern Greece | 23.53 | 40.98 | South-East | 191 |
| 73 | Greece | Peloponnes | 22.27 | 37.40 | South-East | 18 |
| 74 | Greece | Thessaly | 22.41 | 39.66 | South-East | 15 |
| 75 | Greece | Thrace | 25.64 | 40.96 | South-East | 41 |
| 76 | Hungary | Fülöpszállás | 19.24 | 46.82 | East | 23 |
| 77 | Hungary |  | 19.50 | 47.16 | East | 236 |
| 78 | Hungary | Szeged | 20.15 | 46.25 | East | 100 |
| 79 | Iceland |  | -19.02 | 64.96 | North | 100 |
| 80 | Ireland |  | -8.24 | 53.41 | West | 155 |
| 81 | Italy | Alcamo | 12.96 | 37.98 | South | 23 |
| 82 | Italy | Belvedere | 16.89 | 39.21 | South | 27 |
| 83 | Italy | Biella Piedmont | 8.06 | 45.56 | South | 80 |
| 84 | Italy | Bologna | 11.35 | 44.49 | South | 51 |
| 85 | Italy | Brescia | 10.22 | 45.54 | South | 106 |
| 86 | Italy | Caccamo | 13.67 | 37.93 | South | 19 |
| 87 | Italy | Catania | 15.09 | 37.50 | South | 17 |
| 88 | Italy | Catanzaro | 16.60 | 38.94 | South | 59 |
| 89 | Italy | Cosenza | 16.27 | 39.33 | South | 37 |
| 90 | Italy | La Spezia | 9.83 | 44.11 | South | 46 |
| 91 | Italy | Liguria | 9.06 | 44.44 | South | 81 |
| 92 | Italy | Lombardy | 9.96 | 45.66 | South | 182 |
| 93 | Italy | Marche | 13.01 | 43.33 | South | 162 |
| 94 | Italy | Mazara del Vallo | 12.59 | 37.65 | South | 25 |
| 95 | Italy | Modena | 10.92 | 44.65 | South | 130 |
| 96 | Italy | Northern Sardinia | 8.55 | 40.70 | South | 100 |
| 97 | Italy | Offida | 13.70 | 42.94 | South | 38 |
| 98 | Italy | Pantelleria | 11.94 | 36.83 | South | 21 |
| 99 | Italy | Piazza Armerina | 14.37 | 37.38 | South | 30 |
| 100 | Italy | Puglia | 16.73 | 41.01 | South | 168 |
| 101 | Italy | Ragusa | 14.73 | 36.93 | South | 29 |
| 102 | Italy | Ravenna | 12.20 | 44.42 | South | 384 |
| 103 | Italy | Reggio di Calabria | 15.68 | 38.17 | South | 97 |
| 104 | Italy | Rimini | 12.56 | 44.06 | South | 98 |
| 105 | Italy | San Giorgio La Molara | 14.93 | 41.27 | South | 31 |
| 106 | Italy | Santa Ninfa | 12.88 | 37.77 | South | 34 |
| 107 | Italy | Sardinia | 9.10 | 40.03 | South | 56 |
| 108 | Italy | Sciacca | 13.08 | 37.51 | South | 20 |
| 109 | Italy | Sicily | 14.13 | 37.53 | South | 314 |
| 110 | Italy | Trapani | 12.52 | 38.02 | South | 72 |
| 111 | Italy | Trino Piedmont | 8.30 | 45.19 | South | 46 |
| 112 | Italy | Troina | 14.59 | 37.79 | South | 21 |
| 113 | Italy | Tuscany | 11.03 | 43.36 | South | 218 |
| 114 | Italy | Udine | 13.23 | 46.06 | South | 47 |
| 115 | Italy | Umbria | 12.58 | 42.99 | South | 51 |
| 116 | Italy | Val Marecchia | 12.52 | 44.06 | South | 65 |
| 117 | Italy | Veneto | 11.86 | 45.74 | South | 120 |
| 118 | Italy | Verona | 11.00 | 45.44 | South | 153 |
| 119 | Lithuania |  | 23.88 | 55.17 | East | 194 |
| 120 | Macedonia | Krusevo | 21.25 | 41.38 | South-East | 43 |
| 121 | Macedonia |  | 21.75 | 41.61 | South-East | 250 |
| 122 | Macedonia | Stip | 22.19 | 41.74 | South-East | 65 |
| 123 | Netherlands | Friesland | 5.62 | 53.15 | West | 44 |
| 124 | Netherlands | Groningen | 6.57 | 53.21 | West | 48 |
| 125 | Netherlands | Leiden | 4.49 | 52.16 | West | 97 |
| 126 | Netherlands | Limburg | 5.90 | 51.26 | West | 50 |
| 127 | Netherlands |  | 5.29 | 52.13 | West | 87 |
| 128 | Netherlands | Noord-Brabant | 5.23 | 51.48 | West | 122 |
| 129 | Netherlands | Zeeland | 3.79 | 51.49 | West | 46 |
| 130 | Norway | Bergen | 5.33 | 60.39 | North | 93 |
| 131 | Norway | Central Norway | 10.23 | 63.36 | North | 317 |
| 132 | Norway | Eastern Norway | 12.08 | 61.26 | North | 493 |
| 133 | Norway | Northern Norway | 23.10 | 69.57 | North | 377 |
| 134 | Norway | Southern Norway | 7.19 | 58.25 | North | 76 |
| 135 | Norway | Western Norway | 6.32 | 62.56 | North | 301 |
| 136 | Poland | Bialystok | 23.17 | 53.13 | East | 595 |
| 137 | Poland | Bydgoszcz | 18.01 | 53.12 | East | 411 |
| 138 | Poland | Gdansk | 18.65 | 54.35 | East | 942 |
| 139 | Poland | Limanowa | 20.42 | 49.71 | East | 53 |
| 140 | Poland | Lublin | 22.57 | 51.25 | East | 246 |
| 141 | Poland | Northern Poland | 18.06 | 54.50 | East | 142 |
| 142 | Poland | Nowy Sacz | 20.72 | 49.62 | East | 114 |
| 143 | Poland | Nowy Targ | 20.03 | 49.48 | East | 52 |
| 144 | Poland | South Eastern Poland | 21.75 | 49.69 | East | 161 |
| 145 | Poland | Southern Poland | 19.94 | 49.83 | East | 380 |
| 146 | Poland | Suwalki | 22.93 | 54.11 | East | 82 |
| 147 | Poland | Szczecin | 14.55 | 53.43 | East | 105 |
| 148 | Poland | Zakopane | 19.95 | 49.3 | East | 7 |
| 149 | Portugal | Azores | -25.62 | 37.79 | West | 68 |
| 150 | Portugal | Central Portugal | -9.08 | 38.44 | West | 792 |
| 151 | Portugal | Madeira | -16.90 | 32.65 | West | 99 |
| 152 | Portugal | Northern Portugal | -8.37 | 41.09 | West | 565 |
| 153 | Portugal | Southern Portugal | -8.35 | 38.11 | West | 112 |
| 154 | Romania | Constanta | 28.62 | 44.16 | East | 36 |
| 155 | Romania | Corund | 25.18 | 46.48 | East | 98 |
| 156 | Romania | Kogalniceanu | 28.45 | 44.37 | East | 42 |
| 157 | Romania | Lunca de Sus | 25.97 | 46.54 | East | 84 |
| 158 | Romania | Miercurea Ciuc | 25.80 | 46.37 | East | 92 |
| 159 | Romania | Moldavia | 26.94 | 46.20 | East | 40 |
| 160 | Bihor | Oradea, Bihor | 22.05 | 47.06 | East | 73 |
| 161 | Bihor | Palota, Bihor | 21.82 | 47.07 | East | 32 |
| 162 | Romania | Ploiesti | 26.02 | 44.94 | East | 36 |
| 163 | Romania |  | 24.97 | 45.94 | East | 104 |
| 164 | Bihor | Sinteu, Bihor | 22.36 | 47.08 | East | 35 |
| 165 | Romania | Transylvania | 23.18 | 46.39 | East | 14 |
| 166 | Romania | Wallachia | 25.09 | 44.71 | East | 96 |
| 167 | Russian Federation | Archangelsk | 40.57 | 64.54 | East | 42 |
| 168 | Russian Federation | Belgorod | 36.61 | 50.60 | East | 37 |
| 169 | Russian Federation | Brjansk | 34.30 | 53.31 | East | 43 |
| 170 | Russian Federation | Ivanowo | 40.97 | 56.99 | East | 40 |
| 171 | Russian Federation | Kaluga | 36.27 | 54.51 | East | 36 |
| 172 | Russian Federation | Mineralnye Vody | 43.14 | 44.21 | East | 11 |
| 173 | Russian Federation | Novgorod | 31.27 | 58.54 | East | 127 |
| 174 | Russian Federation | Orel | 36.07 | 52.97 | East | 73 |
| 175 | Russian Federation | Pskov | 28.33 | 57.82 | East | 40 |
| 176 | Russian Federation | Smolensk | 32.05 | 54.78 | East | 43 |
| 177 | Russian Federation | Stawropol | 42.02 | 45.10 | East | 64 |
| 178 | Russian Federation | Tambov | 41.42 | 52.72 | East | 48 |
| 179 | Russian Federation | Tver | 35.92 | 56.86 | East | 43 |
| 180 | Russian Federation | Vladimir | 40.41 | 56.13 | East | 50 |
| 181 | Russian Federation | Vologda | 39.88 | 59.22 | East | 40 |
| 182 | Russian Federation | Volot | 30.70 | 57.93 | East | 32 |
| 183 | Serbia | Novi Sad | 19.83 | 45.25 | East | 615 |
| 184 | Slovakia | Bratislava | 17.11 | 48.15 | East | 164 |
| 185 | Slovakia | Eastern Slovakia | 21.24 | 49.00 | East | 629 |
| 186 | Slovakia |  | 19.39 | 48.79 | East | 80 |
| 187 | Spain | Alava | -2.70 | 42.91 | West | 33 |
| 188 | Spain | Alpujarra de la Sierra | -3.16 | 36.98 | West | 50 |
| 189 | Spain | Andalucía/Extremadura | -6.04 | 37.44 | West | 390 |
| 190 | Spain | Asturias | -5.85 | 43.27 | West | 90 |
| 191 | Spain | Basque Country | -2.56 | 43.15 | West | 365 |
| 192 | Spain | Biscay | -2.68 | 43.22 | West | 87 |
| 193 | Spain | Caceres | -6.37 | 39.48 | West | 91 |
| 194 | Spain | Cantabria | -4.00 | 43.14 | West | 101 |
| 195 | Spain | Granada | -3.60 | 37.18 | West | 180 |
| 196 | Spain | Guipuzcoa | -2.22 | 43.07 | West | 19 |
| 197 | Spain | Huelva | -6.95 | 37.26 | West | 167 |
| 198 | Spain | Ibiza | 1.57 | 39.16 | West | 96 |
| 199 | Spain | Majorca | 2.98 | 39.73 | West | 91 |
| 200 | Spain | Pyrenees | 0.17 | 42.55 | West | 134 |
| 201 | Spain | Santiago de Compostela | -8.55 | 42.88 | West | 103 |
| 202 | Sweden | Blekinge | 15.31 | 56.13 | North | 41 |
| 203 | Sweden | Gotland | 18.73 | 57.64 | North | 40 |
| 204 | Sweden | Östergötland/Jönköping | 14.16 | 57.78 | North | 40 |
| 205 | Sweden | Skaraborg | 15.37 | 60.31 | North | 44 |
| 206 | Sweden |  | 14.68 | 59.29 | North | 400 |
| 207 | Sweden | Uppsala | 17.64 | 59.86 | North | 54 |
| 208 | Sweden | Värmland | 13.08 | 59.89 | North | 42 |
| 209 | Sweden | Västerbotten | 18.20 | 64.72 | North | 41 |
| 210 | Switzerland | Bern | 7.45 | 46.95 | Central | 91 |
| 211 | Switzerland | Lausanne | 6.63 | 46.52 | Central | 108 |
| 212 | Switzerland |  | 8.23 | 46.82 | Central | 150 |
| 213 | Ukraine | Lugansk | 38.44 | 48.90 | East | 88 |
| 214 | Ukraine | Uzhgorod | 22.31 | 48.62 | East | 81 |

**References**

[1] Roewer L, Croucher PJ, Willuweit S, Lu TT, Kayser M, et al. (2005) Signature of recent historical events in the European Y-chromosomal STR haplotype distribution. Human Genetics 116: 279-291.
